# Diastereoselective Three-Component Reactions of Chiral Nickel(II) Glycinate for Convenient Synthesis of Novel α-Amino-β-Substituted-γ,γ-Disubstituted Butyric Acids

**DOI:** 10.3390/molecules19010826

**Published:** 2014-01-10

**Authors:** Rui Zhou, Li Guo, Cheng Peng, Gu He, Liang Ouyang, Wei Huang

**Affiliations:** 1 State Key Laboratory of Biotherapy, West China Hospital, and West China School of Pharmacy, Sichuan University, Chengdu 610041, Sichuan, China; E-Mails: sklb_zhourui@126.com (R.Z.); guoli@scu.edu.cn (L.G.); ouyangliang@scu.edu.cn (L.O.); 2 State Key Laboratory Breeding Base of Systematic Research, Development and Utilization of Chinese Medicine, Chengdu University of Traditional Chinese Medicine, Chengdu 610041, Sichuan, China; E-Mail: pengchengchengdu@126.com

**Keywords:** multi-component reaction, nickel(II), glycine, diastereoselectivity, unnatural amino acids

## Abstract

The convenient, high yielding and diastereoselective synthesis of α-amino-β-substituted-γ,γ-disubstituted butyric acid derivatives was carried out by a three-component tandem reaction of a chiral equivalent of nucleophilic glycine. The reaction was performed smoothly under mild conditions and enabled the construction of two or three adjacent chiral centers in one step, thus affording a novel and convenient route to α-amino-β-substituted-γ,γ-disubstituted butyric acid derivatives.

## 1. Introduction

**Figure 1:**
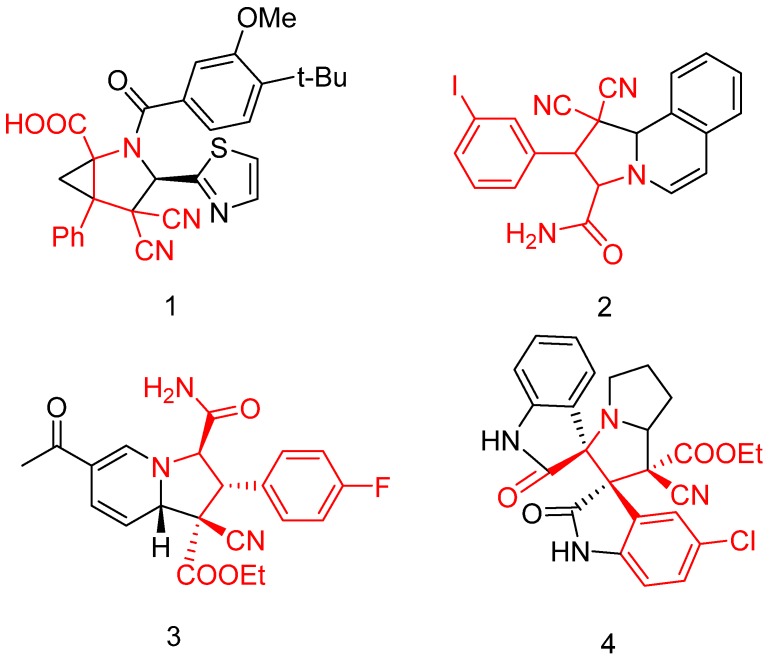
Structures of some biologically important compounds containing α-amino-β-substituted γ,γ- disubstituted butyric acid motifs.

Chiral α-amino-γ,γ-disubstituted fragments are frequently found in various bioactive compounds, such as anti-infective agents (compound **1**), anti-tuberculosis agents (compound **2**), modulators of RNA binding proteins (compound **3**) and compositions for specific inhibition of protein splicing by small molecules, and used in the treatment of tuberculosis and other conditions (compound **4**) (Figure 1) [1,2,3,4]. Catalytic diastereoselective synthesis of these chiral α-amino-β-substituted butyric acid derivatives rely on many reactions, for example, addition of α,β-unsaturated acyloxazolidinones, then the removal of the oxazolidinone portions [5], cycloaddition of chiral nitrones with (*E*)-1,4-dichlorobut-2-ene, followed by acid-catalyzed hydrolysis and then by amide hydrolysis [6], but the Michael addition should be considered the main method to get these products when a chiral equivalent of glycine is used. Indeed, several examples of such reactions using chiral auxiliaries have been reported [7,8,9,10,11,12,13,14,15]. However, to our knowledge, there are no reports about the synthesis of chiral α-amino-β-substituted-γ,γ-disubstituted butyric acid derivatives.

The chiral Ni(II) complex of the Schiff base of glycine (abbreviated as (*S*)-BPB) is commonly used in the asymmetric syntheses of unnatural amino acids. Product mixtures with a high excess of the (*S*)-amino acid diastereomer are always generated by the addition using (*S*)-BPB as a ligand [16,17,18]. The products can be easily isolated by column chromatography, and decomposed by acid to get chiral pure amino acids. Moreover, the recovery of (*S*)-BPB can be high (up to 85%). To the best of our knowledge, a variety of glutamic acid and proline derivatives with a high *ee* values can be synthesized through Michael additions of activated olefins to Ni(II) glycinate [19,20,21]. Recently, Liu *et al.* reported the efficient synthesis of β-substituted α,γ-diaminobutyric acid derivatives using asymmetric Michael addition reactions of chiral nickel(II) glycinate with nitroalkenes [22,23,24,25,26,27], Schneider *et al.* reported the stereoselectivity synthesis of γ-carboxyglutamic acids using asymmetric Michael addition reactions of chiral copper(II) glycinate with di-*tert*-butyl methylenemalonate [28]. This report focuses on the synthesis of α-amino-β-substituted-γ,γ-disubstituted butyric acid derivatives through the reaction of aromatic aldehydes, a chiral Ni(II) glycinate complex, and an α-carbanion of two electron-withdrawing groups (malononitrile or ethyl cyanoacetate) as a continuation of our previous research on new methods for the preparation of potentially bioactive compounds by multi-component reactions [29,30,31,32,33]. In the process, two carbon-carbon bonds were constructed and two or three chiral centers were generated in a convenient one-pot reaction with a high stereoselectivity.

## 2. Results and Discussion

The Michael addition reaction was considered as an effective way to get the products. Firstly, the optimization of the reaction conditions was undertaken using a model reaction of chiral nickel(II) glycinate with 2-benzylidenemalononitrile (Table 1). The reaction with 1,8-diazabicyclo[5.4.0]undec-7-ene (DBU), triethylamine(TEA), 4-methylmorpholine (NMM) and piperidine gave a little lower diastereoselectivities (Entries 1–5, Table 1) than Hunig’s base (DIEA) did, and all the reactions gave satisfactory yields, except the one in NMM.

**Table 1:** Optimization of the reaction conditions ^a^. 
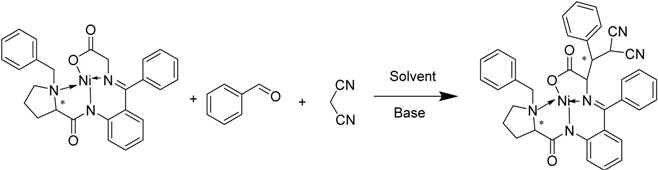

Entry	Base	Solvent	Yield (%) ^b^	*de* ^c^
1	DBU ^d^	CH_3_CN	94	88%
2	TEA	CH_3_CN	97	94%
3	DIEA	CH_3_CN	94	96%
4	NMM	CH_3_CN	63	92%
5	Piperidine	CH_3_CN	89	96%
6	DIEA	DMF	99	88%
7	DIEA	EA	98	80%
8	DIEA	MeOH	99	88%
9	DIEA	DCM	97	58%
10	DIEA	dioxane	91	97%
11	DIEA	CHCl_3_	59	90%
12	DIEA	DMSO	91	76%

^a^ All the reactions were conducted at ambient temperature; ^b^ Yield of the major products after silica gel column chromatography; ^c^ Determined by HPLC analysis; ^d^ DBU was used in 0.15 equiv.; and other bases were used in 3 equiv.

The reaction proceeded smoothly in most of the solvents tested, although the one in chloroform gave a bad yield and the one in dichloromethane (DCM) gave poor diastereoselectivity. Good diastereoselectivities and yields were observed with the use of acetonitrile, *N,N*-dimethylformamide (DMF), ethyl acetate (EA), methanol, dioxane and dimethyl sulfoxide (DMSO). Above all, diastereoselectivity was not obviously influenced by the kind of the bases used, and polar solvents seemed to be better than nonpolar ones. As 2-benzylidenemalononitrile can be easily generated from benzaldehyde and malononitrile under alkaline conditions, domino reaction of these three components was thought to be feasible. In fact, TLC showed that when these three components were mixed together under basic conditions, benzaldehyde first reacted quickly with malononitrile, then added to the nickel(II) glycinate and the product 7a appeared. The results showed no big difference with those in Table 1, so the substrate scope was investigated using DIEA as the base and dioxane as the solvent (entry 11, Table 1) without further optimization.

**Figure 2:**
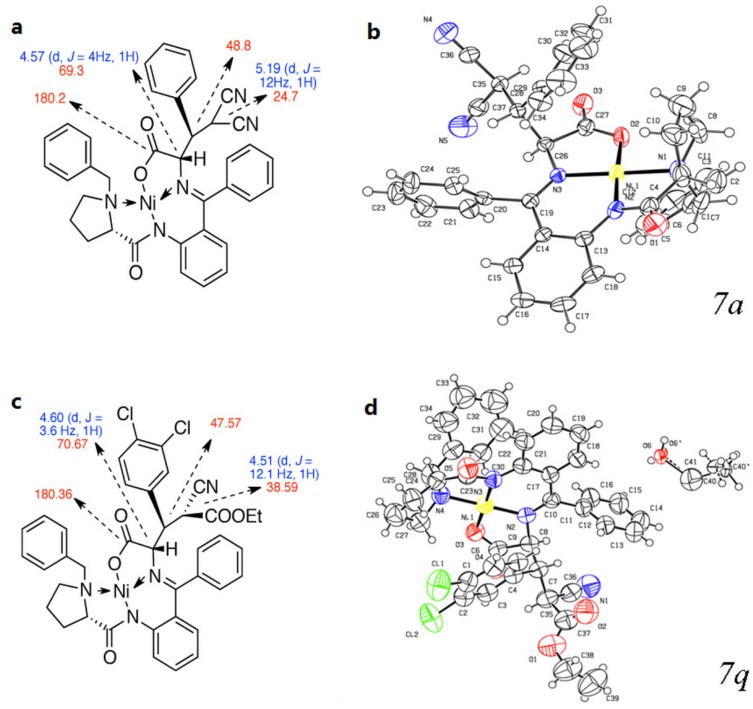
(**a**) Selected ^1^H- and ^13^C-NMR chemical shifts of (*S*,2*S*,3*R*)-**7a**; (**b**) Single crystal X-ray diffraction study of (*S*,2*S*,3*R*)-**7a**; (**c**) Selected ^1^H- and ^13^C-NMR chemical shifts of (*S*,2*S*,3*R*,4*S*)-**7q** and (**d**) Single crystal X-ray diffraction study of (*S*,2*S*,3*R*,4*S*)-**7q**.

The aromatic aldehydes with substituents at different positions were introduced into this reaction (Table 2). Whether functionalized with either electron-withdrawing or electron-donating groups, these aldehydes gave the products in good to high yields. As the result obtained with malononitrile was inspiring, ethyl cyanoacetate was introduced into the reaction, and gave a satisfactory result, so the reactions with malononitrile and ethyl cyanoacetate could be looked as two series. The results of the ethyl cyanoacetate series seemed a little better than the malononitrile series on average, despite the fact three chiral centers are newly generated. In both series, the naphthyl-functionalized aldehydes had the best diastereoselectivities (Table 2, enties 9 and 10), and *ortho*-functionalized aromatic aldehydes gave relatively high yields and diastereoselectivities (Table 2, entries 12, 14 and 18). The results were quite different in this two series when *t*-Bu- and 3-Cl-substituted substrates were involved (Table 2, entries 2 and 22, 3 and 15). However, furaldehyde and thienaldehyde were not tolerated (Table 2, enties 13 and 14). To elucidate the relative and absolute configurations of the products, X-ray single crystal structures of (*S*,2*S*,3*R*)-**7a** (CCDC 951535) and (*S*,2*S*,3*R*,4*S*)-**7q** (CCDC 949234) are given below (Figure 2).

To further confirm the structure, diastereoselectivity and regioselectivity, detailed NMR spectral and X-ray analyses were carried out. The structures proposed for all products were in agreement with their NMR spectra, as discussed for compounds **7a** and **7q** as examples. In the ^1^H-NMR spectrum of **7a** and **7q**, the α-C proton of glycine exhibited double(d) peaks at δ 4.57 (d, *J* = 4 Hz, 1H) and δ 4.60 (d, *J* = 3.6 Hz, 1H), respectively. The α-C proton of malononitrile in **7a** appeared as a doublet at δ 5.19 (d, *J* = 12 Hz, 1H), and the corresponding proton of ethyl cyanoacetate in **7q** appeared as a doublet at δ 4.51 (d, *J* = 12.1 Hz, 1H). The relative configuration of these structures should be as same as compound **7a** and **7q** shown in Figure 2a,c, the configurations were further confirmed by the X-ray study of single crystals (Figure 2b,d). The ^13^C-NMR of compound **4b** supported the proposed structure as well.

**Scheme 1:**
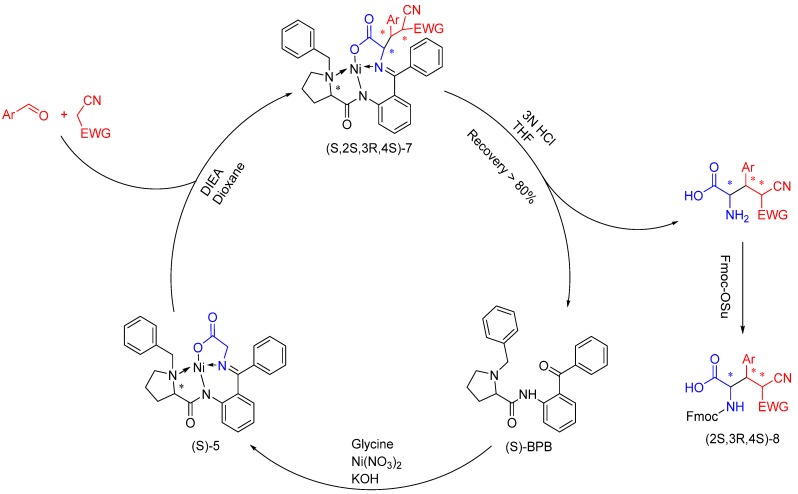
Asymmetric domino reactions of chiral nickel(II) glycinate (*S*)-5 with aromatic aldehydes and α-carbanion.

A plausible mechanism for the high diastereoselectivity of the reaction could be explained as follows (Scheme 1): malononitrile or cyanide ethyl acetate first reacted with aromatic aldehyde, and the intermediate formed continued to react with the complex. When (*S*)-*N*-benzylproline was used, the benzyl group was on a certain side of this complex, so the steric hindrance was large on this side, and the intermediate would prefer attacking from the other side. Still, steric hindrance from the phenyl groups of the intermediate could contribute to the diasteroselectivity, this may explain why naphthaldehydes provided a high *de* value. As the diastereoselectivity was mainly controlled by the substrates, the reaction was easy to carry, making it a convenient way to get chiral α-amino-β-substituted-γ,γ-disubstituted butyric acid derivatives.

**Table 2:** Asymmetric Michael reactions of chiral nickel(II) glycinate (*S*)-5 with aromatic aldehydes and α-carbanions ^a^. 
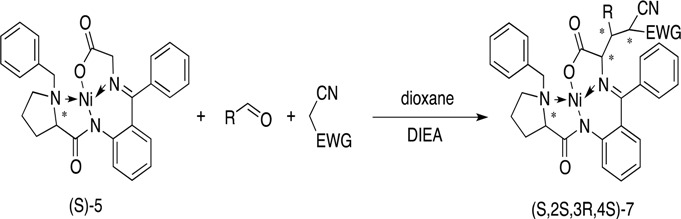

Entry	Product	R	EWG	Yield (%) ^b^	*de* ^c^
1	(*S*,2*S*,3*R*)-**7a**	Ph	CN	91	97%
2	(*S,2S,3R*)-**7b**	4-(*t*-Bu)-C_6_H_4_	CN	86	>99%
3	(*S,2S,3R*)-**7c**	3-Cl-C_6_H_4_	CN	52	90%
4	(*S,2S,3R*)-**7d**	4-F-C_6_H_4_	CN	83	93%
5	(*S,2S,3R*)-**7e**	4-Br-C_6_H_4_	CN	44	98%
6	(*S,2S,3R*)-**7f**	3,4-di-Cl-C_6_H_3_	CN	84	97%
7	(*S,2S,3R*)-**7g**	3-Br-C_6_H_4_	CN	38	95%
8	(*S,2S,3R*)-**7h**	3-OMe-C_6_H_4_	CN	82	>99%
9	(*S,2S,3R*)-**7i**	2-naphthyl	CN	80	98%
10	(*S,2S,3R*)-**7j**	1-naphthyl	CN	26	98%
11	(*S,2S,3R*)-**7k**	3-OH-C_6_H_4_	CN	46	98%
12	(*S,2S,3R*)-**7l**	2-F-4-Br-C_6_H_3_	CN	90	>99%
13	(*S,2S,3R,4S*)-**7m**	Ph	COOEt	78	98%
14	(*S,2S,3R,4S*)-**7n**	2-Br-C_6_H_4_	COOEt	88	>99%
15	(*S,2S,3R,4S*)-**7o**	3-Cl-C_6_H_4_	COOEt	89	97%
16	(*S,2S,3R,4S*)-**7p**	4-F-C_6_H_4_	COOEt	75	96%
17	(*S,2S,3R,4S*)-**7q**	3,4-di-Cl-C_6_H_3_	COOEt	82	98%
18	(*S,2S,3R,4S*)-**7r**	2,4-di-Cl-C_6_H_3_	COOEt	96	98%
19	(*S,2S,3R,4S*)-**7s**	4-CH_3_-C_6_H_4_	COOEt	76	>99%
20	(*S,2S,3R,4S*)-**7t**	4-OCH_3_-C_6_H_4_	COOEt	77	97%
21	(*S,2S,3R,4S*)-**7u**	4-NO_2_-C_6_H_4_	COOEt	69	97%
22	(*S,2S,3R,4S*)-**7v**	4-(*t*-Bu)-C_6_H_4_	COOEt	51	97%
23	(*S,2S,3R,4S*)-**7w**	1-naphthyl	COOEt	67	>99%
24	(*S,2S,3R*)-**7x**	3-Br-thienyl	CN	NR ^d^	NR ^d^
25	(*S,2S,3R*)-**7y**	4-Me-Furyl	CN	NR ^d^	NR ^d^

^a^ All the reactions were conducted at ambient temperature, 3 equiv. of all the bases were used; ^b^ Yield of the major products after silica gel column chromatography; ^c^ Determined by HPLC analysis; ^d^ Not Reaction.

**Scheme 2:**
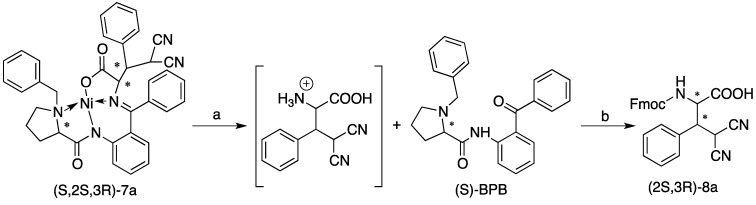
Decomposition of Ni(II) complex **7a** to release product **8a** and recovery of the (*S*)-BPB.

With high diastereoselectivities and mild reaction conditions, the synthesis of (2*S*,3*R*)-**8a** (Scheme 2) was completed by optimizing the metal complex decomposition and Fmoc-protection conditions. Typically, the compound (*S*,2*S*,3*R*)-**7a** was decomposed by heating a suspension in methanol/6N HCl. However, we found that one of the nitrile groups was partly hydrolyzed in this process, so suitable conditions were sought to ensure that the nitrile groups remain inert. When we stirred (*S*,2*S*,3*R*)-**7a** in THF with a 3N concentration HCl at ambient temperature, the complex was decomposed and the nitrile group preserved (Scheme 2). The chiral ligand (*S*)-BPB can be easily recovered quantitatively. (2*S*,3*R*)-**8a** was synthesized after (*S*,2*S*,3*R*)-**7a** was decomposed, the (*S*)-BPB was extracted with ethyl acetate (EA) and the α-amino-β-substituted γ,γ-disubstituted butyric acid product was protected by a Fmoc group. Ultimately, the yield of (*S*,2*R*)-**5a** from (*S*,2*S*,3*R*)-**7a** was 62% over two steps.

## 3. Experimental

### 3.1. General

The reagents (chemicals) were purchased from commercial sources, and used without further purification. Analytical thin layer chromatography (TLC) was GF_254_ (0.15–0.2 mm thickness). The mass spectra and high resolution mass spectra were obtained using Bruker microTOF-Q instrument or TOF-MS instrument. The ^1^H- and ^13^C-NMR spectra have been respectively measured in CDCl_3_ or DMSO-*d_6_* at 400 and 100 MHz using a Bruker Avance III 400 MHz instrument with TMS as an internal standard. Analytical high performance liquid chromatography was carried out using the Waters Alliance 2695 HPLC, using the Chiralpak IA column. The loading loop was 10 μL. The eluting employed was an isocratic mixture of *n*-hexane and *i*-propanol (50:50 respectively) at a flow of 1 mL/min unless stated. Retention times are reported in minutes. The enantiomeric excess was calculated from the integration of the absorption peaks at 220 nm.

### 3.2. Typical Procedure for the Synthesis of (S)-Nickel(II) Complex (**5**) [18]

(*S*)-BPB (1 g, 2.60 mmol), Ni(NO_3_)_2_·6H_2_O (1.52 g, 5.21 mmol) and glycine (976 mg, 13.0 mmol) were stirred in MeOH (50 mL). Then NaH (55%–65% in oil, 1.04 g, 26 mmol) and KOH (437 mg, 7.81 mmol) were added successively. The mixture was refluxed for 2 h then cooled to room temperature and neutralized with acetic acid. After 12 h the precipitate was filtered and washed with ethanol (100 mL), followed by stirring in methane/water (*v*/*v*) 1:2 (200 mL), then filtered to form a red crystalline solid (1.27 g, yield 98%). The complex was sufficiently pure for further use without additional purification.

### 3.3. General Procedure for the Synthesis of Ni(II) (**7**)

The nickel(II) complex of glycine (*S*)-**5** (1.0 equiv.) was dissolved in dioxane, and DIEA (3.0 equiv.), aromatic aldehyde (1.2 equiv.) and malononitrile/ethyl cyanoacetate (1.2 equiv.) was added at room temperature. The mixture was then stirred at room temperature for 12 h, then poured into 10% citric acid solution, extracted with CH_2_Cl_2_ (three times), dried with anhydrous Na_2_SO_4_, concentrated, and purified on silica (petroleum ether/ethyl acetate = 1/1) to give **7** as a red solid.

#### 3.3.1. Ni(II)-(*S*)-BPB/(*2S*,*3R*)-2-Amino-4,4'-dicyano-3-phenylbutyric Acid Schiff Base Complex (**7a**)

Yield = 91%, m.p. 202–204 °C. 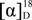 = +1602 (ca. 0.2 g/100 mL, CHCl_3_). ^1^H-NMR (CDCl_3_) δ 8.25 (d, *J* = 8 Hz, 1H), 7.98 (d, *J* = 8 Hz, 2H), 7.68–7.63 (m, 6H), 7.39 (d, *J* = 8 Hz, 1H), 7.30–7.26 (m, 4H), 7.18–7.14 (m, 3H), 6.70 (d, *J* = 4 Hz, 1H), 5.19 (d, *J* = 12 Hz, 1H), 4.57 (d, *J* = 4 Hz, 1H), 4.15 (d, *J* = 12 Hz, 1H), 3.42 (d, *J* = 12 Hz, 1H), 3.29–3.21 (m, 2H), 2.97–2.91 (m, 1H), 2.32–2.20 (m, 1H), 2.06–2.02 (m, 1H), 1.95–1.91 (m, 1H), 1.85–1.83 (m, 1H), 1.56–1.50 (m, 1H). ^13^C-NMR (CDCl_3_) δ 180.2, 176.2, 173.5, 143.4, 133.9, 133.5, 133.2, 132.6, 131.4, 130.9, 130.6, 130.1, 129.9, 129.5, 128.9, 128.9, 127.5, 127.0, 125.5, 123.3, 120.8, 111.5, 111.3, 70.5, 69.7, 63.9, 57.4, 48.8, 29.7, 24.7, 23.1. ESI-MS (*m*/*z*): calcd. 652.2, found 652.2 ([M+H]^+^); HRMS (*m*/*z*): calcd. C_37_H_32_N_5_NiO_3_ for 652.1859, found 652.1857 ([M+H]^+^). HPLC (Chiralpak IA, *n*-hexane/*i*-propanol = 50/50, flow rate 1.0 mL/min, λ = 220 nm), t_major_ = 32.3 min, t_minor_ = 13.4 min, *de* = 97%.

#### 3.3.2. Ni(II)-(*S*)-BPB/(*2S*,*3R*)-2-Amino-4,4'-dicyano-3-(4-tert-butyl)-phenylbutyric Acid Schiff Base Complex (**7b**)

Yield = 86%, m.p. 197–199 °C. 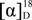 = +1602 (ca. 0.2 g/100 mL, CHCl_3_). ^1^H-NMR (CDCl_3_) δ 8.29 (d, *J* = 8.7 Hz, 1H), 7.95 (d, *J* = 7.5 Hz, 2H), 7.68 (dt, *J* = 13.0, 6.8 Hz, 3H), 7.60 (d, *J* = 8.1 Hz, 2H), 7.41 (d, *J* = 7.2 Hz, 1H), 7.29 (dd, *J* = 14.5, 7.1 Hz, 3H), 7.19 (d, *J* = 7.9 Hz, 3H), 7.16–7.09 (m, 2H), 6.70 (d, *J* = 2.9 Hz, 2H), 5.16 (d, *J* = 12.0 Hz, 1H), 4.58 (d, *J* = 3.4 Hz, 1H), 4.16 (d, *J* = 12.7 Hz, 1H), 3.79 (s, 1H), 3.49 (d, *J* = 12.7 Hz, 1H), 3.28–3.13 (m, 2H), 3.05–2.90 (m, 1H), 2.29–2.13 (m, 1H), 2.12–2.00 (m, 1H), 1.92 (dt, *J* = 27.0, 8.6 Hz, 1H), 1.78 (dt, *J* = 18.9, 9.3 Hz, 1H), 1.54 (s, 1H), 1.46–1.31 (m, 9H). ^13^C-NMR (CDCl_3_) δ 180.2, 176.3, 173.3, 160.9, 143.2, 133.9, 133.4, 133.3, 133.2, 131.4, 130.8, 130.5, 129.4, 128.9, 128.8, 127.4, 127.0, 125.6, 124.1, 123.2, 120.8, 115.2, 111.6, 111.3, 70.6, 69.8, 64.0, 57.6, 55.4, 48.1, 30.5, 24.7, 22.9. ESI-MS (*m*/*z*): calcd. 708.2, found 708.3 ([M+H]^+^); HRMS (*m*/*z*): calcd. C_41_H_40_N_5_NiO_3_ for 708.2485, found 708.2482 ([M+H]^+^). HPLC (Chiralpak IA, *n*-hexane/*i*-propanol = 50/50, flow rate 1.0 mL/min, λ = 220 nm), t_major_ = 30.9 min, t_minor_ = 17.3 min, *de* > 99%.

#### 3.3.3. Ni(II)-(*S*)-BPB/(*2S*,*3R*)-2-Amino-4,4'-dicyano-3-(3-chlorophenyl) Butyric Acid Schiff Base Complex (**7c**)

Yield = 52%, m.p. 211–213 °C. 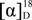 = +1622 (ca. 0.2 g/100 mL, CHCl_3_). ^1^H-NMR (CDCl_3_) δ 8.27 (d, *J* = 8.7 Hz, 1H), 8.00 (d, *J* = 7.5 Hz, 2H), 7.75–7.60 (m, 4H), 7.55 (d, *J* = 15.7 Hz, 1H), 7.42–7.34 (m, 2H), 7.32 (dd, *J* = 14.6, 6.9 Hz, 3H), 7.22–7.09 (m, 4H), 6.70 (d, *J* = 3.7 Hz, 2H), 5.14 (d, *J* = 11.9 Hz, 1H), 4.56 (d, *J* = 3.8 Hz, 1H), 4.15 (d, *J* = 12.7 Hz, 1H), 3.43 (d, *J* = 12.6 Hz, 1H), 3.25 (ddd, *J* = 16.2, 10.7, 5.6 Hz, 2H), 3.02 (dd, *J* = 9.8, 5.5 Hz, 1H), 2.25 (dd, *J* = 18.6, 10.0 Hz, 1H), 2.10–1.98 (m, 3H), 1.70–1.63 (m, 1H). ^13^C-NMR (CDCl_3_) δ 180.2, 175.9, 173.7, 143.4, 136.3, 134.6, 133.9, 133.4, 133.4, 133.2, 131.3, 131.0, 131.0, 130.6, 130.4, 129.5, 129.0, 128.8, 127.4, 127.0, 125.4, 123.3, 120.8, 111.2, 110.9, 70.5, 69.4, 64.0, 57.6, 48.4, 30.7, 29.6, 24.6, 23.0. ESI-MS (*m*/*z*): calcd. 686.1, found 686.2 ([M+H]^+^). HRMS (*m*/*z*): calcd. C_37_H_31_ClN_5_NiO_3_ for 686.1469, found 686.1475 ([M+H]^+^). HPLC (Chiralpak IA, *n*-hexane/*i*-propanol = 50/50, flow rate 1.0 mL/min, λ = 220 nm), t_major_ = 31.0 min, t_minor_ = 13.0 min, *de* = 90%.

#### 3.3.4. Ni(II)-(*S*)-BPB/(*2S*,*3R*)-2-Amino-4,4'-dicyano-3-(4-fluorophenyl)butyric Acid Schiff Base Complex (**7d**)

Yield = 83%, m.p. 207–209 °C. 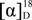 = +1734 (ca. 0.2 g/100 mL, CHCl_3_). ^1^H-NMR (CDCl_3_) δ 8.25 (d, *J* = 8.6 Hz, 1H), 8.00 (d, *J* = 7.4 Hz, 2H), 7.73–7.62 (m, 3H), 7.34 (ddd, *J* = 14.8, 13.7, 7.2 Hz, 7H), 7.21–7.09 (m, 3H), 6.73–6.66 (m, 2H), 5.16 (d, *J* = 12.0 Hz, 1H), 4.55 (d, *J* = 3.7 Hz, 1H), 4.15 (d, *J* = 13.1 Hz, 1H), 3.48–3.37 (m, 1H), 3.32–3.22 (m, 2H), 3.02–2.90 (m, 1H), 2.33–2.23 (m, 1H), 2.13–2.05 (m, 1H), 1.97 (dd, *J* = 13.3, 7.4 Hz, 2H), 1.67–1.61 (m, 1H). ^13^C-NMR (CDCl_3_) δ 180.2, 176.1, 173.6, 165.1, 162.6, 143.3, 133.9, 133.4, 133.3, 133.2, 131.3, 131.2, 130.9, 130.6, 129.5, 129.0, 128.8, 128.4, 128.4, 127.3, 127.0, 125.4, 123.3, 120.8, 117.1, 116.9, 111.3, 111.0, 70.5, 69.6, 64.0, 57.5, 48.1, 30.6, 24.7, 22.9. ESI-MS (*m*/*z*): calcd. 670.2, found 670.2 ([M+H]^+^). HRMS (*m*/*z*): calcd. C_37_H_31_FN_5_NiO_3_ for 670.1764, found 670.1801 ([M+H]^+^). HPLC (Chiralpak IA, *n*-hexane/*i*-propanol = 50/50, flow rate 1.0 mL/min, λ = 220 nm), t_major_ = 26.0 min, t_minor_ = 10.1 min, *de* = 93%.

#### 3.3.5. Ni(II)-(*S*)-BPB/(*2S*,*3R*)-2-amino-4,4'-dicyano-3-(4-bromophenyl) Butyric Acid Schiff Base Complex (**7e**)

Yield = 44%, m.p. 197–199 °C. 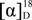 = +1972 (ca. 0.2 g/100 mL, CHCl_3_). ^1^H-NMR (CDCl_3_) δ 8.23 (d, *J* = 8.7 Hz, 1H), 8.01 (d, *J* = 7.4 Hz, 2H), 7.78–7.58 (m, 3H), 7.38 (d, *J* = 6.8 Hz, 1H), 7.29 (dd, *J* = 14.9, 7.4 Hz, 3H), 7.25–7.08 (m, 7H), 6.70 (d, *J* = 13.7 Hz, 2H), 5.14 (d, *J* = 12.0 Hz, 1H), 4.53 (d, *J* = 3.2 Hz, 1H), 4.15 (d, *J* = 12.7 Hz, 1H), 3.40 (d, *J* = 12.6 Hz, 1H), 3.31–3.18 (m, 2H), 2.99 (dd, *J* = 10.5, 6.0 Hz, 1H), 2.23 (dt, *J* = 24.1, 12.1 Hz, 1H), 2.13–2.02 (m, 1H), 2.02–1.87 (m, 2H), 1.55 (dd, *J* = 12.1, 6.5 Hz, 1H). ^13^C-NMR (CDCl_3_) δ 180.2, 176.3, 173.3, 160.9, 143.2, 133.9, 133.4, 133.3, 133.2, 131.4, 130.8, 130.5, 129.4, 128.9, 128.8, 127.4, 127.0, 125.6, 124.1, 123.2, 120.8, 115.2, 111.6, 111.3, 70.6, 69.8, 64.0, 57.6, 55.4, 48.1, 30.5, 24.7, 22.9. ESI-MS (*m*/*z*): calcd. 730.1, found 730.2 ([M+H]^+^). HRMS (*m*/*z*): calcd. C_37_H_31_BrN_5_NiO_3_ for 730.0966, found 730.0978 ([M+H]^+^). HPLC (Chiralpak IA, *n*-hexane/*i*-propanol = 50/50, flow rate 1.0 mL/min, λ = 220 nm), t_major_ = 32.0 min, t_minor_ = 15.3 min, *de* = 98%.

#### 3.3.6. Ni(II)-(*S*)-BPB/(*2S*,*3R*)-2-Amino-4,4'-dicyano-3-(3,4-dichlorophenyl) Butyric Acid Schiff Base Complex (**7f**)

Yield = 84%, m.p. 213–215 °C. 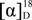 = +1660 (ca. 0.2 g/100 mL, CHCl_3_). ^1^H-NMR (CDCl_3_) δ 8.27 (d, *J* = 8.7 Hz, 1H), 8.01 (d, *J* = 7.4 Hz, 2H), 7.69 (dd, *J* = 17.4, 7.8 Hz, 4H), 7.44 (s, 1H), 7.38 (d, *J* = 6.3 Hz, 1H), 7.31 (t, *J* = 7.4 Hz, 2H), 7.18 (dd, *J* = 15.9, 8.3 Hz, 2H), 7.09 (t, *J* = 7.8 Hz, 2H), 6.71 (d, *J* = 14.3 Hz, 2H), 5.15 (d, *J* = 11.9 Hz, 1H), 4.53 (s, 1H), 4.12 (dd, *J* = 17.0, 9.9 Hz, 1H), 3.42 (d, *J* = 12.6 Hz, 1H), 3.30 (t, *J* = 8.3 Hz, 1H), 3.22 (d, *J* = 11.9 Hz, 1H), 2.99 (d, *J* = 5.8 Hz, 1H), 2.33 (dd, *J* = 20.4, 8.8 Hz, 1H), 2.02 (qd, *J* = 13.8, 6.8 Hz, 3H), 1.77–1.66 (m, 1H). ^13^C-NMR (CDCl_3_) δ 180.2, 175.8, 173.8, 143.4, 135.0, 134.6, 134.0, 133.5, 133.3, 132.7, 131.7, 131.3, 131.1, 130.7, 129.6, 129.0, 128.9, 127.2, 126.9, 125.2, 123.3, 120.8, 111.1, 110.6, 70.5, 69.3, 64.1, 57.8, 48.0, 30.7, 24.4, 22.8. ESI-MS (*m*/*z*): calcd. 720.1, found 720.2 ([M+H]^+^); HRMS (*m*/*z*): calcd. C_37_H_30_Cl_2_N_5_NiO_3_ for 720.1079, found 720.1090 ([M+H]^+^). HPLC (Chiralpak IA, *n*-hexane/*i*-propanol = 50/50, flow rate 1.0 mL/min, λ = 220 nm), t_major_ = 26.0 min, t_minor_ = 12.5 min, *de* = 97%.

#### 3.3.7. Ni(II)-(*S*)-BPB/(*2S*,*3R*)-2-Amino-4,4'-dicyano-3-(3-bromophenyl) Butyric Acid Schiff Base Complex (**7g**)

Yield = 38%, m.p. 208–210 °C. 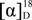 = +1882 (ca. 0.2 g/100 mL, CHCl_3_). ^1^H-NMR (CDCl_3_) δ 8.28 (d, *J* = 8.7 Hz, 1H), 8.00 (d, *J* = 7.4 Hz, 2H), 7.80 (d, *J* = 8.0 Hz, 1H), 7.75–7.60 (m, 3H), 7.49 (t, *J* = 7.8 Hz, 2H), 7.39 (d, *J* = 6.7 Hz, 1H), 7.31 (t, *J* = 7.6 Hz, 2H), 7.24–7.08 (m, 4H), 6.70 (d, *J* = 4.0 Hz, 2H), 5.14 (d, *J* = 11.9 Hz, 1H), 4.55 (d, *J* = 3.7 Hz, 1H), 4.20–4.09 (m, 1H), 3.42 (d, *J* = 12.6 Hz, 1H), 3.25 (ddd, *J* = 15.7, 10.7, 5.6 Hz, 2H), 3.03 (dd, *J* = 10.0, 5.6 Hz, 1H), 2.25 (dt, *J* = 16.4, 8.4 Hz, 1H), 2.15–1.91 (m, 4H). ^13^C-NMR (CDCl_3_) δ 180.2, 176.3, 173.3, 160.9, 143.2, 133.9, 133.4, 133.3, 133.2, 131.4, 130.8, 130.5, 129.4, 128.9, 128.8, 127.4, 127.0, 125.6, 124.1, 123.2, 120.8, 115.2, 111.6, 111.3, 70.6, 69.8, 64.0, 57.6, 55.4, 48.1, 30.5, 24.7, 22.9. ESI-MS (*m*/*z*): calcd. 730.1, found 730.2 ([M+H]^+^); HRMS (*m*/*z*): calcd. C_37_H_31_BrN_5_NiO_3_ for 730.0966, found 730.0966 ([M+H]^+^). HPLC (Chiralpak IA, *n*-hexane/*i*-propanol = 50/50, flow rate 1.0 mL/min, λ = 220 nm), t_major_ = 32.0, t_minor_ = 14.0 min, *de* = 95%.

#### 3.3.8. Ni(II)-(*S*)-BPB/(*2S*,*3R*)-2-Amino-4,4'-dicyano-3-(3-methoxyphenyl) Butyric Acid Schiff Base Complex (**7h**)

Yield = 82%, m.p. 222–224 °C. 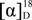 = +1678 (ca. 0.2 g/100 mL, CHCl_3_). ^1^H-NMR (CDCl_3_) δ 8.18 (d, *J* = 8.7 Hz, 1H), 7.93 (d, *J* = 7.5 Hz, 2H), 7.59 (dd, *J* = 13.8, 7.2 Hz, 3H), 7.44 (t, *J* = 7.9 Hz, 1H), 7.32 (d, *J* = 6.9 Hz, 1H), 7.23 (t, *J* = 7.5 Hz, 2H), 7.07 (dd, *J* = 13.9, 6.8 Hz, 4H), 6.75 (d, *J* = 11.2 Hz, 2H), 6.62 (d, *J* = 4.0 Hz, 2H), 5.09 (d, *J* = 12.0 Hz, 1H), 4.47 (d, *J* = 3.4 Hz, 1H), 4.04 (dd, *J* = 15.5, 9.7 Hz, 1H), 3.71 (s, 3H), 3.34 (d, *J* = 12.6 Hz, 1H), 3.16 (dd, *J* = 16.1, 6.4 Hz, 2H), 2.89 (q, *J* = 10.2 Hz, 1H), 2.26–2.06 (m, 1H), 2.01 (dd, *J* = 13.8, 7.1 Hz, 1H), 1.87 (dd, *J* = 18.6, 6.4 Hz, 2H), 1.49 (dd, *J* = 14.2, 9.1 Hz, 1H). ^13^C-NMR (CDCl_3_) δ 180.1, 176.2, 173.3, 160.6, 143.3, 133.9, 133.8, 133.4, 133.3, 133.2, 131.4, 130.9, 130.9, 130.5, 129.4, 128.9, 128.8, 127.5, 127.0, 125.5, 123.2, 120.7, 115.7, 111.5, 111.2, 70.5, 69.6, 63.9, 57.4, 55.3, 48.6, 30.6, 24.6, 23.0. ESI-MS (*m*/*z*): calcd. 682.2, found 682.3 ([M+H]^+^); HRMS (*m*/*z*): calcd. C_38_H_34_N_5_NiO_4_ for 682.1964, found 682.1959 ([M+H]^+^). HPLC (Chiralpak IA, *n*-hexane/*i*-propanol = 50/50, flow rate 1.0 mL/min, λ = 220 nm), t_major_ = 36.2, t_minor_ = 16.5 min, *de* > 99%.

#### 3.3.9. Ni(II)-(*S*)-BPB/(*2S*,*3R*)-2-Amino-4,4'-dicyano-3-(2-naphthyl) Butyric Acid Schiff Base Complex (**7i**)

Obtained as a red solid, yield = 80%, m.p. 187–189 °C. 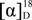 = +1582 (ca. 0.2 g/100 mL, CHCl_3_). ^1^H-NMR (CDCl_3_) δ 8.19 (d, *J* = 8.7 Hz, 1H), 8.08 (d, *J* = 8.4 Hz, 1H), 7.97 (d, *J* = 7.5 Hz, 3H), 7.93 (d, *J* = 7.7 Hz, 1H), 7.81 (s, 1H), 7.70 (d, *J* = 7.8 Hz, 3H), 7.60 (p, *J* = 6.7 Hz, 2H), 7.41 (d, *J* = 7.0 Hz, 1H), 7.32 (d, *J* = 7.0 Hz, 1H), 7.26 (t, *J* = 7.6 Hz, 3H), 7.20–7.09 (m, 2H), 6.70 (d, *J* = 14.1 Hz, 2H), 5.34 (d, *J* = 12.0 Hz, 1H), 4.62 (d, *J* = 3.1 Hz, 1H), 4.12 (d, *J* = 7.1 Hz, 1H), 4.02 (d, *J* = 12.5 Hz, 1H), 3.46 (dd, *J* = 12.0, 3.1 Hz, 1H), 3.23 (d, *J* = 12.5 Hz, 1H), 3.05 (t, *J* = 8.5 Hz, 1H), 2.64–2.48 (m, 1H), 2.04 (s, 1H), 1.74 (d, *J* = 10.0 Hz, 2H), 1.24 (dd, *J* = 16.0, 8.8 Hz, 1H). ^13^C-NMR (CDCl_3_) δ 180.1, 176.2, 173.4, 143.3, 134.1, 133.9, 133.5, 133.3, 133.2, 131.3, 130.9, 130.5, 129.8, 129.5, 128.9, 128.8, 128.5, 127.8, 127.5, 127.5, 127.3, 127.1, 125.5, 123.3, 120.7, 111.6, 111.3, 70.3, 70.0, 64.0, 57.7, 48.9, 30.0, 24.6, 22.3. ESI-MS (*m*/*z*): calcd. 702.2, found 702.3 ([M+H]^+^); HRMS (*m*/*z*): calcd. C_41_H_34_N_5_NiO_3_ for 702.2015, found 702.2022 ([M+H]^+^). HPLC (Chiralpak IA, *n*-hexane/*i*-propanol = 50/50, flow rate 1.0 mL/min, λ = 220 nm), t_major_ = 31.5 min, t_minor_ = 18.8 min, *de* = 98%.

#### 3.3.10. Ni(II)-(*S*)-BPB/(*2S*,*3R*)-2-Amino-4,4'-dicyano-3-(1-naphthyl) Butyric Acid Schiff Base Complex (**7j**)

Yield = 26%, m.p. 178–180 °C. 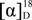 = +1614 (ca. 0.2 g/100 mL, CHCl_3_). ^1^H-NMR (CDCl_3_) δ 8.25 (d, *J* = 8.8 Hz, 1H), 8.14 (d, *J* = 8.2 Hz, 1H), 8.05 (d, *J* = 8.2 Hz, 1H), 7.89 (d, *J* = 7.5 Hz, 2H), 7.80–7.65 (m, 4H), 7.57 (d, *J* = 7.3 Hz, 1H), 7.55–7.43 (m, 3H), 7.41 (d, *J* = 7.0 Hz, 1H), 7.29–7.21 (m, 3H), 7.21–7.07 (m, 3H), 6.80–6.67 (m, 2H), 5.36 (d, *J* = 11.8 Hz, 1H), 4.74 (d, *J* = 2.6 Hz, 1H), 4.20 (dd, *J* = 11.8, 2.2 Hz, 1H), 4.12 (q, *J* = 7.1 Hz, 1H), 3.97 (d, *J* = 12.6 Hz, 1H), 3.25 (d, *J* = 12.6 Hz, 1H), 2.95 (t, *J* = 8.7 Hz, 1H), 2.50 (dt, *J* = 11.4, 5.8 Hz, 1H), 1.86 (dd, *J* = 12.9, 9.2 Hz, 1H), 1.75 (dt, *J* = 22.7, 9.6 Hz, 2H), 0.97 (dt, *J* = 14.6, 7.4 Hz, 1H). ^13^C-NMR (CDCl_3_) δ 179.5, 176.1, 173.6, 143.4, 134.4, 134.0, 133.5, 133.4, 133.1, 133.0, 131.2, 130.6, 130.4, 130.1, 129.3, 129.2, 128.8, 128.7, 127.2, 127.1, 126.9, 126.7, 126.1, 126.0, 125.1, 123.0, 122.5, 120.5, 111.6, 111.1, 71.4, 70.3, 63.7, 57.2, 43.5, 30.2, 25.7, 22.9. ESI-MS (*m*/*z*): calcd. 702.2, found 702.3 ([M+H]^+^); HRMS (*m*/*z*): calcd. C_41_H_34_N_5_NiO_3_ for 702.2015, found 702.2019 ([M+H]^+^). HPLC (Chiralpak IA, *n*-hexane/*i*-propanol = 50/50, flow rate 1.0 mL/min, λ = 220 nm), t_major_ = 55.7 min, t_minor_ = 20.4 min, *de* = 98%.

#### 3.3.11. Ni(II)-(*S*)-BPB/(*2S*,*3R*)-2-Amino-4,4'-dicyano-3-(3-hydroxyphenyl) Butyric Acid Schiff Base Complex (**7k**)

Yield = 46%, m.p. 222–224 °C. 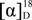 = +1775 (ca. 0.2 g/100 mL, CHCl_3_). ^1^H-NMR (DMSO) δ 9.89 (s, 1H), 8.42 (d, *J* = 7.5 Hz, 2H), 8.11 (d, *J* = 8.7 Hz, 1H), 7.78 (d, *J* = 5.3 Hz, 1H), 7.69 (s, 3H), 7.54 (d, *J* = 4.9 Hz, 1H), 7.46 (t, *J* = 7.8 Hz, 1H), 7.37 (t, *J* = 7.5 Hz, 2H), 7.13 (dd, *J* = 16.4, 6.8 Hz, 3H), 6.93–6.80 (m, 2H), 6.73 (t, *J* = 7.6 Hz, 1H), 6.63 (d, *J* = 8.3 Hz, 1H), 5.40 (d, *J* = 12.4 Hz, 1H), 4.40 (d, *J* = 3.6 Hz, 1H), 4.09 (q, *J* = 7.1 Hz, 1H), 3.91 (d, *J* = 12.2 Hz, 1H), 3.15 (dd, *J* = 12.3, 3.5 Hz, 1H), 2.96–2.84 (m, 1H), 2.56 (s, 2H), 2.23 (dd, *J* = 15.2, 8.6 Hz, 1H), 2.17–2.07 (m, 1H), 1.99 (dd, *J* = 25.2, 14.2 Hz, 2H), 1.70 (d, *J* = 6.5 Hz, 1H). ^13^C-NMR (DMSO) δ 180.0, 174.8, 171.9, 158.2, 143.2, 134.5, 134.1, 133.5, 133.1, 131.8, 131.5, 130.2, 130.1, 129.7, 128.9, 128.4, 128.1, 127.8, 127.4, 125.1, 122.8, 119.8, 116.4, 113.1, 112.1, 69.7, 69.6, 63.2, 57.7, 47.5, 30.4, 25.2, 22.6. ESI-MS (*m*/*z*): calcd. 668.2, found 668.2 ([M+H]^+^); HRMS (*m*/*z*): calcd. C_37_H_32_N_5_NiO_4_ for 668.1808, found 668.1819 ([M+H]^+^). HPLC (Chiralpak IA, *n*-hexane/*i*-propanol = 50/50, flow rate 1.0 mL/min, λ = 220 nm), t_major_ = 14.7 min, t_minor_ = 7.1 min, *de* = 98%.

#### 3.3.12. Ni(II)-(*S*)-BPB/(*2S*,*3R*)-2-Amino-4,4'-dicyano-3-(2-fluoro-4-bromophenyl) Butyric Acid Schiff Base Complex (**7l**)

Yield = 90%, m.p. 222–224 °C. 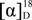 = +1624 (ca. 0.2 g/100 mL, CHCl_3_). ^1^H-NMR (CDCl_3_) δ 8.32 (d, *J* = 8.7 Hz, 1H), 8.01 (d, *J* = 7.5 Hz, 2H), 7.66 (d, *J* = 6.2 Hz, 3H), 7.60 (d, *J* = 8.6 Hz, 2H), 7.35 (d, *J* = 5.2 Hz, 1H), 7.29 (dd, *J* = 13.5, 6.0 Hz, 3H), 7.24–7.11 (m, 3H), 6.68 (s, 2H), 5.23 (d, *J* = 12.0 Hz, 1H), 4.53 (d, *J* = 3.3 Hz, 1H), 4.20–4.05 (m, 1H), 3.80 (d, *J* = 9.4 Hz, 1H), 3.45 (d, *J* = 12.6 Hz, 1H), 3.30 (t, *J* = 8.5 Hz, 1H), 2.96 (q, *J* = 10.3 Hz, 1H), 2.44–2.25 (m, 1H), 2.25–2.08 (m, 1H), 2.02–1.89 (m, 2H), 1.79–1.64 (m, 1H). ^13^C-NMR (CDCl_3_) δ 180.2, 176.3, 173.3, 160.9, 143.2, 133.9, 133.4, 133.3, 133.2, 131.4, 130.8, 130.5, 129.4, 128.9, 128.8, 127.4, 127.0, 125.6, 124.1, 123.2, 120.8, 115.2, 111.6, 111.3, 70.6, 69.8, 64.0, 57.6, 55.4, 48.1, 30.5, 24.7, 22.9. ESI-MS (*m*/*z*): calcd. 748.1, found 748.1 ([M+H]^+^); HRMS (*m*/*z*): calcd. C_37_H_30_BrFN_5_NiO_3_ for 748.0869, found 748.0881 ([M+H]^+^). HPLC (Chiralpak IA, *n*-hexane/*i*-propanol = 50/50, flow rate 1.0 mL/min, λ = 220 nm), t_major_ = 36.2 min, t_minor_ = 15.3 min, *de* > 99%.

#### 3.3.13. Ni(II)-(*S*)-BPB/(*2S*,*3R*,*4S*)-2-Amino-4-cyano-5-ethoxy-5-oxo-3-phenylpentanoic Acid Schiff Base Complex (**7m**)

Yield = 78%, m.p. 192.2–193.5 °C. 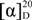 = +2323 (ca. 0.03 g/100 mL, CH_2_Cl_2_). ^1^H-NMR (CDCl_3_) δ 8.24 (d, *J* = 8.6 Hz, 1H), 7.99 (d, *J* = 7.1 Hz, 2H), 7.74–7.58 (m, 3H), 7.53 (s, 3H), 7.40 (d, *J* = 7.3 Hz, 1H), 7.35–7.26 (m, 4H), 7.22–7.08 (m, 3H), 6.70 (q, *J* = 7.7 Hz, 2H), 4.63 (s, 1H), 4.57 (d, *J* = 12.0 Hz, 1H), 4.18 (d, *J* = 12.6 Hz, 1H), 3.85 (q, *J* = 6.9 Hz, 2H), 3.39 (t, *J* = 12.9 Hz, 2H), 3.22 (t, *J* = 8.4 Hz, 1H), 2.93 (dt, *J* = 9.3, 4.6 Hz, 1H), 2.17 (dt, *J* = 16.0, 8.1 Hz, 1H), 2.02 (dd, *J* = 12.6, 6.5 Hz, 1H), 1.94 (dd, *J* = 18.3, 8.6 Hz, 1H), 1.82 (dt, *J* = 19.5, 6.8 Hz, 1H), 1.47 (ddd, *J* = 19.2, 12.4, 6.7 Hz, 1H), 0.90 (t, *J* = 6.9 Hz, 3H). ^13^C-NMR (CDCl_3_) δ 180.28, 176.58, 173.02, 164.34, 143.18, 134.17, 133.89, 133.71, 133.30, 132.85, 131.46, 130.59, 130.36, 129.32, 129.19, 129.12, 128.88, 128.80, 127.70, 127.12, 125.83, 123.20, 120.67, 114.69, 71.09, 70.50, 63.81, 62.42, 57.36, 48.45, 38.92, 30.62, 23.06, 13.48. HRMS (*m*/*z*): calcd. C_39_H_36_N_4_NaNiO_5_^+^ for 721.1931, found 721.1931 ([M+Na]^+^). HPLC (Chiralpak IA, *n*-hexane/*i*-propanol = 50/50, flow rate 1.0 mL/min, λ = 220 nm), t_major_ = 31.7 min, t_minor_ = 8.0 min, *de* = 98%.

#### 3.3.14. Ni(II)-(*S*)-BPB/(*2S*,*3R*,*4S*)-2-Amino-3-(2-bromophenyl)-4-cyano-5-ethoxy-5-oxopentanoic Acid Schiff Base Complex (**7n**)

Yield = 88%, m.p. 191.3–192.1 °C. 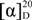 = +2120 (ca. 0.03 g/100 mL, CH_2_Cl_2_). ^1^H-NMR (CDCl_3_) δ 8.44 (d, *J* = 8.7 Hz, 1H), 7.96 (d, *J* = 7.3 Hz, 2H), 7.88 (d, *J* = 7.8 Hz, 1H), 7.69–7.61 (m, 3H), 7.58–7.53 (m, 1H), 7.53–7.44 (m, 2H), 7.43–7.36 (m, 1H), 7.29 (dd, *J* = 10.5, 4.9 Hz, 3H), 7.16 (dt, *J* = 13.7, 5.3 Hz, 2H), 6.73–6.63 (m, 2H), 4.62 (d, *J* = 3.0 Hz, 1H), 4.49 (d, *J* = 12.2 Hz, 1H), 4.17 (d, *J* = 12.6 Hz, 1H), 4.06 (dd, *J* = 12.2, 3.0 Hz, 1H), 3.89 (qd, *J* = 7.1, 2.3 Hz, 2H), 3.43 (d, *J* = 12.6 Hz, 1H), 3.25 (t, *J* = 8.6 Hz, 1H), 2.85-2.75 (m, 1H), 2.19 (ddd, *J* = 19.3, 13.1, 7.3 Hz, 2H), 1.92 (dt, *J* = 11.2, 8.1 Hz, 1H), 1.70 (dt, *J* = 13.6, 7.5 Hz, 1H), 1.46 (dt, *J* = 18.8, 6.4 Hz, 1H), 0.95 (t, *J* = 7.1 Hz, 3H). ^13^C-NMR (CDCl_3_) δ 180.14, 176.38, 174.21, 163.71, 143.31, 134.68, 134.06, 133.87, 133.70, 133.24, 133.05, 131.42, 130.80, 130.31, 130.03, 129.76, 128.87, 128.79, 128.55, 128.06, 127.12, 127.05, 125.84, 122.92, 120.52, 113.84, 71.89, 70.74, 63.75, 62.67, 57.14, 46.61, 39.74, 30.89, 23.02, 13.50, 0.01. HRMS (*m*/*z*): calcd. C_39_H_35_BrN_4_NaNiO_5_^+^for 799.1037, found 799.1034 ([M+Na]^+^). HPLC (Chiralpak IA, *n*-hexane/*i*-propanol = 50/50, flow rate 1.0 mL/min, λ = 220 nm), t_major_ =42.8 min, t_minor_ =10.6 min, *de* > 99%.

#### 3.3.15. Ni(II)-(*S*)-BPB/(*2S*,*3R*,*4S*)-2-Amino-(3-chlorophenyl)-4-cyano-5-ethoxy-3-5-oxopentanoic Acid Schiff Base Complex (**7o**)

Yield = 89%, m.p. 191.2–193.4 °C. 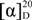 = +2376 (ca. 0.03 g/100 mL, CH_2_Cl_2_). ^1^H-NMR (CDCl_3_) δ 8.27 (d, *J* = 8.7 Hz, 1H), 8.00 (d, *J* = 7.4 Hz, 2H), 7.70 (dd, *J* = 11.3, 4.9 Hz, 1H), 7.67–7.59 (m, 2H), 7.53 (d, *J* = 8.2 Hz, 1H), 7.46 (t, *J* = 7.8 Hz, 1H), 7.42–7.34 (m, 2H), 7.31 (t, *J* = 7.6 Hz, 2H), 7.21–7.13 (m, 3H), 7.11 (d, *J* = 7.2 Hz, 1H), 6.76–6.64 (m, 2H), 4.62 (d, *J* = 3.7 Hz, 1H), 4.53 (d, *J* = 12.2 Hz, 1H), 4.19 (d, *J* = 12.6 Hz, 1H), 3.89 (q, *J* = 7.1 Hz, 2H), 3.42 (d, *J* = 12.6 Hz, 1H), 3.33 (dd, *J* = 12.2, 3.7 Hz, 1H), 3.26 (dd, *J* = 9.3, 7.7 Hz, 1H), 3.02 (dd, *J* = 10.6, 5.8 Hz, 1H), 2.23 (td, *J* = 17.0, 7.6 Hz, 1H), 2.09 (dd, *J* = 13.4, 7.4 Hz, 1H), 1.99 (dd, *J* = 10.8, 6.3 Hz, 1H), 1.93 (dd, *J* = 14.0, 7.2 Hz, 1H), 1.62 (d, *J* = 12.7 Hz, 1H), 0.96 (t, *J* = 7.1 Hz, 3H). ^13^C-NMR () δ 180.29, 176.27, 173.26, 164.17, 143.30, 136.44, 135.58, 133.91, 133.62, 133.34, 133.04, 131.45, 130.68, 130.43, 129.42, 129.26, 128.92, 128.83, 127.58, 127.06, 125.66, 123.25, 120.68, 114.35, 70.79, 70.54, 63.88, 62.61, 57.57, 48.07, 38.81, 30.76, 23.03, 13.51. HRMS (*m*/*z*): calcd. C_39_H_35_ClN_4_NaNiO_5_^+^for 755.1542, found 755.1541 ([M+Na]^+^). HPLC (Chiralpak IA, *n*-hexane/*i*-propanol = 50/50, flow rate 1.0 mL/min, λ = 220 nm), t_major_ = 26.6 min, t_minor_ = 8.1 min, *de* = 97%.

#### 3.3.16. Ni(II)-(*S*)-BPB/(*2S*,*3R*,*4S*)-2-Amino-4-cyano-5-ethoxy-3-(4-fluorophenyl)-5-oxopentanoic Acid Schiff Base Complex (**7p**)

Yield = 75%, m.p. 192.3–193.5 °C. 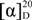 = +2250 (ca. 0.03 g/100 mL, CH_2_Cl_2_). ^1^H-NMR (CDCl_3_) δ 8.25 (d, *J* = 8.7 Hz, 1H), 8.00 (d, *J* = 7.5 Hz, 2H), 7.69 (t, *J* = 7.0 Hz, 1H), 7.67–7.59 (m, 2H), 7.39 (d, *J* = 7.3 Hz, 1H), 7.30 (dd, *J =* 14.3, 6.6 Hz, 4H), 7.22(d, *J* = 8.5, 2H), 7.16 (t, *J* = 7.3 Hz, 3H), 6.70 (q, *J* = 8.2 Hz, 2H), 4.62 (d, *J* = 3.4 Hz, 1H), 4.52 (d, *J* = 12.2 Hz, 1H), 4.19 (d, *J* = 12.6 Hz, 1H), 3.88 (q, *J* = 7.1 Hz, 2H), 3.41 (d, *J* = 12.6 Hz, 1H), 3.36 (dd, *J* = 12.2, 3.5 Hz, 1H), 3.29–3.22 (m, 1H), 2.99 (dd, *J* = 10.1, 5.7 Hz, 1H), 2.25 (td, *J* = 16.7, 7.6 Hz, 1H), 2.09 (dt, *J* = 16.1, 8.6 Hz, 1H), 1.96 (dt, *J* = 14.1, 9.6 Hz, 2H), 1.66–1.55 (m, 1H), 0.95 (t, *J* = 7.1 Hz, 3H). ^13^C-NMR (CDCl_3_) δ 180.32, 176.43, 173.22, 164.62, 164.23, 162.15, 143.22, 133.90, 133.68, 133.33, 132.97, 131.43, 130.65, 130.44, 130.04, 130.00, 129.23, 128.93, 128.83, 127.58, 127.09, 125.70, 123.22, 120.70, 116.40, 116.19, 114.44, 99.99, 70.99, 70.48, 63.91, 62.55, 57.48, 47.82, 38.99, 30.68, 22.94, 13.55. HRMS (*m*/*z*): calcd. C_39_H_35_FN_4_NaNiO_5_^+^for 739.1837, found 739.1837 ([M+Na]^+^). HPLC (Chiralpak IA, *n*-hexane/*i*-propanol = 50/50, flow rate 1.0 mL/min, λ = 220 nm), t_major_ = 53.2 min, t_minor_ = 6.9 min, *de* = 96%.

#### 3.3.17. Ni(II)-(*S*)-BPB/(*2S*,*3R*,*4S*)-2-Amino-4-cyano-3-(3,4-dichlorophenyl)-5-ethoxy-5-oxopentanoic Acid Schiff Base Complex (**7q**)

Yield = 82%, m.p. 192.7–194.7 °C. 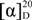 = +2353 (ca. 0.03 g/100 mL, CH_2_Cl_2_). ^1^H-NMR (CDCl_3_) δ 8.27 (d, *J* = 8.7 Hz, 1H), 8.02 (d, *J* = 7.6 Hz, 2H), 7.70 (t, *J* = 7.1 Hz, 1H), 7.63 (dd, *J* = 13.5, 8.8 Hz, 3H), 7.44 (s, 1H), 7.38 (d, *J* = 7.4 Hz, 1H), 7.31 (t, *J* = 7.6 Hz, 2H), 7.20–7.11 (m, 3H), 7.08 (d, *J* = 7.6 Hz, 1H), 6.78–6.64 (m, 2H), 4.60 (d, *J* = 3.6 Hz, 1H), 4.51 (d, *J* = 12.1 Hz, 1H), 4.19 (d, *J* = 12.5 Hz, 1H), 3.94 (q, *J* = 7.1 Hz, 2H), 3.41 (d, *J* = 12.6 Hz, 1H), 3.35–3.25 (m, 2H), 3.00 (dt, *J* = 10.2, 5.2 Hz, 1H), 2.30 (dt, *J* = 16.8, 7.7 Hz, 1H), 2.09 (dd, *J* = 13.3, 6.0 Hz, 1H), 2.02 (dd, *J* = 11.8, 7.4 Hz, 1H), 1.95 (dd, *J* = 13.4, 6.3 Hz, 1H), 1.67 (dt, *J* = 13.0, 6.4 Hz, 1H), 1.02 (t, *J* = 7.1 Hz, 3H). ^13^C-NMR (CDCl_3_) δ 180.36, 176.15, 173.45, 164.02, 143.31, 134.66, 133.94, 133.80, 133.56, 133.37, 133.17, 131.42, 131.08, 130.75, 130.50, 129.30, 128.96, 128.86, 127.48, 127.04, 125.53, 123.27, 120.75, 114.12, 70.67, 70.54, 64.03, 62.82, 57.78, 47.57, 38.59, 30.75, 22.83, 13.59. HRMS (*m*/*z*): calcd. C_39_H_34_Cl_2_N_4_NaNiO_5_^+^for 789.1152, found 789.1151 ([M+Na]^+^). HPLC (Chiralpak IA, *n*-hexane/*i*-propanol = 50/50, flow rate 1.0 mL/min, λ = 220 nm), t_major_ = 44.5 min, t_minor_ = 7.6 min, *de* = 98%.

#### 3.3.18. Ni(II)-(*S*)-BPB/(*2S*,*3R*,*4S*)-2-Amino-4-cyano-3-(2,4-dichlorophenyl)-5-ethoxy-5-oxopentanoic Acid Schiff Base Complex (**7r**)

Yield = 96%, m.p. 192.6–194.5 °C. 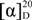 = +1960 (ca. 0.03 g/100 mL, CH_2_Cl_2_). ^1^H-NMR (CDCl_3_) δ 8.42 (d, J = 8.8 Hz, 1H), 7.99 (d, J = 7.6 Hz, 2H), 7.71 (s, 1H), 7.69–7.59 (m, 3H), 7.44 (s, 2H), 7.41 (d, J = 3.5 Hz, 1H), 7.30 (dd, J = 13.9, 6.7 Hz, 3H), 7.19–7.11 (m, 2H), 6.72–6.63 (m, 2H), 4.60 (d, J = 3.1 Hz, 1H), 4.48 (d, J = 12.2 Hz, 1H), 4.18 (d, J = 12.6 Hz, 1H), 4.05 (dd, J = 12.2, 3.1 Hz, 1H), 3.99–3.88 (m, 2H), 3.43 (d, J = 12.6 Hz, 1H), 3.34–3.22 (m, 1H), 2.90 (dt, J = 11.0, 5.6 Hz, 1H), 2.33 (dt, J = 16.8, 7.7 Hz, 1H), 2.16 (td, J = 13.6, 7.7 Hz, 1H), 2.00 (dt, J = 10.9, 7.6 Hz, 1H), 1.88 (dt, J = 14.2, 7.4 Hz, 1H), 1.69–1.53 (m, 1H), 1.01 (t, J = 7.1 Hz, 3H). ^13^C-NMR (CDCl_3_) δ 180.23, 176.30, 174.38, 163.63, 143.32, 137.69, 135.70, 134.05, 133.59, 133.31, 133.17, 131.56, 131.39, 130.83, 130.43, 130.19, 130.14, 129.65, 128.91, 128.83, 128.28, 127.08, 125.58, 122.92, 120.58, 113.73, 71.39, 70.71, 63.95, 62.85, 57.45, 43.63, 39.21, 30.98, 22.89, 13.56. HRMS (m/z): calcd. C_39_H_34_Cl_2_N_4_NaNiO_5_^+^for 789.1152, found 789.1151 ([M+Na]^+^). HPLC (Chiralpak IA, n-hexane/i-propanol = 50/50, flow rate 1.0 mL/min, λ = 220 nm), t_major_ = 77.3, t_minor_ = 8.8 min, de = 98%.

#### 3.3.19. Ni(II)-(*S*)-BPB/(*2S*,*3R*,*4S*)-2-Amino-4-cyano-5-ethoxy-5-oxo-3-(p-tolyl)pentanoic Acid Schiff Base Complex (**7s**)

Yield = 76%, m.p. 182.3–183.7 °C. 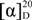 = +2570 (ca. 0.03 g/100 mL, CH_2_Cl_2_). ^1^H-NMR (CDCl_3_) δ 8.24 (d, *J* = 8.7 Hz, 1H), 8.00 (d, *J* = 7.3 Hz, 2H), 7.68 (dt, *J* = 9.7, 4.3 Hz, 1H), 7.61 (dd, *J* = 9.1, 5.4 Hz, 2H), 7.39 (d, *J* = 7.4 Hz, 1H), 7.31 (dd, *J* = 16.1, 8.0 Hz, 4H), 7.21–7.10 (m, 5H), 6.75–6.65 (m, 2H), 4.60 (d, *J* = 3.7 Hz, 1H), 4.54 (d, *J* = 12.2 Hz, 1H), 4.19 (d, *J* = 12.6 Hz, 1H), 3.86 (q, *J* = 7.1 Hz, 2H), 3.41 (d, *J* = 12.6 Hz, 1H), 3.33 (dd, *J* = 12.2, 3.7 Hz, 1H), 3.26–3.19 (m, 1H), 3.01–2.93 (m, 1H), 2.43 (s, 3H), 2.20 (dt, *J* = 16.7, 7.9 Hz, 1H), 2.04 (dt, *J* = 13.1, 6.8 Hz, 1H), 1.95 (dd, *J* = 11.1, 7.2 Hz, 1H), 1.83 (tt, *J* = 15.3, 7.6 Hz, 1H), 1.49 (tt, *J* = 12.8, 6.4 Hz, 1H), 0.94 (t, *J* = 7.1 Hz, 3H). ^13^C-NMR (CDCl_3_) δ 180.19, 176.62, 172.87, 164.36, 143.12, 138.95, 133.86, 133.71, 133.31, 132.80, 131.45, 130.97, 130.55, 130.30, 129.97, 129.17, 128.87, 128.79, 127.70, 127.11, 125.83, 123.17, 120.65, 114.76, 71.12, 70.55, 63.87, 62.37, 57.40, 48.10, 38.85, 30.47, 22.78, 21.27, 13.51. HRMS (*m*/*z*): calcd. C_40_H_38_N_4_NaNiO_5_^+^for 735.2088, found 735.2089 ([M+Na]^+^). HPLC (Chiralpak IA, *n*-hexane/*i*-propanol = 50/50, flow rate 1.0 mL/min, λ = 220 nm), t_major_ = 46.3 min, t_minor_ = 7.5 min, *de* > 99%.

#### 3.3.20. Ni(II)-(*S*)-BPB/(*2S*,*3R*,*4S*)-2-Amino-4-cyano-5-ethoxy-3-(4-methoxylphenyl)-5-oxopentanoic Acid Schiff Base Complex (**7t**)

Yield = 77%, m.p. 188.5–189.4 °C. 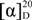 = +2376 (ca. 0.03 g/100 mL, CH_2_Cl_2_). ^1^H-NMR (CDCl_3_) δ 8.23 (d, *J* = 8.7 Hz, 1H), 8.01 (d, *J* = 7.5 Hz, 2H), 7.68 (t, *J* = 6.9 Hz, 1H), 7.65–7.58 (m, 2H), 7.38 (d, *J* = 7.4 Hz, 1H), 7.30 (t, *J* = 7.6 Hz, 2H), 7.21 (d, *J* = 8.1 Hz, 2H), 7.15 (dd, *J* = 13.2, 6.6 Hz, 3H), 7.04 (d, *J* = 8.5 Hz, 2H), 6.74–6.65 (m, 2H), 4.60 (d, *J* = 3.5 Hz, 1H), 4.52 (d, *J* = 12.2 Hz, 1H), 4.19 (d, *J* = 12.6 Hz, 1H), 3.91–3.86 (m, 2H), 3.85 (s, 3H), 3.40 (d, *J* = 12.6 Hz, 1H), 3.32 (dd, *J* = 12.2, 3.5 Hz, 1H), 3.24 (t, *J* = 8.5 Hz, 1H), 3.04–2.96 (m, 1H), 2.21 (dt, *J* = 16.3, 7.6 Hz, 1H), 2.08 (dd, *J* = 13.3, 6.2 Hz, 1H), 1.98 (dd, *J* = 11.0, 6.9 Hz, 1H), 1.90 (dd, *J* = 13.5, 6.8 Hz, 1H), 1.56–1.47 (m, 1H), 0.95 (t, *J* = 7.1 Hz, 3H). ^13^C-NMR (CDCl_3_) δ 180.28, 176.63, 172.88, 164.42, 160.34, 143.15, 133.85, 133.73, 133.37, 132.79, 131.46, 130.56, 130.32, 129.17, 128.88, 128.79, 127.67, 127.13, 125.85, 125.78, 123.18, 120.65, 114.73, 114.59, 71.20, 70.59, 63.92, 62.37, 57.55, 55.34, 47.89, 38.97, 30.62, 22.94, 13.56. HRMS (*m*/*z*): calcd. C_40_H_38_N_4_NaNiO_6_^+^for 751.2037, found 751.2037 ([M+Na]^+^). HPLC (Chiralpak IA, *n*-hexane/*i*-propanol = 50/50, flow rate 1.0 mL/min, λ = 220 nm), t_major_ = 71.2 min, t_minor_ = 8.5 min, *de* = 97%.

#### 3.3.21. Ni(II)-(*S*)-BPB/(*2S*,*3R*,*4S*)-2-Amino-4-cyano-5-ethoxy-3-(4-nitrophenyl)-5-oxopentanoic Acid Schiff Base Complex (**7u**)

Yield = 69%, m.p. 206.5–208.6 °C. 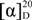 = +2163 (ca. 0.03 g/100 mL, CH_2_Cl_2_). ^1^H-NMR (CDCl_3_) δ 8.40 (d, *J* = 8.5 Hz, 2H), 8.28 (d, *J* = 8.7 Hz, 1H), 7.98 (d, *J* = 7.5 Hz, 2H), 7.72 (t, *J* = 7.1 Hz, 1H), 7.69–7.60 (m, 2H), 7.48 (d, *J* = 8.4 Hz, 2H), 7.41 (d, *J* = 7.4 Hz, 1H), 7.30 (t, *J* = 7.6 Hz, 2H), 7.21–7.11 (m, 3H), 6.75–6.67 (m, 2H), 4.67 (d, *J* = 3.5 Hz, 1H), 4.62 (d, *J* = 12.1 Hz, 1H), 4.17 (d, *J* = 12.6 Hz, 1H), 3.96–3.86 (m, 2H), 3.48 (dd, *J* = 12.1, 3.5 Hz, 1H), 3.40 (d, *J* = 12.6 Hz, 1H), 3.23 (dd, *J* = 9.7, 7.1 Hz, 1H), 2.95–2.87 (m, 1H), 2.17 (dt, *J* = 17.7, 8.9 Hz, 1H), 1.97–1.86 (m, 2H), 1.68 (dd, *J* = 17.8, 10.7 Hz, 1H), 1.55–1.46 (m, 1H), 1.00 (t, *J* = 7.1 Hz, 3H). ^13^C-NMR (CDCl_3_) δ 180.25, 176.03, 173.78, 163.86, 148.69, 143.31, 142.00, 134.01, 133.59, 133.32, 133.25, 131.36, 130.83, 130.64, 129.33, 129.00, 128.89, 127.47, 127.03, 125.46, 124.23, 123.26, 120.85, 113.96, 70.71, 70.32, 63.95, 62.93, 57.36, 47.97, 38.63, 30.64, 22.83, 13.61. HRMS (*m*/*z*): calcd. C_39_H_35_N_5_NaNiO_7_^+^for 766.1782, found 766.1782 ([M+Na]^+^). HPLC (Chiralpak IA, *n*-hexane/*i*-propanol = 50/50, flow rate 1.0 mL/min, λ = 220 nm), t_major_ = 69.0 min, t_minor_ = 8.6 min, *de* = 97%.

#### 3.3.22. Ni(II)-(*S*)-BPB/(*2S*,*3R*,*4S*)-2-Amino-3-(4-(tert-butyl)phenyl)-4-cyano-5-ethoxy-5-oxopentanoic Acid Schiff Base Complex (**7v**)

Yield = 51%, m.p. 200.8–201.7 °C. 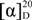 = +2260 (ca. 0.03 g/100 mL, CH_2_Cl_2_). ^1^H-NMR (CDCl_3_) δ 8.29 (d, *J* = 8.7 Hz, 1H), 7.95 (d, *J* = 7.4 Hz, 2H), 7.72–7.66 (m, 1H), 7.66–7.59 (m, 2H), 7.50 (d, *J* = 8.3 Hz, 2H), 7.40 (d, *J* = 7.4 Hz, 1H), 7.30 (t, *J* = 7.6 Hz, 2H), 7.17 (dt, *J* = 15.7, 7.6 Hz, 5H), 6.75–6.66 (m, 2H), 4.63 (d, *J* = 3.7 Hz, 1H), 4.53 (d, *J* = 12.2 Hz, 1H), 4.20 (d, *J* = 12.7 Hz, 1H), 3.86–3.76 (m, 2H), 3.49 (d, *J* = 12.7 Hz, 1H), 3.32 (dd, *J* = 12.2, 3.7 Hz, 1H), 3.17 (dd, *J* = 9.8, 7.4 Hz, 1H), 3.00 (dd, *J* = 10.3, 6.8 Hz, 1H), 2.18 (dt, *J* = 18.1, 8.3 Hz, 1H), 2.06 (dd, *J* = 11.2, 6.6 Hz, 1H), 1.89 (dd, *J* = 19.4, 8.6 Hz, 1H), 1.80 (dd, *J* = 10.3, 6.2 Hz, 1H), 1.48 (d, *J* = 8.3 Hz, 1H), 1.34 (s, 9H), 0.79 (t, *J* = 7.1 Hz, 3H). ^13^C-NMR (CDCl_3_) δ 180.08, 176.69, 173.06, 164.52, 151.93, 143.13, 133.93, 133.77, 133.17, 132.84, 131.41, 130.88, 130.56, 130.34, 129.16, 128.85, 128.80, 127.82, 127.07, 126.29, 126.25, 125.92, 123.10, 120.69, 114.80, 70.98, 70.48, 63.49, 62.22, 56.56, 48.08, 39.24, 34.76, 31.35, 30.72, 22.77, 13.35. HRMS (*m*/*z*): calcd. C_43_H_44_N_4_NaNiO_5_^+^for 777.2557, found 777.2557 ([M+Na]^+^). HPLC (Chiralpak IA, *n*-hexane/*i*-propanol = 50/50, flow rate 1.0 mL/min, λ = 220 nm), t_major_ = 58.6 min, t_minor_ = 8.7 min, *de* = 97%.

#### 3.3.23. Ni(II)-(*S*)-BPB/(*2S*,*3R*,*4S*)-2-Amino-4-cyano-5-ethoxy-3-(naphthalen-1-y)-5-oxopentanoic Acid Schiff Base Complex (**7w**)

Yield = 67%, m.p. 188.4–190.3 °C. 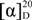 = +1793 (ca. 0.03 g/100 mL, CH_2_Cl_2_). ^1^H-NMR (CDCl_3_) δ 8.25 (d, *J* = 8.7 Hz, 1H), 8.00 (dd, *J* = 16.3, 8.0 Hz, 2H), 7.90 (d, *J* = 7.5 Hz, 2H), 7.71 (dd, *J* = 16.1, 8.1 Hz, 3H), 7.66–7.56 (m, 3H), 7.54–7.44 (m, 2H), 7.41 (d, *J* = 7.7 Hz, 1H), 7.23 (d, *J =* 7.4 Hz, 2H), 7.18–7.07 (m, 3H), 6.77 (d, *J* = 8.2 Hz, 1H), 6.71 (t, *J* = 7.5 Hz, 1H), 4.80 (s, 1H), 4.76 (d, *J* = 12.1 Hz, 1H), 4.30 (d, *J* = 10.7 Hz, 1H), 4.02 (d, *J* = 12.6 Hz, 1H), 3.80–3.65 (m, 2H), 3.25 (d, *J* = 12.5 Hz, 1H), 2.94 (t, *J* = 8.7 Hz, 1H), 2.51 (dt, *J* = 11.5, 5.9 Hz, 1H), 1.89–1.72 (m, 2H), 1.24 (dt, *J* = 13.6, 6.8 Hz, 2H), 0.94 (dt, *J* = 21.1, 7.7 Hz, 1H), 0.67 (t, *J* = 7.1 Hz, 3H). ^13^C-NMR (CDCl_3_) δ 179.70, 176.53, 173.06, 164.07, 143.34, 134.19, 133.94, 133.72, 133.47, 133.23, 133.08, 131.36, 131.22, 130.96, 130.45, 129.86, 129.42, 128.98, 128.76, 128.69, 127.37, 126.94, 126.86, 126.59, 126.02, 125.80, 125.42, 122.98, 122.94, 120.40, 114.48, 72.69, 70.32, 63.60, 62.39, 57.23, 43.09, 39.99, 30.34, 22.94, 13.28. HRMS (*m*/*z*): calcd. C_43_H_38_N_4_NaNiO_5_^+^for 771.2088, found 771.2088 ([M+Na]^+^). HPLC (Chiralpak IA, *n*-hexane/*i*-propanol = 50/50, flow rate 1.0 mL/min, λ = 220 nm), t_major_ = 31.7 min, t_minor_ = 12.3 min, *de* > 99%.

### 3.4. Procedure for the Synthesis of (2S,3R)-**8a**

In a typical procedure, 3 mol/L HCl (3.33 mL, 5.0 mmol) was added to a solution of the (*S*,2*S*,3*R*)-**7a** (1.0 mmol) dissolved in THF (13 mL). The reaction was stirred for 12 h or until the red color of the solution disappeared and was then concentrated under vacuum to half of the original volume. In the case of (*S*,2*S*,3*R*)-**7a**, the (*S*)-BPB was recovered from the aqueous portion by extracting with ethyl acetate (EA) and was washed with water. The organic layer was removed, and the aqueous portion was diluted with water (2 mL). The aqueous portion was transferred to a clean flask, and solid NaHCO_3_ (336 mg, 4.0 mmol) was carefully added with stirring to neutralize the solution, followed by Na_2_EDTA (372 mg, 1.0 mmol), and was stirred for 5 min. Additional solid NaHCO_3_ (336 mg, 4.0 mmol) was added, followed by a solution of Fmoc-OSu (337 mg,1.0 mmol) in acetonitrile (5 mL). The reaction was stirred for 24 h under nitrogen, concentrated in vacuum to half of the original volume, adjusted to pH = 3 with 10% citric acid, and extracted with EtOAc twice. Combined organic layers were washed with brine, dried with anhydrous MgSO_4_, concentrated, and purified on silica gel using a flash chromatography (petroleum ether/ethyl acetate = 1/2) to give (2*S*,3*R*)-**8a** as a white solid.

### 3.5. 2-(((9H-Fluoren-9-yl)methoxy)carbonyl)-4,4-dicyano-3-phenylbutanoic Acid (**8a**)

^1^H-NMR (CDCl_3_) δ 8.02 (s, 1H), 7.68–7.54 (m, 3H), 7.54–7.41 (m, 2H), 7.21–6.97 (m, 6H), 6.58 (s, 1H), 4.42 (s, 1H), 4.35–4.17 (m, 2H), 4.11 (s, 1H), 4.06 (s, 1H), 3.82 (s, 1H). ^13^C-NMR (DMSO) δ 172.8, 162.3, 150.4, 143.2, 140.6, 140.6, 128.9, 128.8, 127.8, 127.7, 127.2, 127.2, 127.1, 126.4, 121.3, 120.1, 120.0, 109.7, 67.8, 59.7, 46.1, 25.1, 20.7. ESI-MS (*m*/*z*): calcd. 450.2, found 450.4 ([M−H]^−^).

## 4. Conclusions

We have reported the first asymmetric three-component reaction of chiral nickel(II) glycinate, aromatic aldehydes, and an α-carbanion of two electron-withdrawing groups (malononitrile or ethyl cyanoacetate) to give a series of novel α-amino-β-substituted γ,γ-disubstituted butyric acid derivatives. We have screened a series of reaction conditions and developed a practical system to promote the asymmetric three component reaction of chiral nicke(II) glycinate. This reaction, which constructed two carbon-carbon bonds and formed two or three chiral centers, provides a convenient synthesis of functionalized chiral Fmoc-α-amino-β-substituted γ,γ-disubstituted butyric acid derivatives. The transformation performed well with electron-deficient, electron-rich, condensed ring and sterically hindered aromatic aldehydes and addorded functionalized products. To our excitement, some of them had amazingly high diastereoselectivities, but the heteroaryl substitutes were not well tolerated. The absolute configurations of the typical products were determined. Further studies will focus on mechanistic aspects, expansion of substrate ranges, and further applications of other chiral nickel(II) complexes in important carbon-carbon bond-forming reactions.

## Supplementary Materials

Supplementary materials can be accessed at: http://www.mdpi.com/1420-3049/19/1/826/s1.
