# Supplementary material for: Diastereoselective Three-Component Reactions of Chiral Nickel(II) Glycinate for Convenient Synthesis of Novel α-Amino-β-Substituted-γ,γ-Disubstituted Butyric Acids
Source: Molecules. 2014 Jan 10;19(1):826–45. doi: 10.3390/molecules19010826 (PMC6271210; doi:10.3390/molecules19010826)
Supplement: Supplementary file 1 [file molecules-19-00826-s001.pdf]

Supplementary Materials

Figure S1. ^{1}H-NMR Spectrum of Compound **7a**.

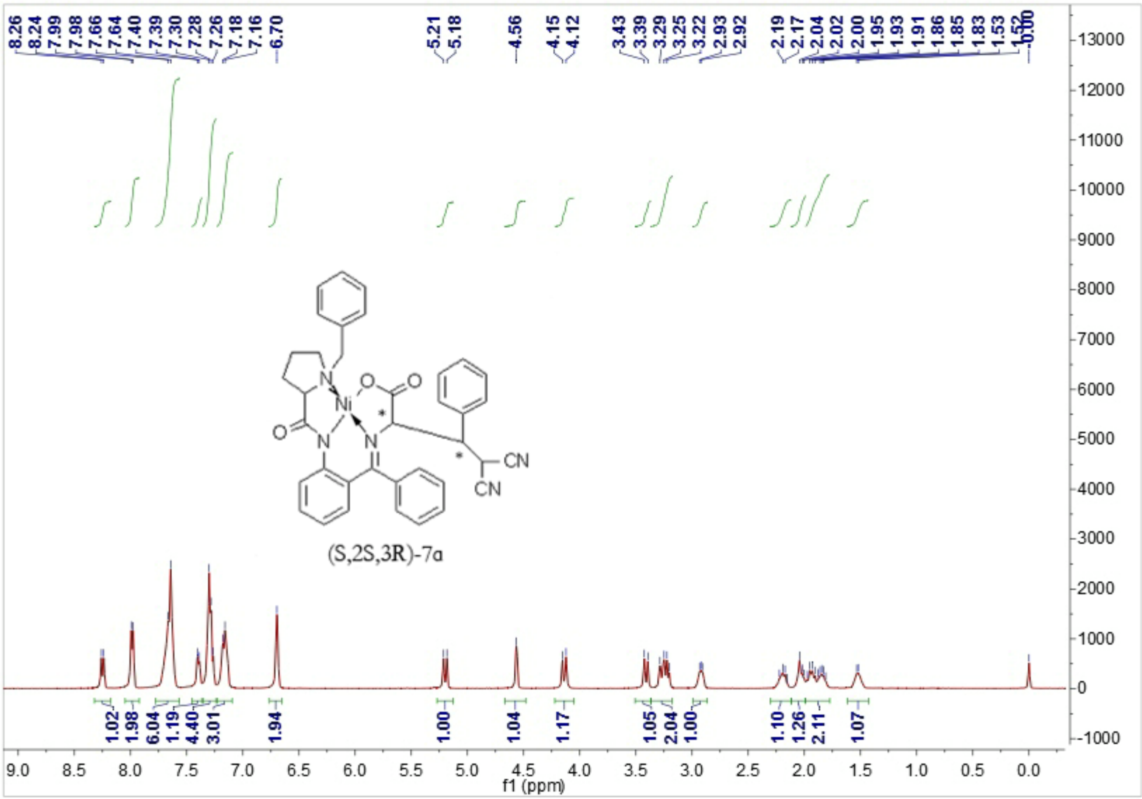

Figure S2. ^{13}C-NMR Spectrum of Compound **7a**.

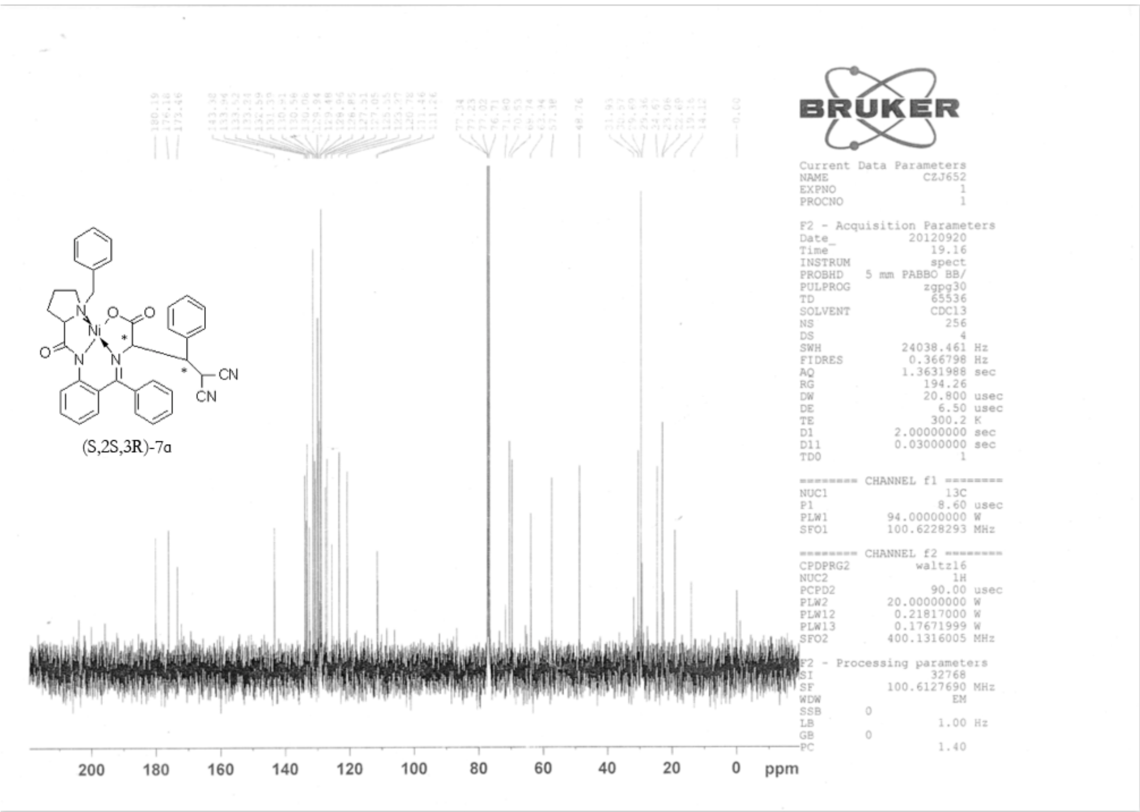

Figure S3. HPLC Spectra of Compound **7a**.

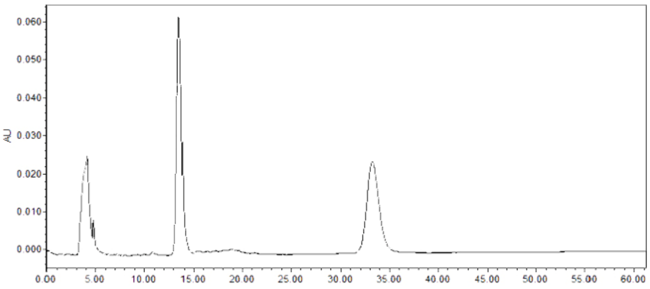

	rt(min)	Area	%Area	Type
1	13.495	5851481	50.63	bb
2	33.229	5705073	49.37	bb

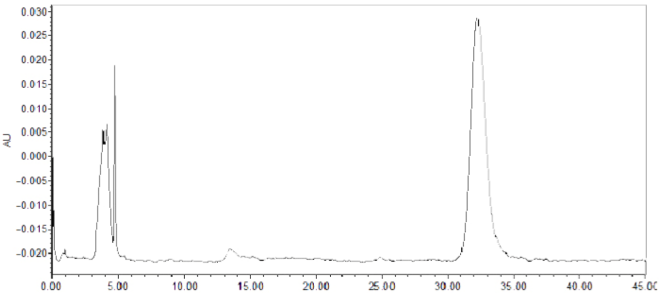

	rt(min)	Area	%Area	Type
1	13.396	148496	1.34	bb
2	32.261	10956015	98.66	bb

Figure S4. ^{1}H-NMR Spectru of Compound **7b**.

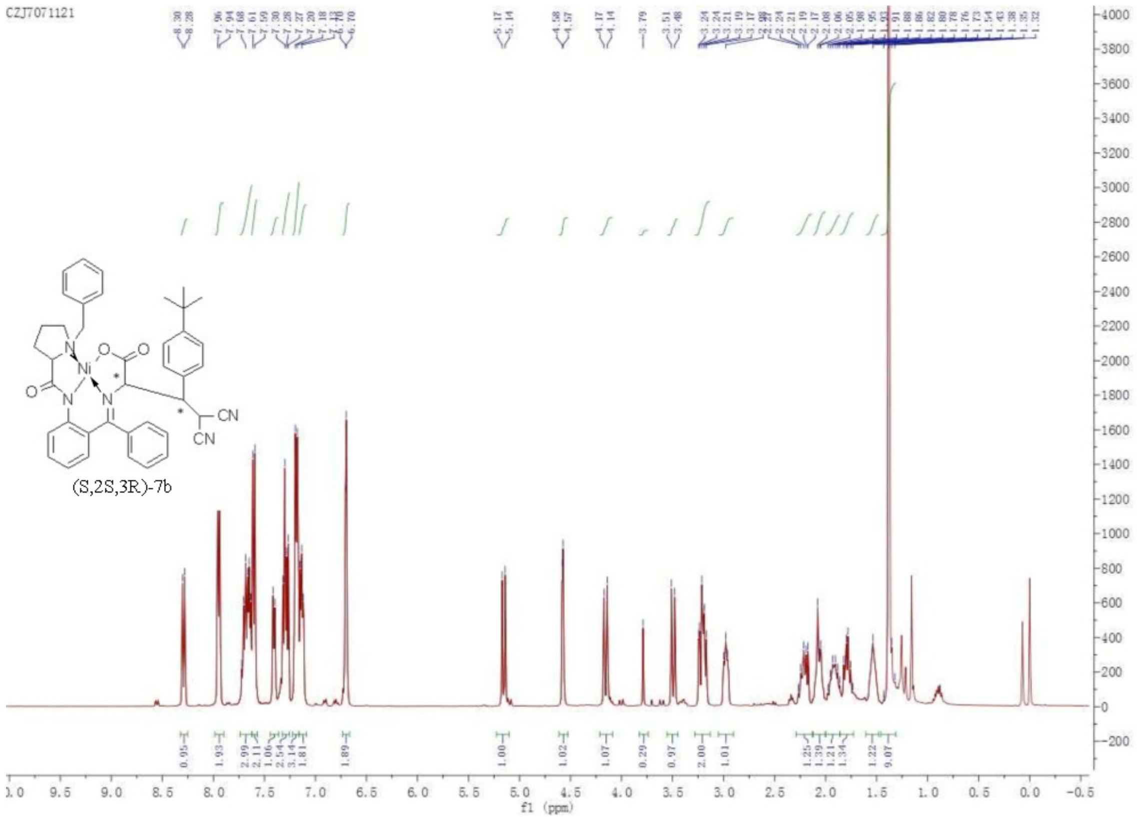

Figure S5. ^{13}C-NMR Spectrum of Compound **7b**.

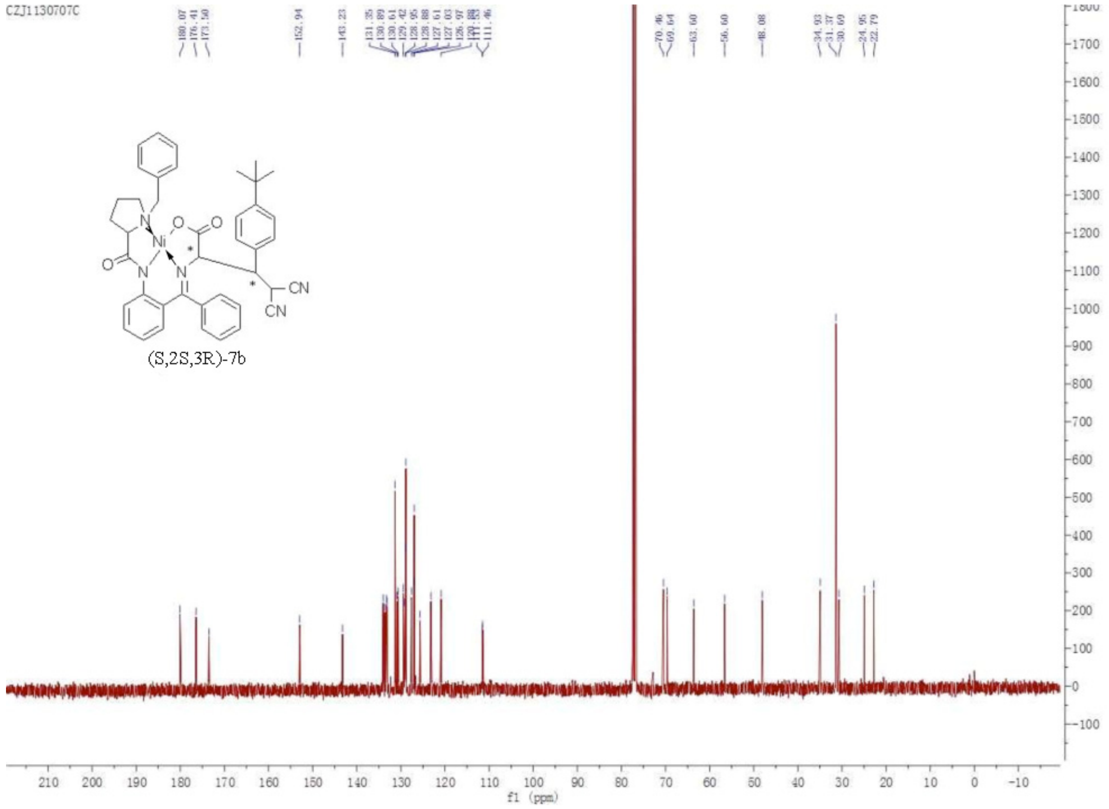

Figure S6. HPLC Spectra of Compound **7b**.

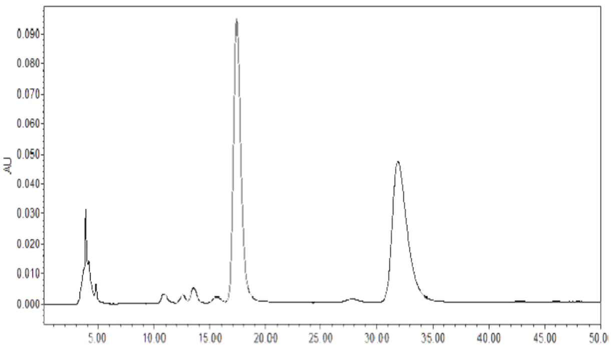

	rt(min)	Area	%Area	Type
1	17.363	4347423	50.56	bb
2	31.918	4250551	49.44	bb

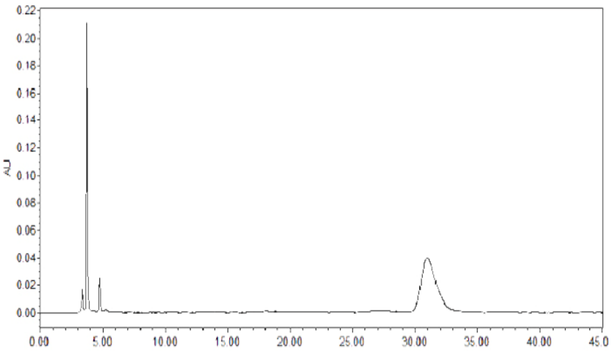

	rt(min)	Area	%Area	Type
1	30.914	3260786	100	bb

Figure S7. ¹H-NMR Spectrum of Compound **7c**.

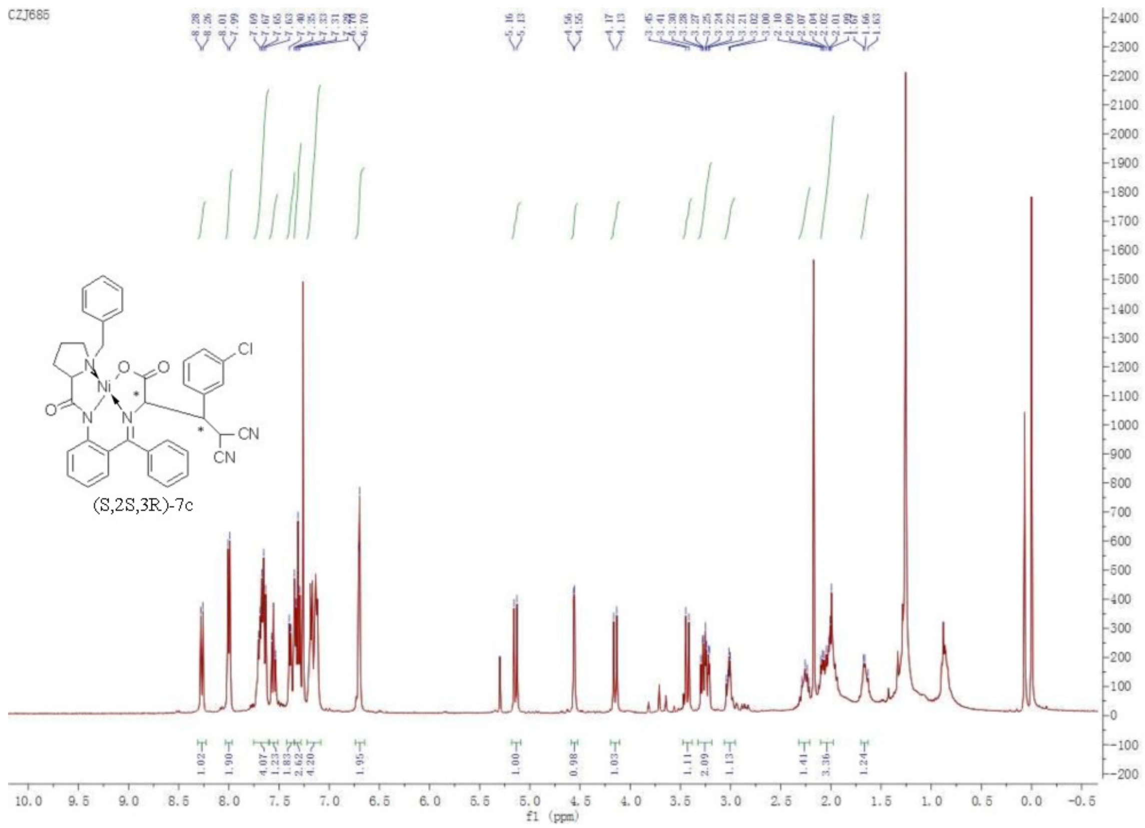

Figure S8. ¹³C-NMR Spectrum of Compound **7c**.

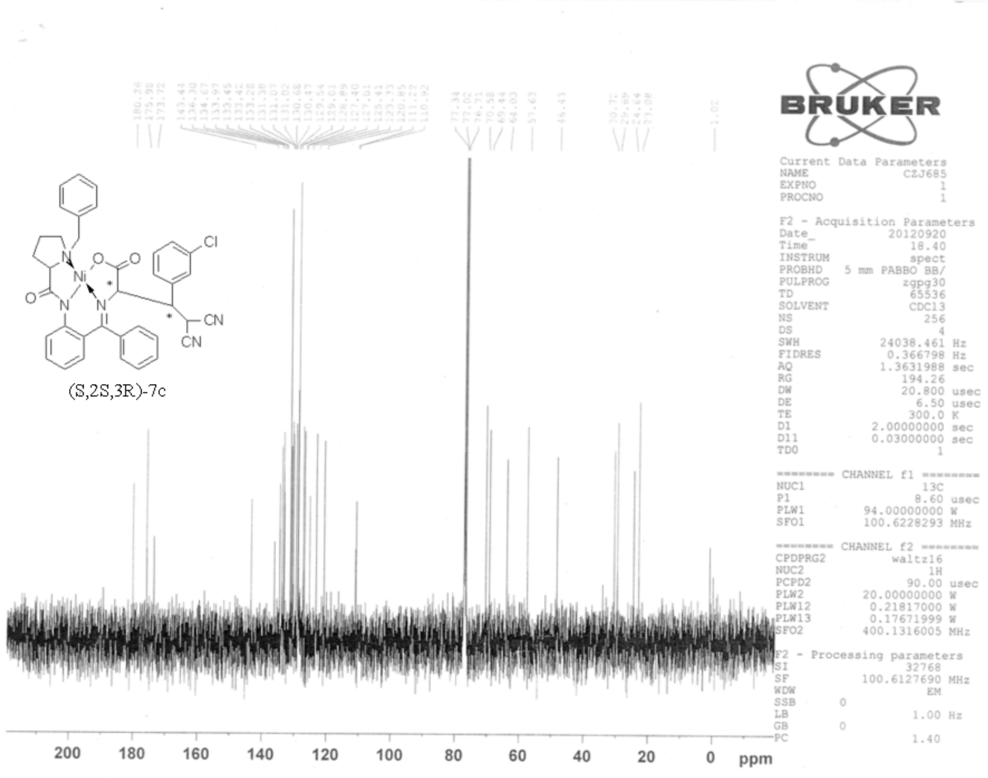

Figure S9. HPLC Spectra of Compound **7c**.

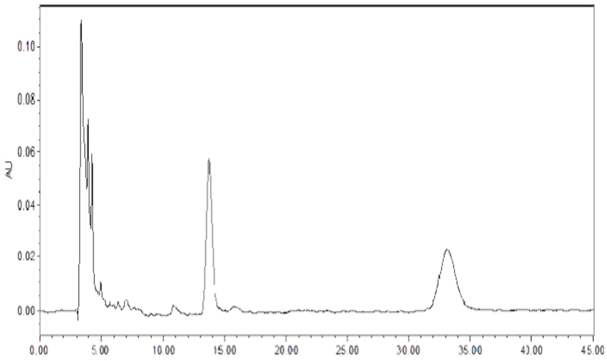

	rt(min)	Area	%Area	Type
1	13.738	1936535	49.96	bb
2	33.103	1939533	50.04	bb

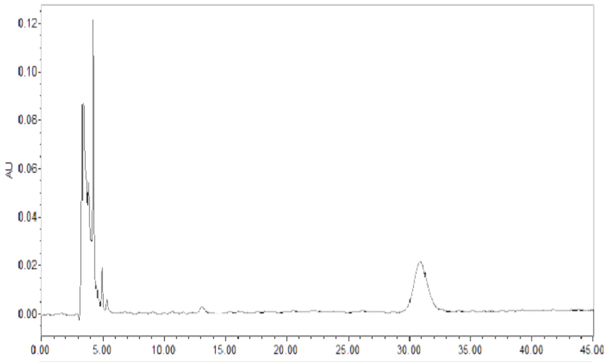

	rt(min)	Area	%Area	Type
1	13.042	13.042	4.67	bb
2	30.878	1544894	95.33	bb

Figure S10. ^{1}H-NMR Spectrum of Compound **7d**.

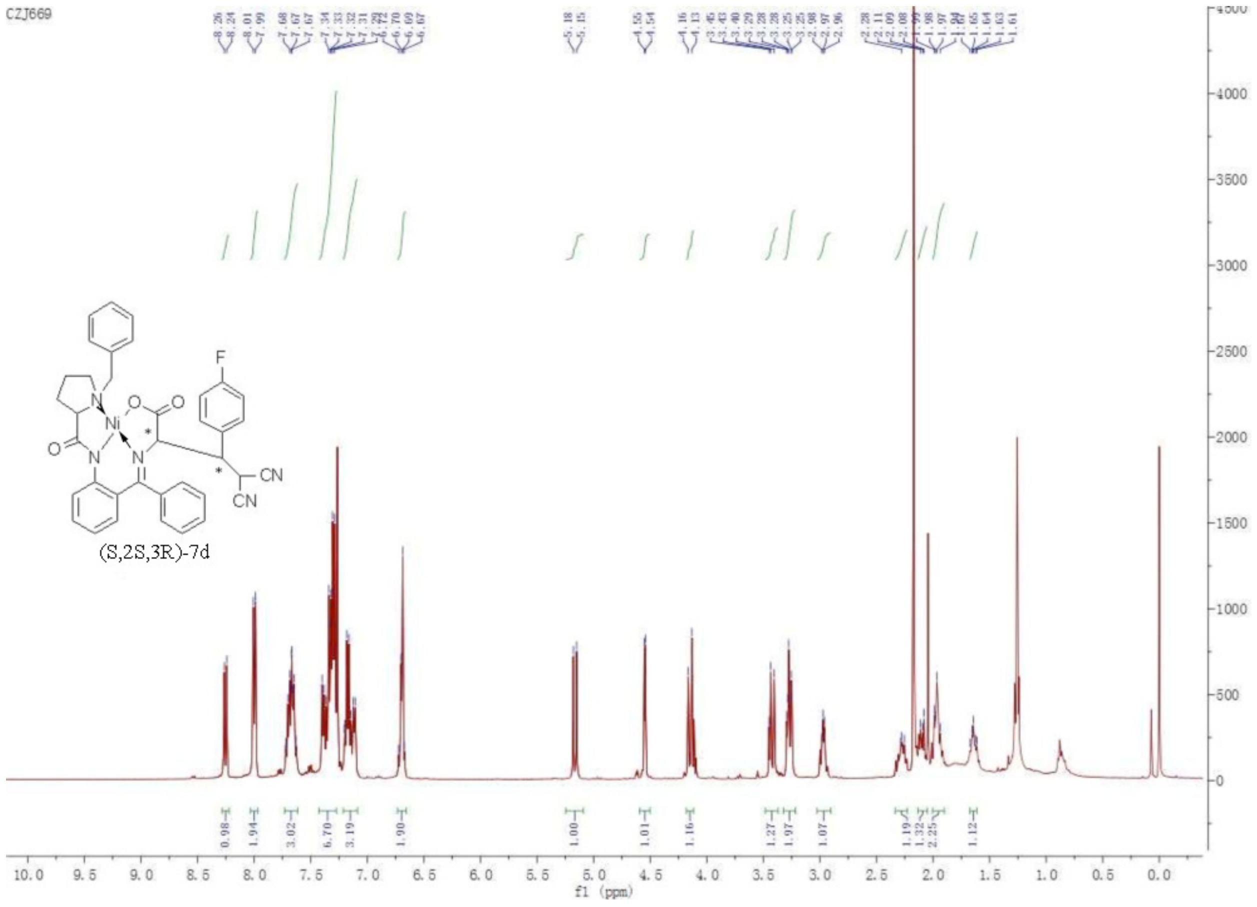

Figure S11. ^{13}C-NMR Spectrum of Compound **7d**.

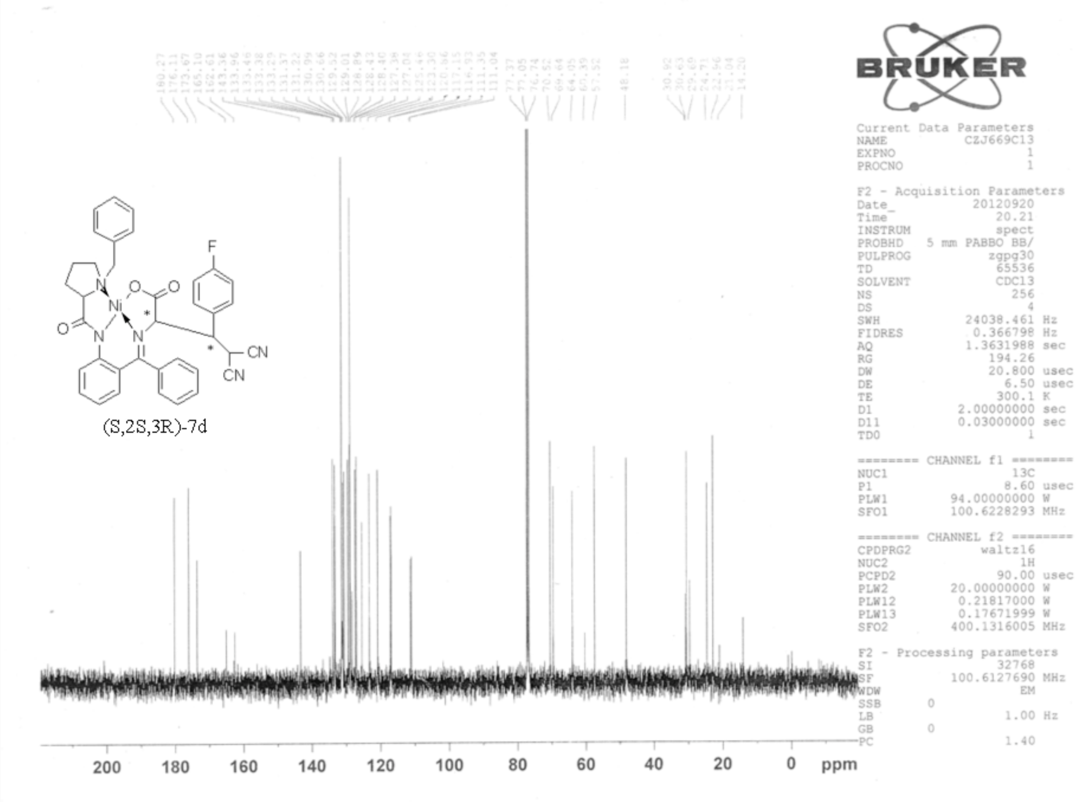

Figure S12. HPLC Spectra of Compound **7d**.

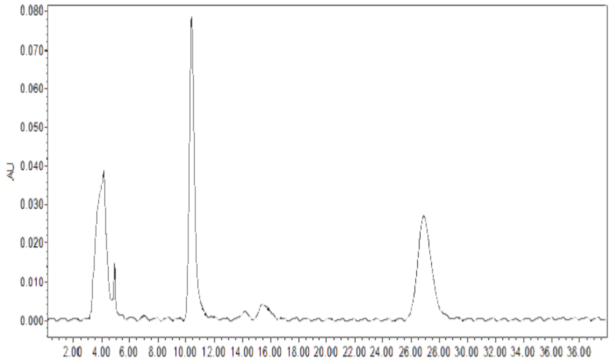

	rt(min)	Area	%Area	Type
1	10.409	5175400	51.77	bb
2	26.906	4820644	48.23	bb

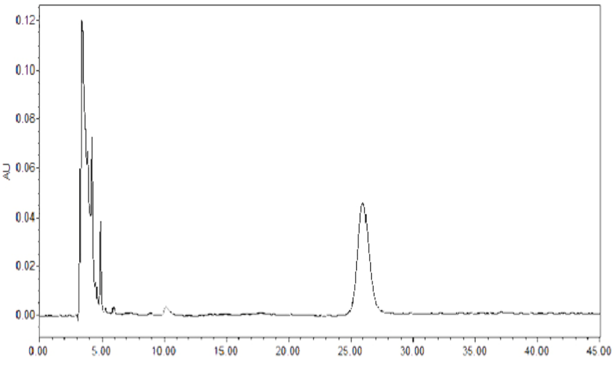

	rt(min)	Area	%Area	Type
1	10.16	101243	3.45	bb
2	25.963	2833768	96.55	bb

Figure S13. ^{1}H-NMR Spectrum of Compound **7e**.

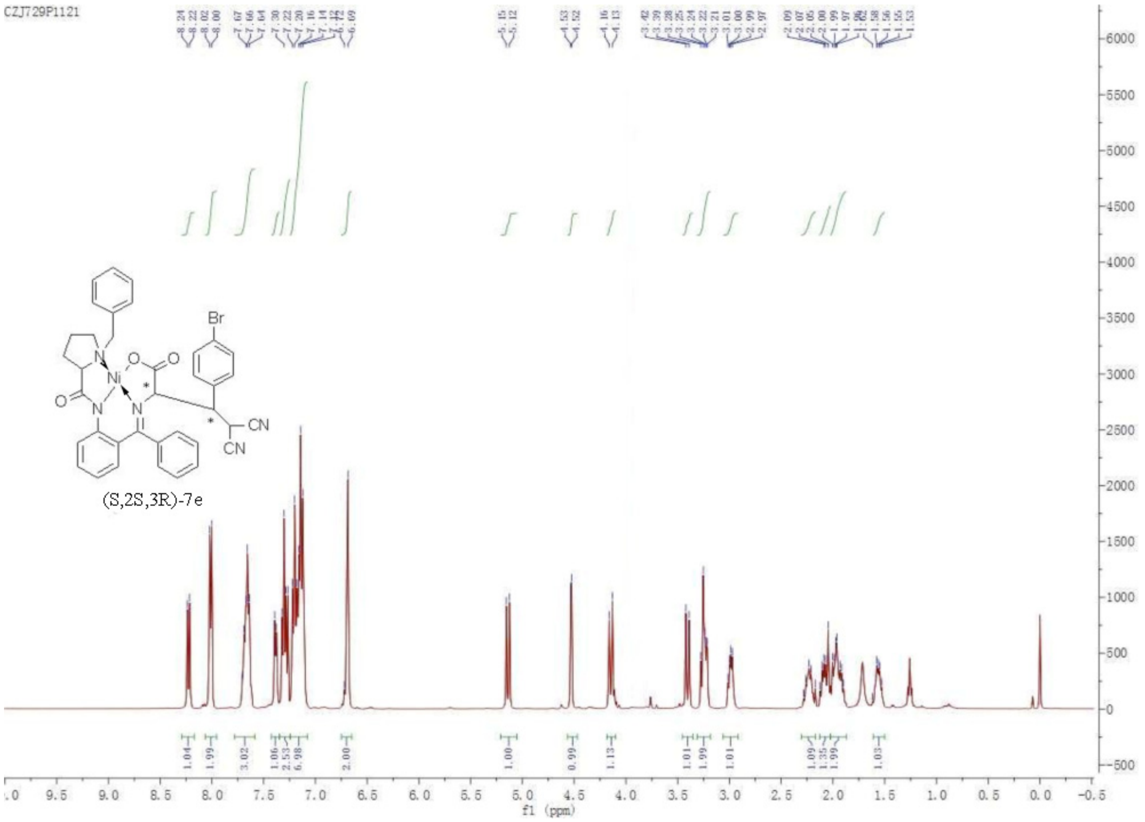

Figure S14. ^{13}C-NMR Spectrum of Compound **7e**.

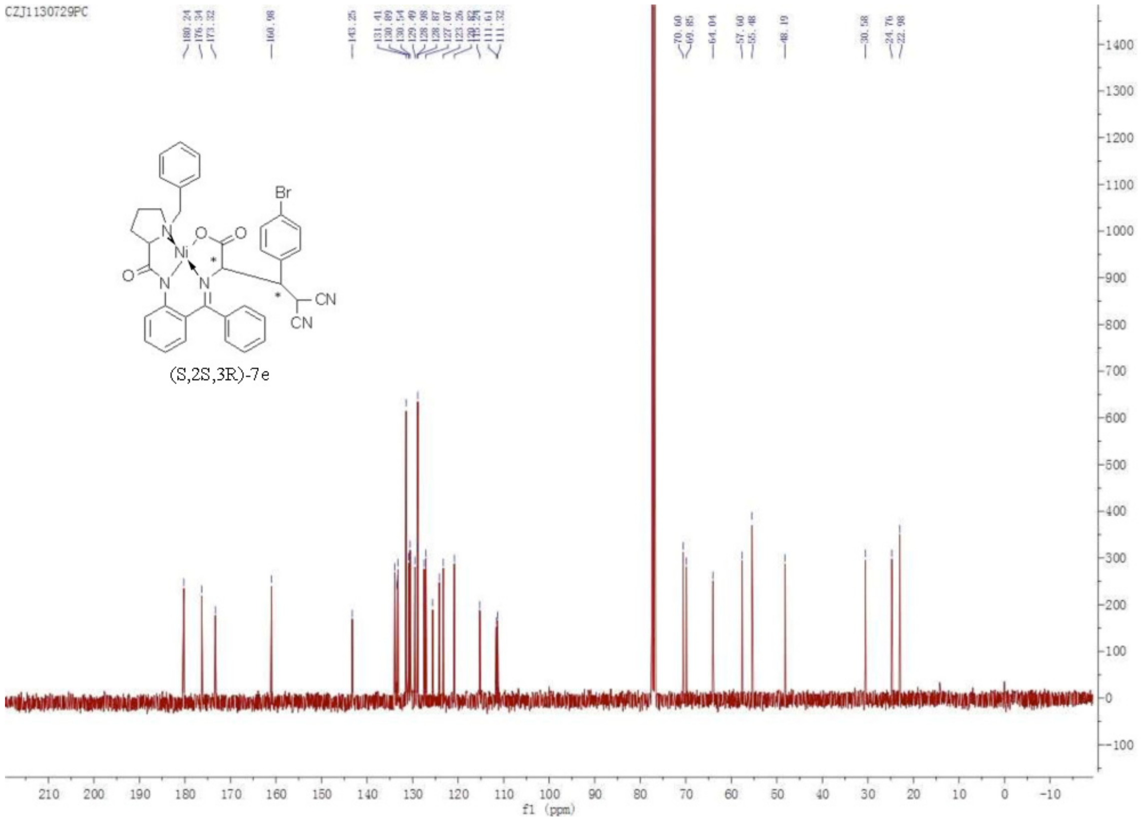

Figure S15. HPLC Spectra of Compound **7e**.

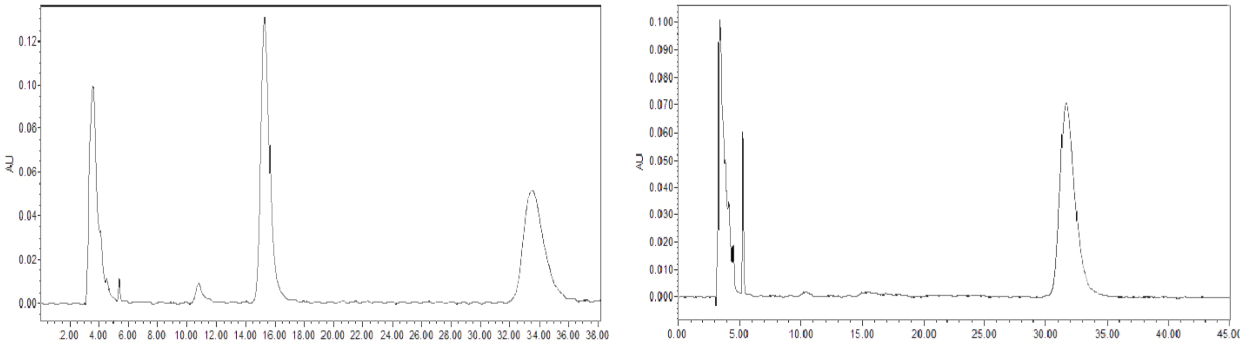

	rt(min)	Area	%Area	Type
1	15.306	6682575	52.81	bb
2	33.518	5972338	47.19	bb

	rt(min)	Area	%Area	Type
1	15.312	41961	0.72	bb
2	31.662	5759255	99.28	bb

Figure S16. ^{1}H-NMR Spectrum of Compound **7f**.

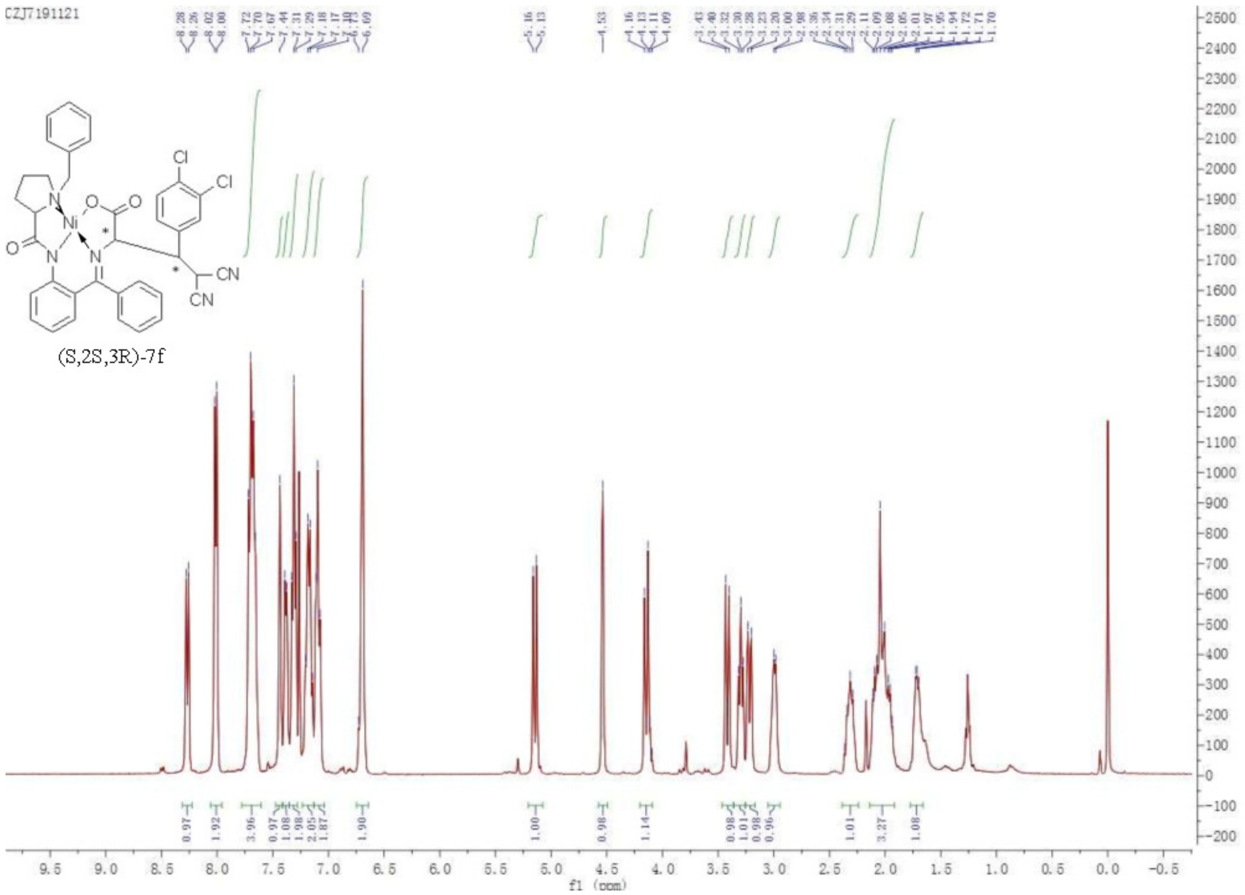

Figure S17. ^{13}C-NMR Spectrum of Compound **7f**.

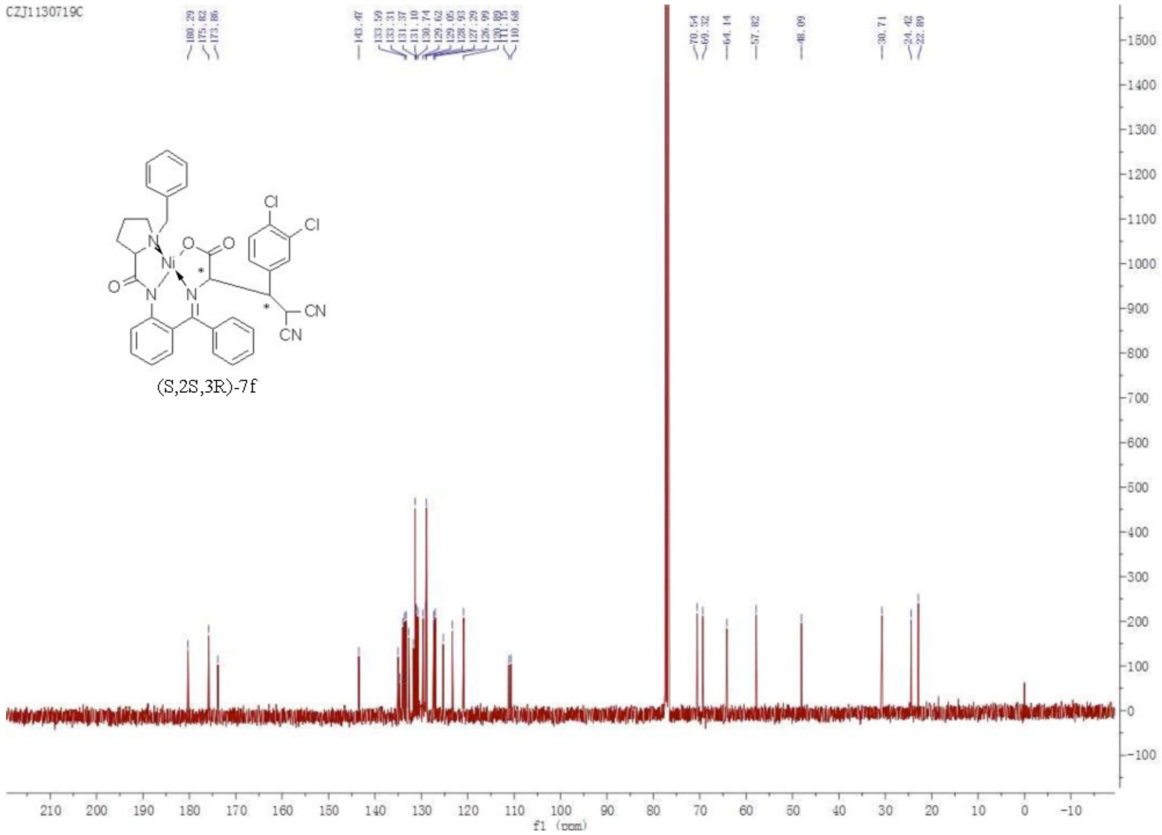

Figure S18. HPLC Spectra of Compound **7f**.

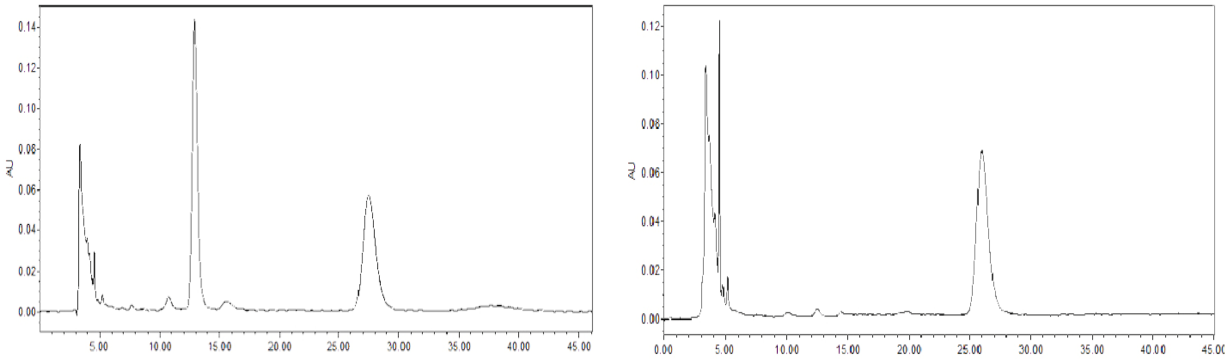

	rt(min)	Area	%Area	Type
1	12.947	4420816	50.67	bb
2	27.304	4303825	49.33	bb

	rt(min)	Area	%Area	Type
1	12.512	67860	1.49	bb
2	26.000	4498091	98.51	bb

Figure S19. ^{1}H-NMR Spectrum of Compound **7g**.

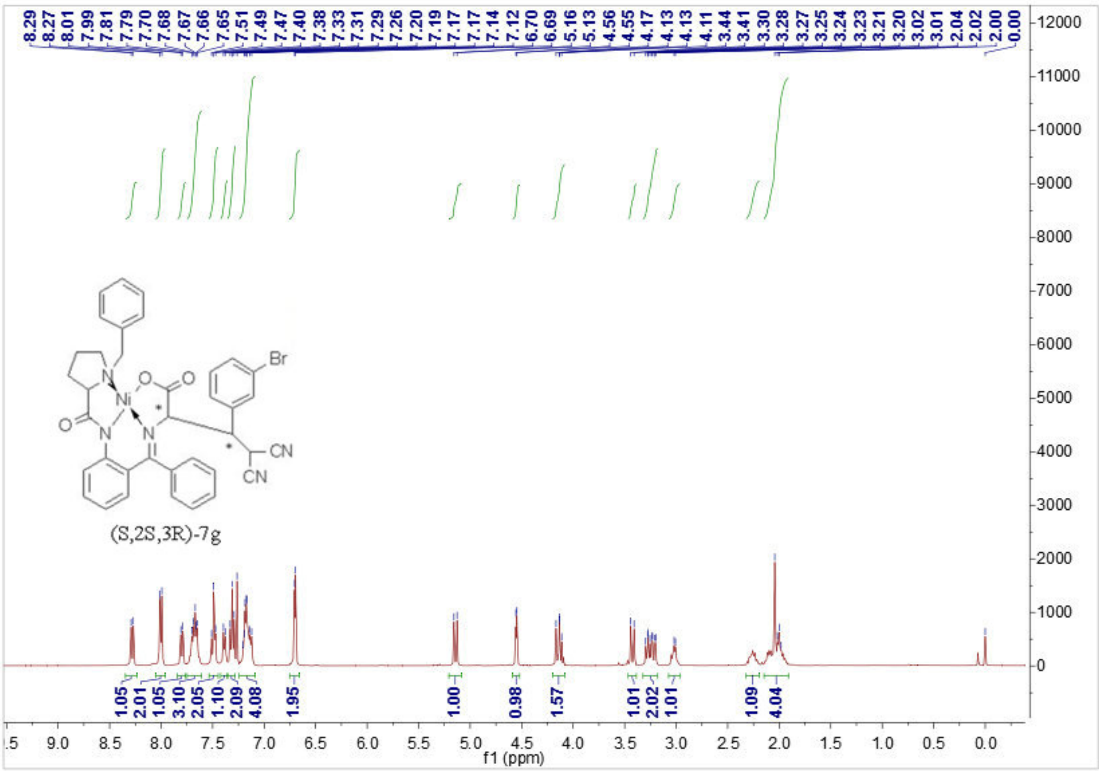

Figure S20. ^{13}C-NMR Spectrum of Compound **7g**.

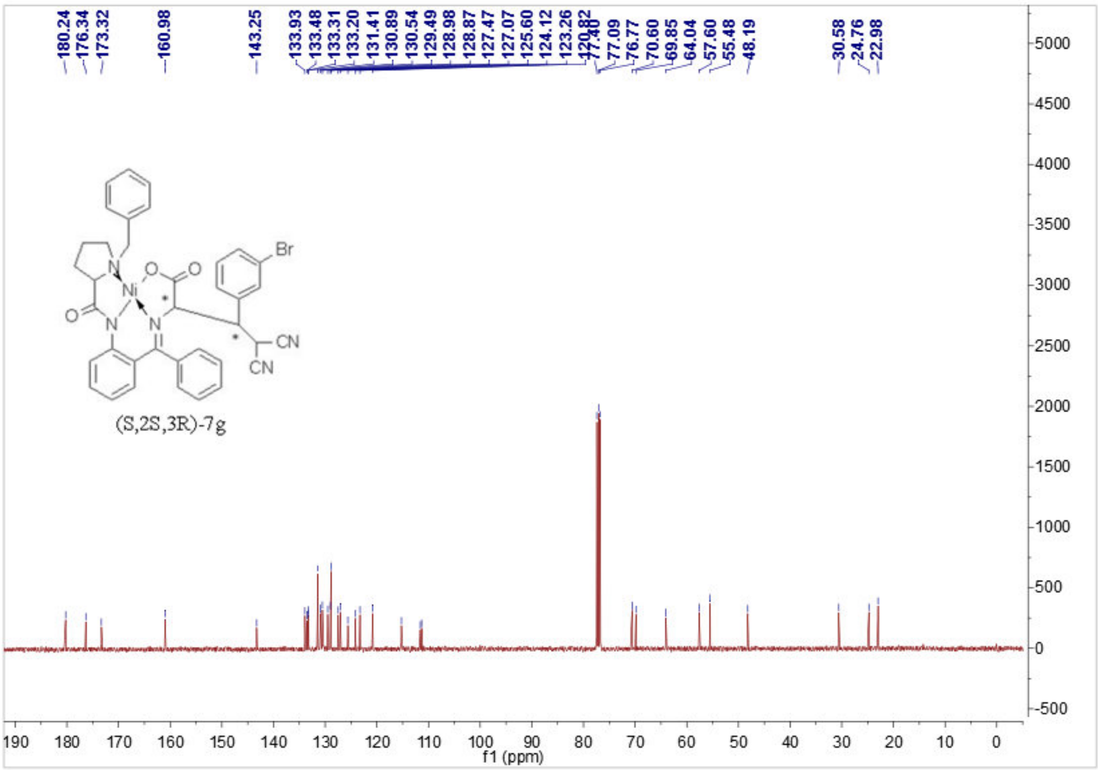

Figure S21. HPLC Spectra of Compound **7g**.

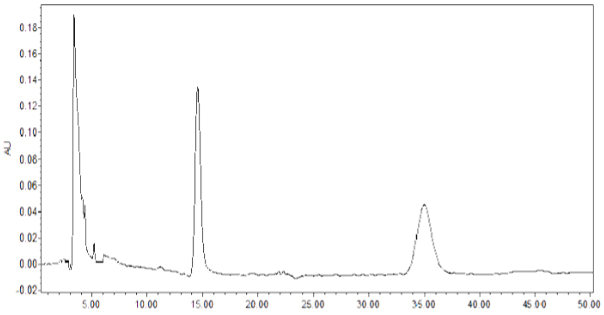

	rt(min)	Area	%Area	Type
1	14.527	4786692	49.74	bb
2	34.970	4835987	50.26	bb

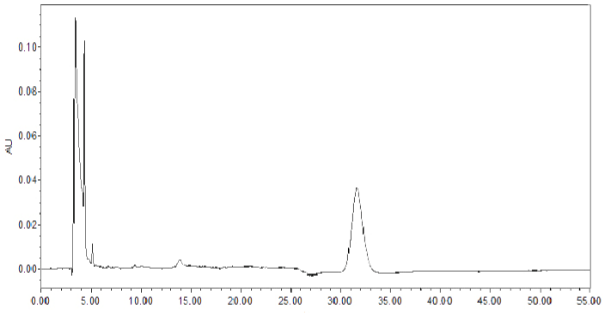

	rt(min)	Area	%Area	Type
1	13.790	68813	2.25	bb
2	31.615	2984636	97.75	bb

Figure S22. ^{1}H-NMR Spectrum of Compound **7h**.

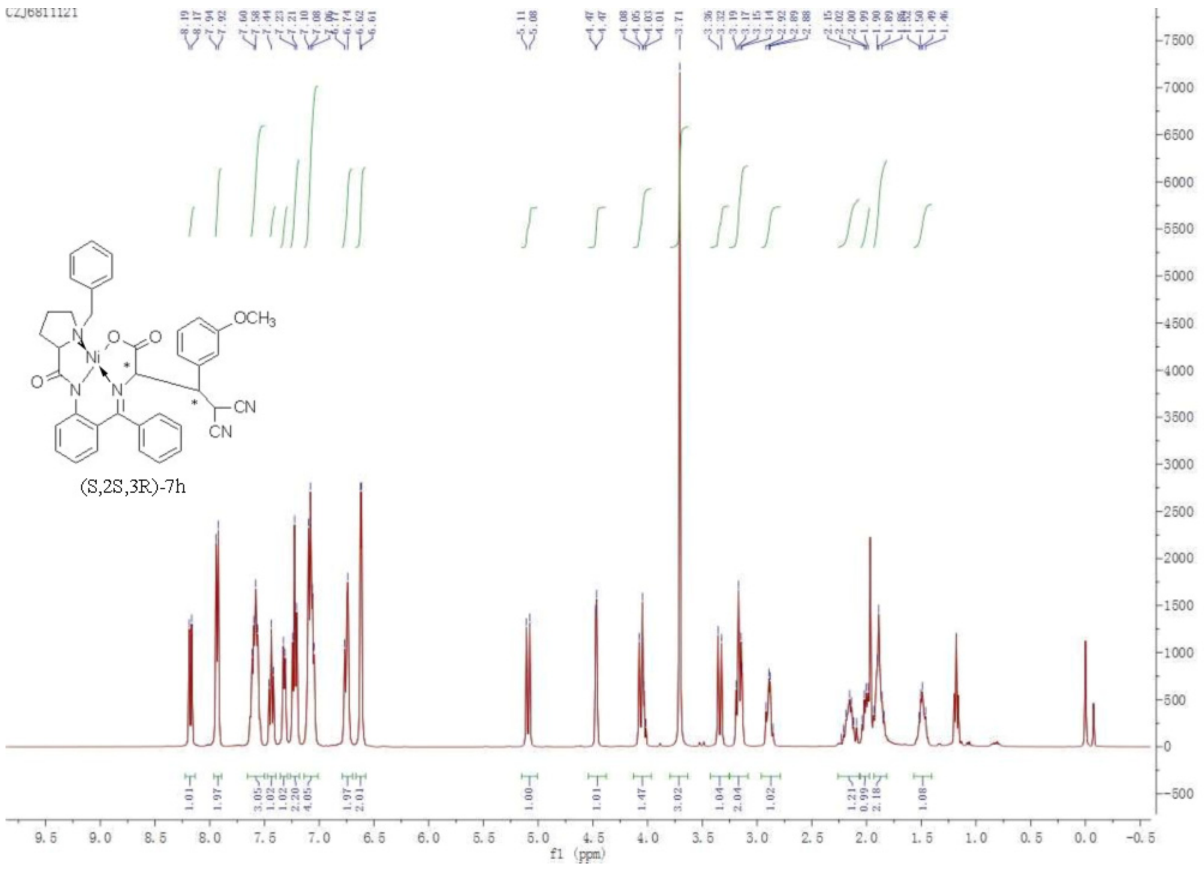

Figure S23. ^{13}C-NMR Spectrum of Compound **7h**.

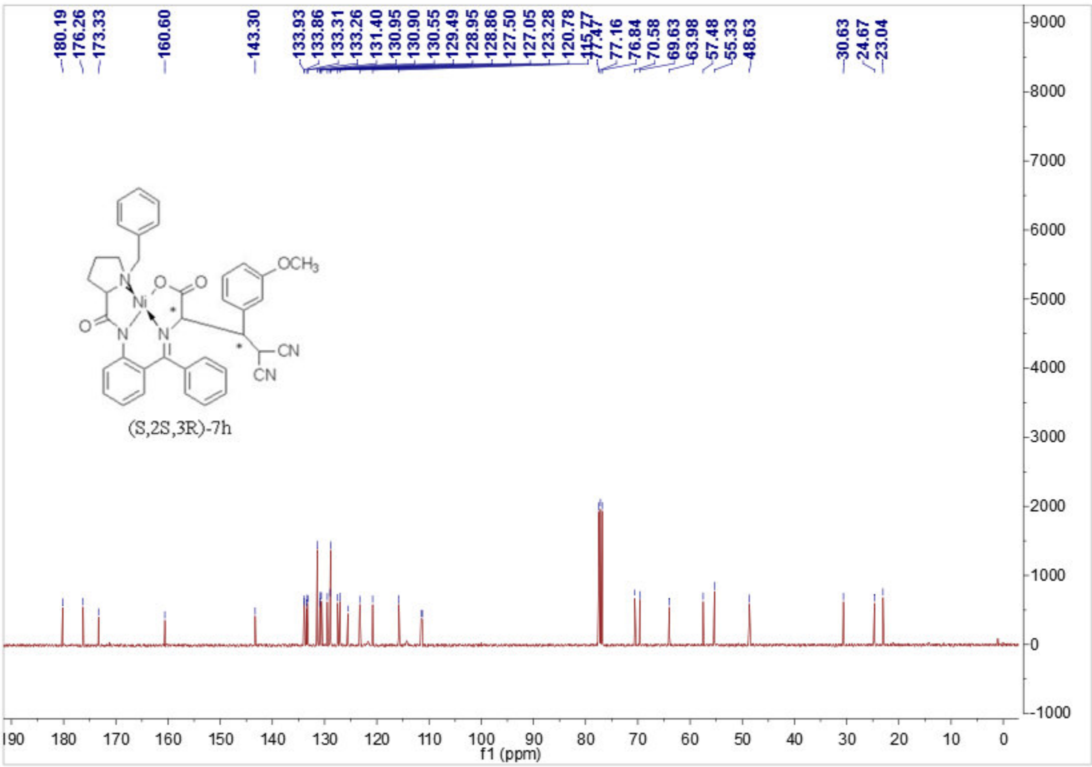

Figure S24. HPLC Spectra of Compound **7h**.

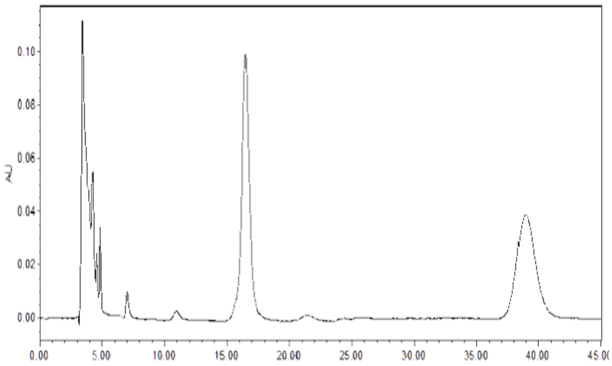

	rt(min)	Area	%Area	Type
1	16.488	4254231	50.93	bb
2	38.939	4099244	49.07	bb

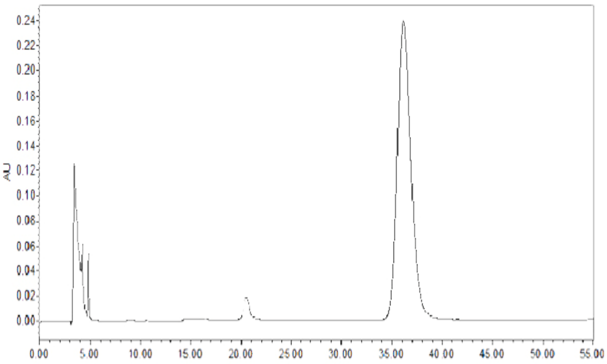

	rt(min)	Area	%Area	Type
1	10.524	3980	0.02	bb
2	36.142	21995468	99.98	bb

Figure S25. ^{1}H-NMR Spectrum of Compound **7i**.

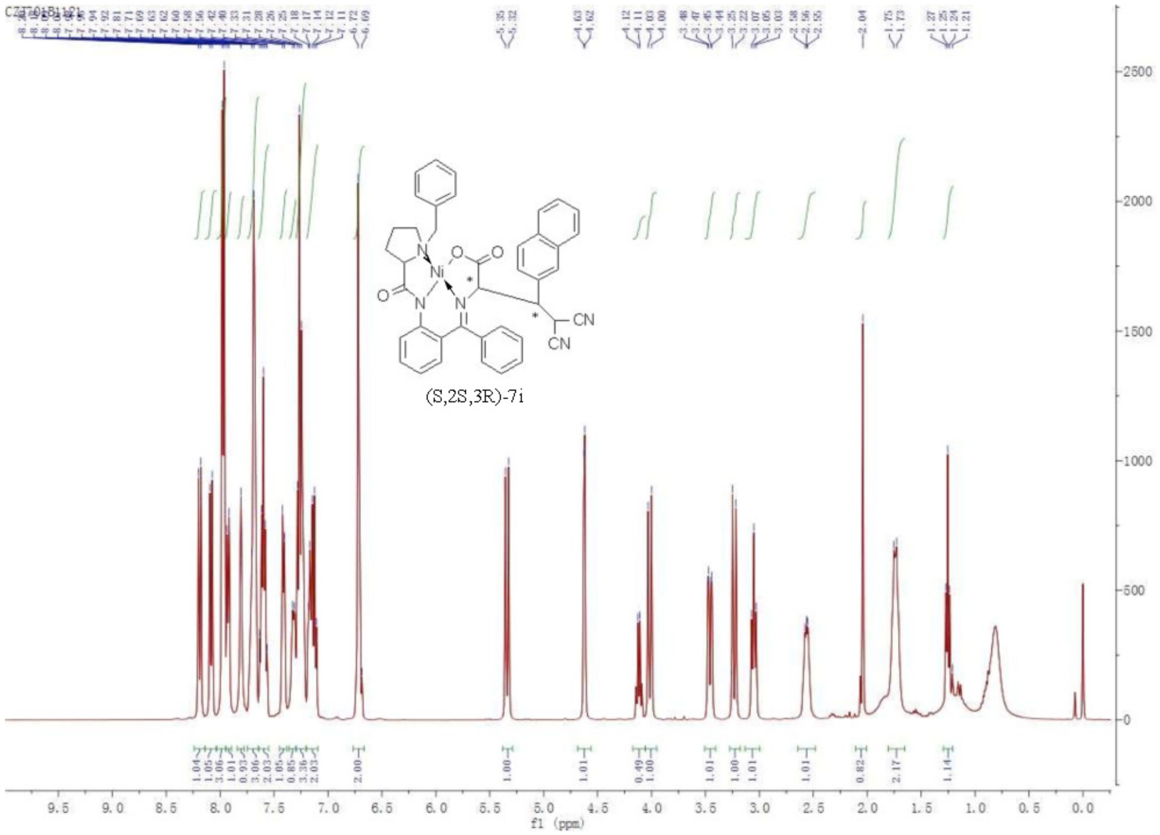

Figure S26. ^{13}C-NMR Spectrum of Compound **7i**.

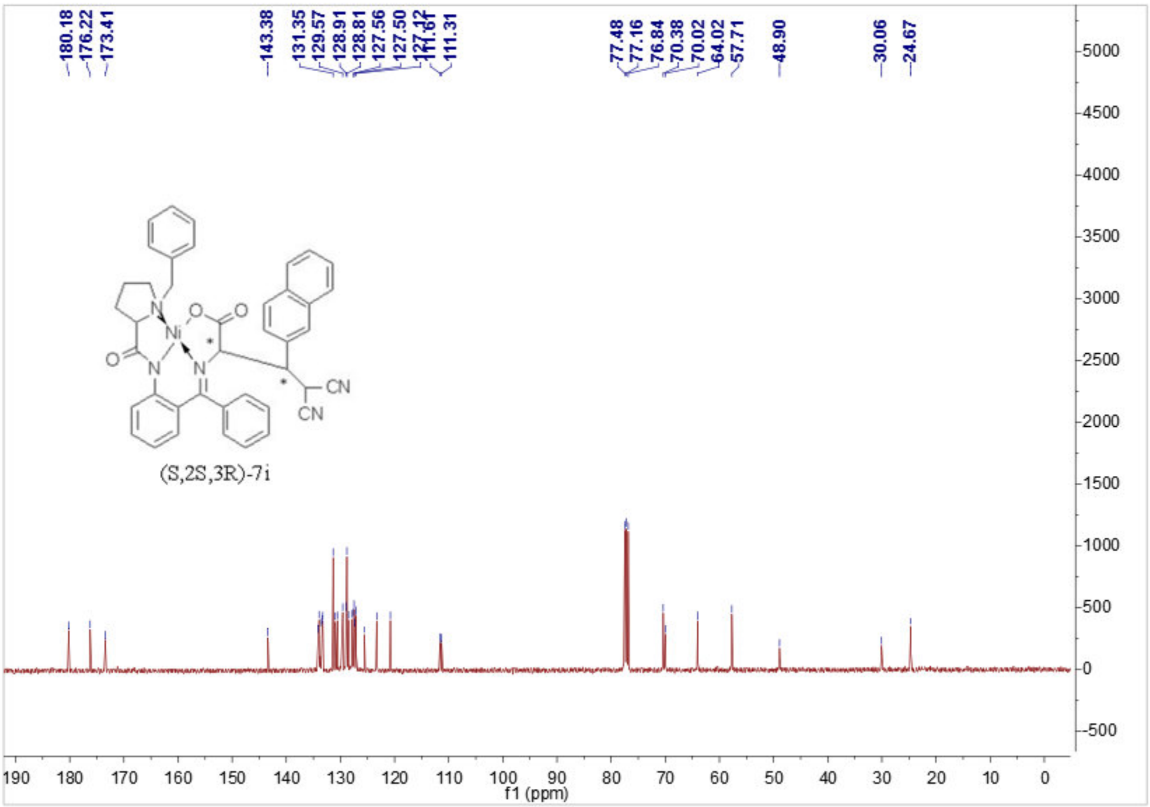

Figure S27. HPLC Spectra of Compound **7i**.

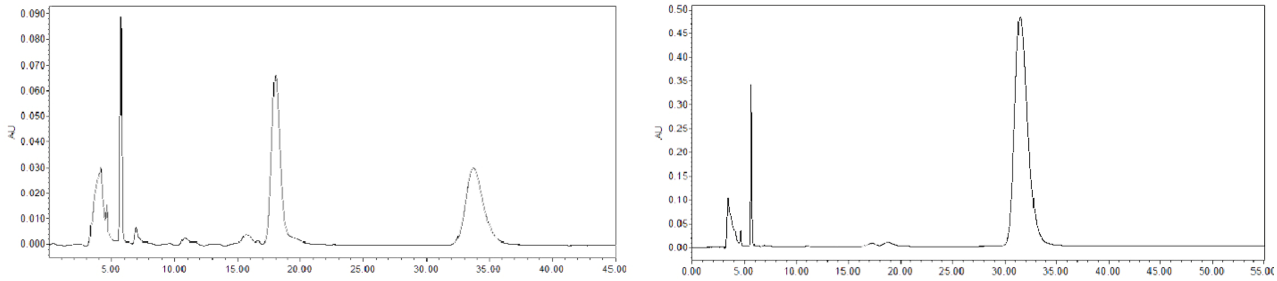

	rt(min)	Area	%Area	Type
1	17.989	9171285	49.38	bb
2	33.716	9400088	50.62	bb

	rt(min)	Area	%Area	Type
1	18.789	277770	0.66	bb
2	31.482	41895384	98.92	bb

Figure S28. ^{1}H-NMR Spectrum of Compound **7j**.

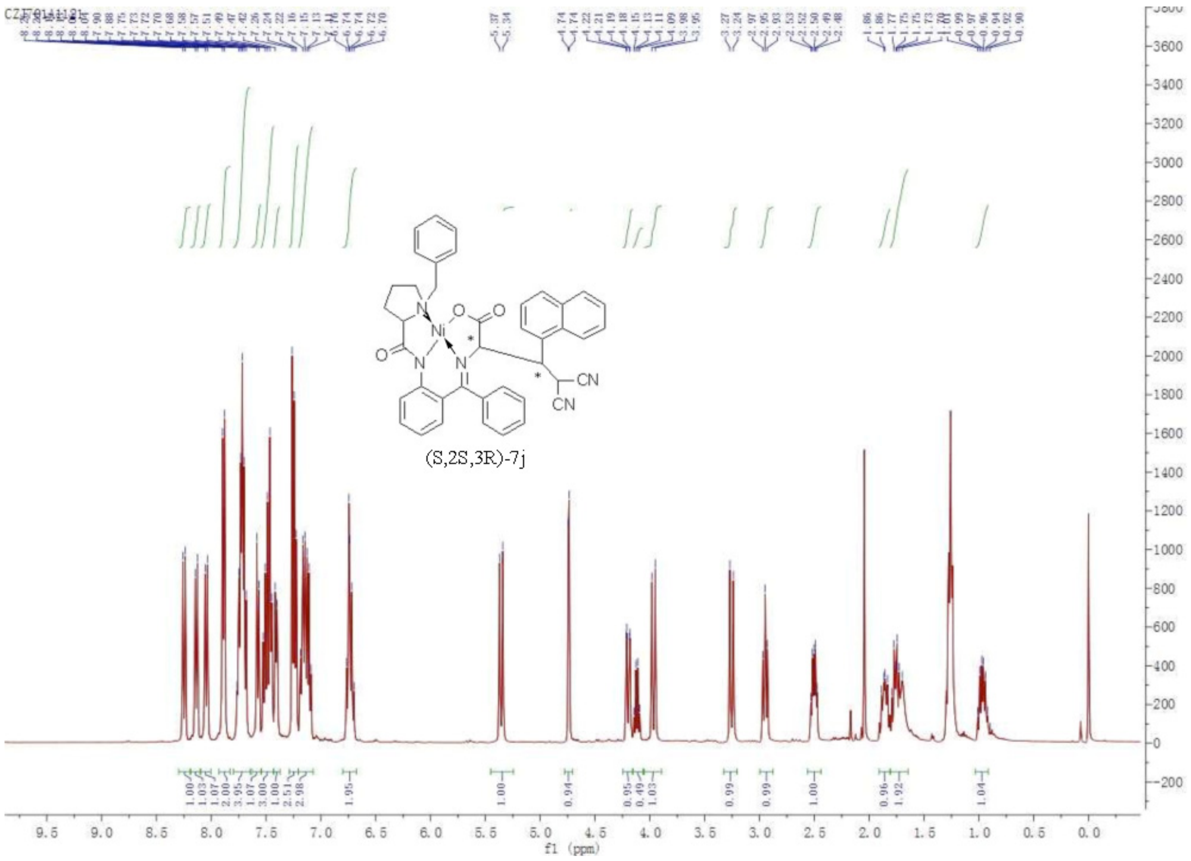

Figure S29. ^{13}C-NMR Spectrum of Compound **7j**.

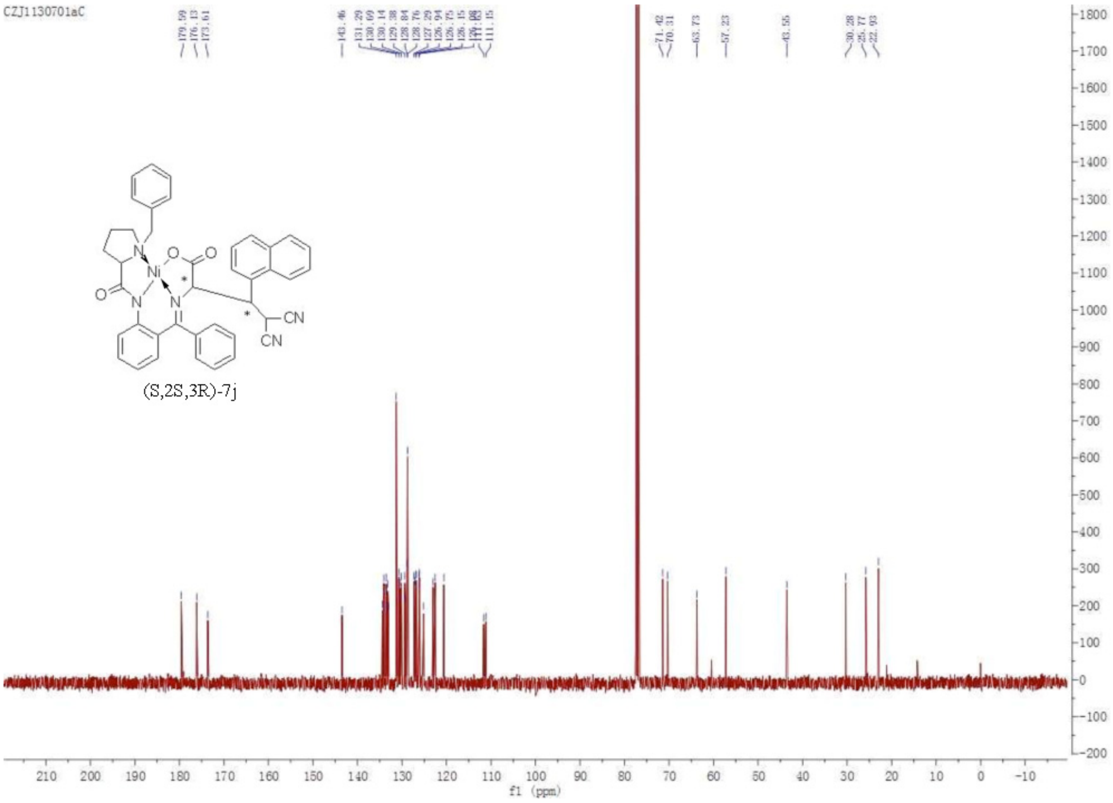

Figure S30. HPLC Spectra of Compound **7j**.

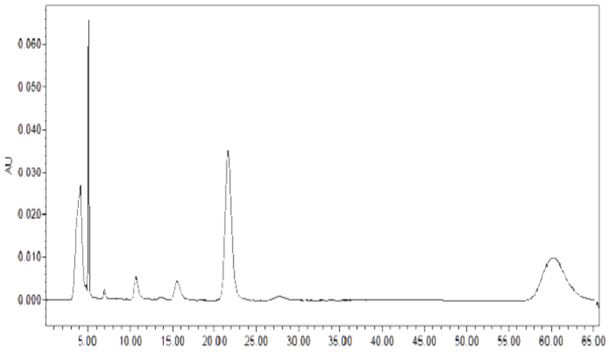

	rt(min)	Area	%Area	Type
1	21.627	7183201	50.80	bb
2	60.213	6957441	49.20	bb

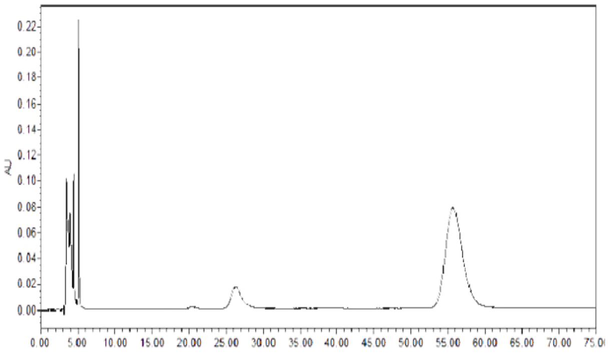

	rt(min)	Area	%Area	Type
1	20.432	64501	0.53	bb
2	55.723	12165426	99.47	bb

Figure S31. ^{1}H-NMR Spectrum of Compound **7k**.

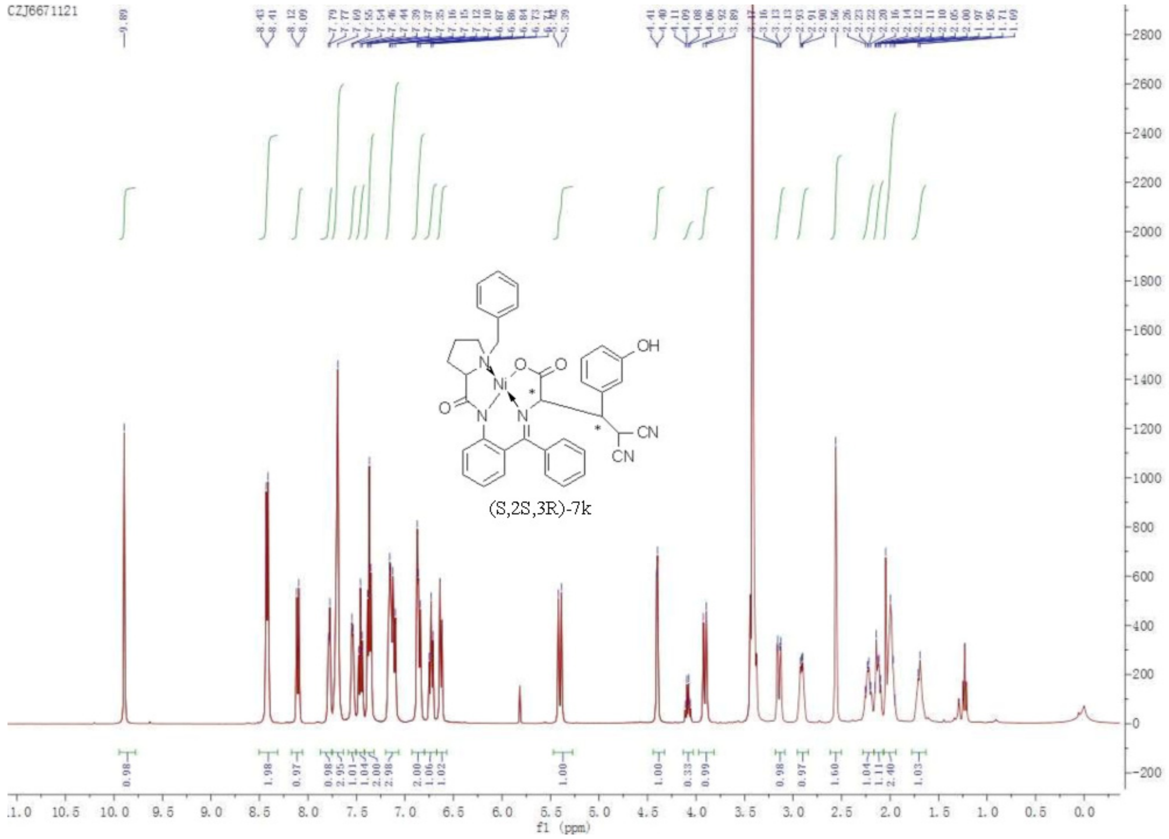

Figure S32. ^{13}C-NMR Spectrum of Compound **7k**.

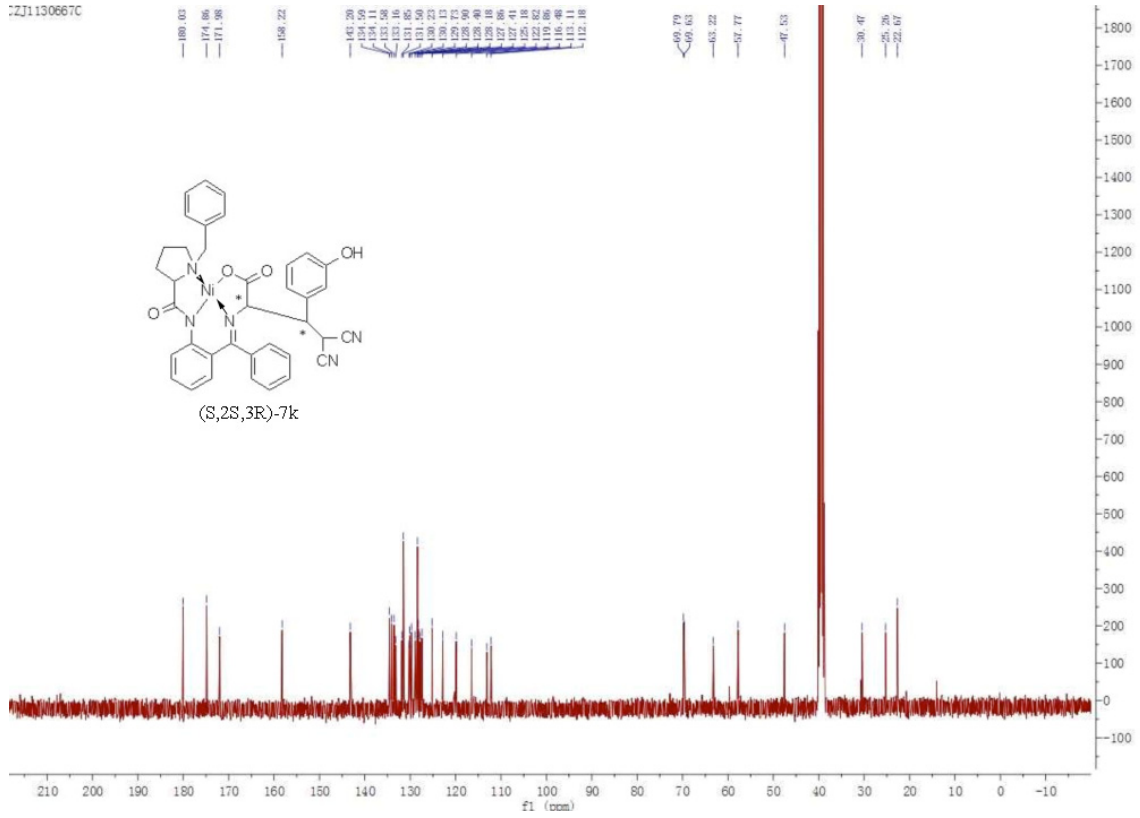

Figure S33. HPLC Spectra of Compound **7k**.

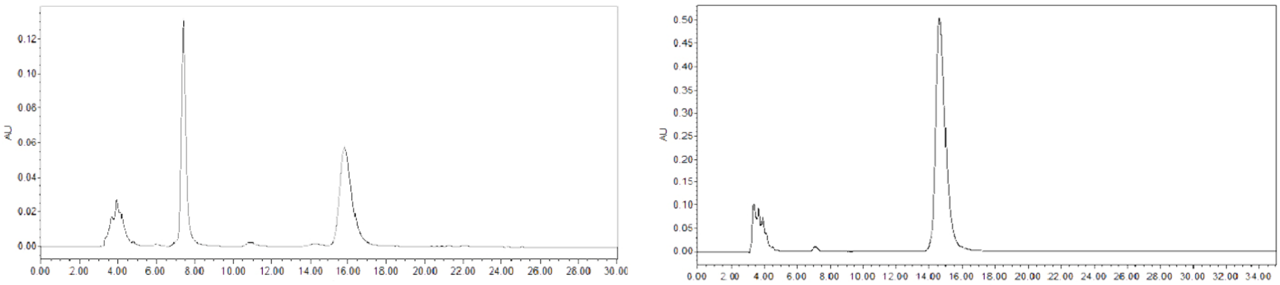

	rt(min)	Area	%Area	Type
1	7.386	6826800	49.71	bb
2	15.843	6905386	50.29	bb

	rt(min)	Area	%Area	Type
1	7.106	143702	0.74	bb
2	14.614	19405801	99.26	bb

Figure S34. ^{1}H-NMR Spectrum of Compound **7l**.

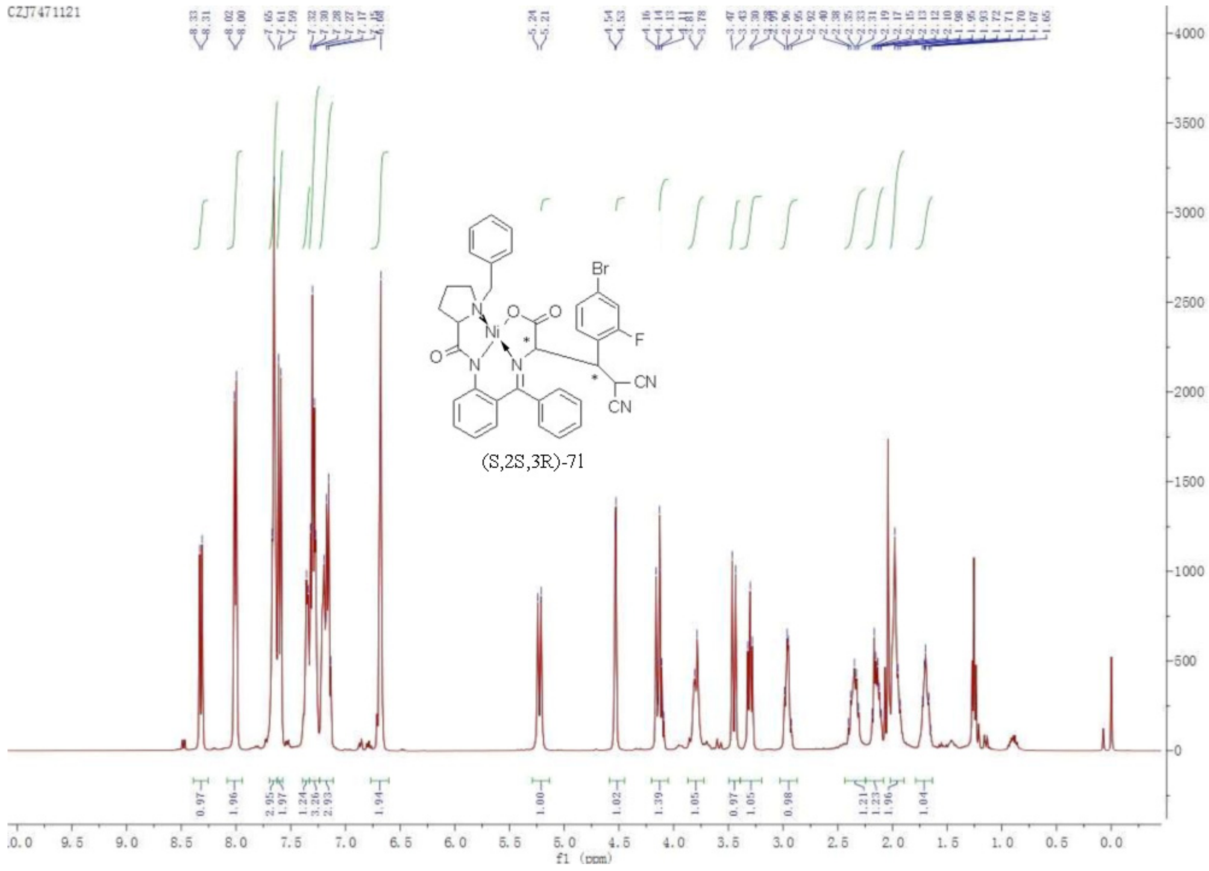

Figure S35. ^{13}C-NMR Spectrum of Compound **7l**.

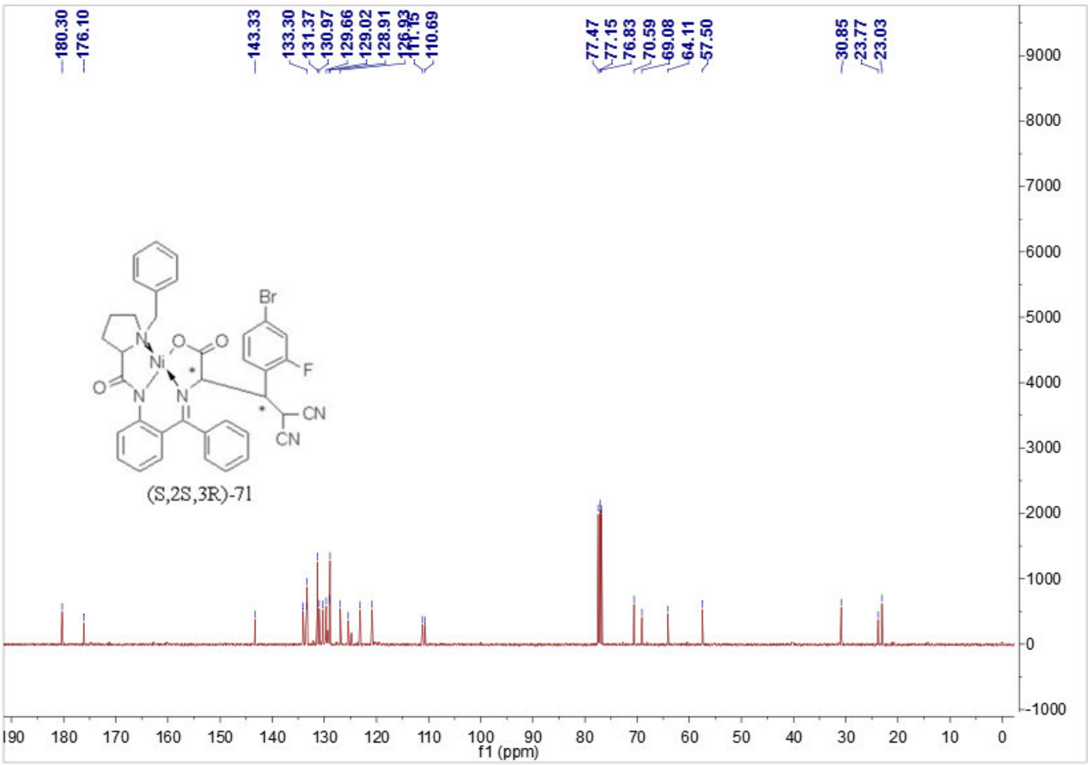

Figure S36. HPLC Spectra of Compound **7l**.

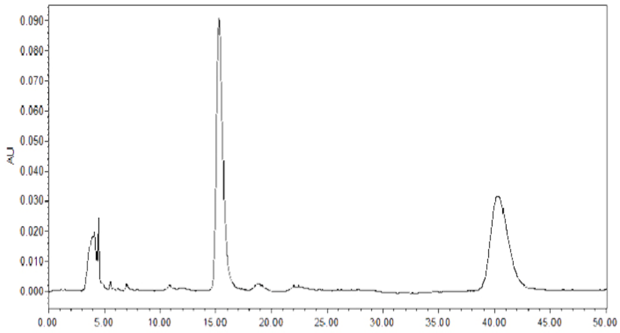

	rt(min)	Area	%Area	Type
1	15.320	9256719	50.75	bb
2	40.302	8984830	49.25	bb

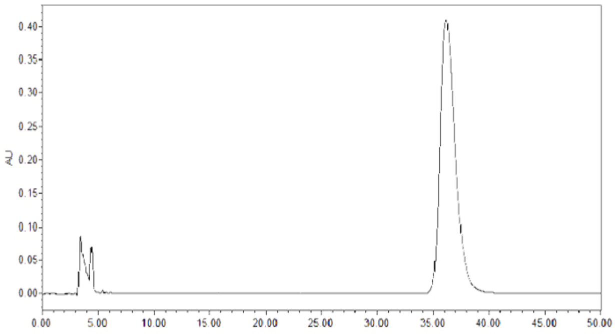

	rt(min)	Area	%Area	Type
1	36.156	38190822	100.00	bb

Figure S37. ^{1}H-NMR Spectrum of Compound **7m**.

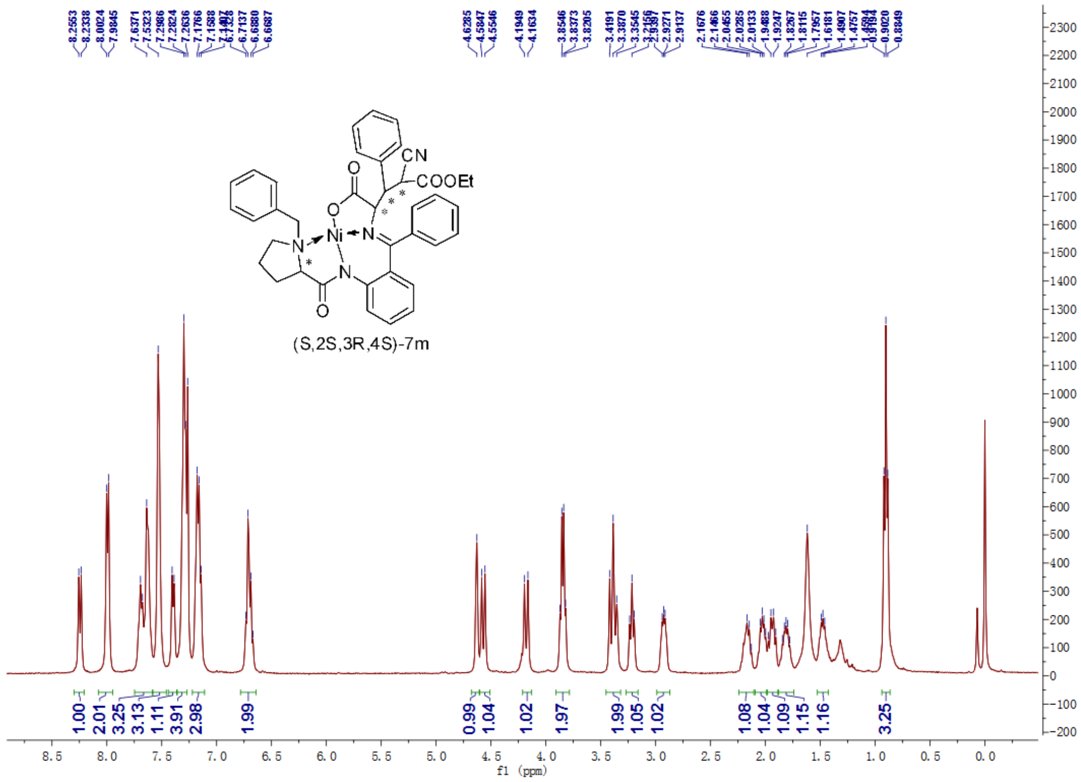

Figure S38. ^{13}C-NMR Spectrum of Compound **7m**.

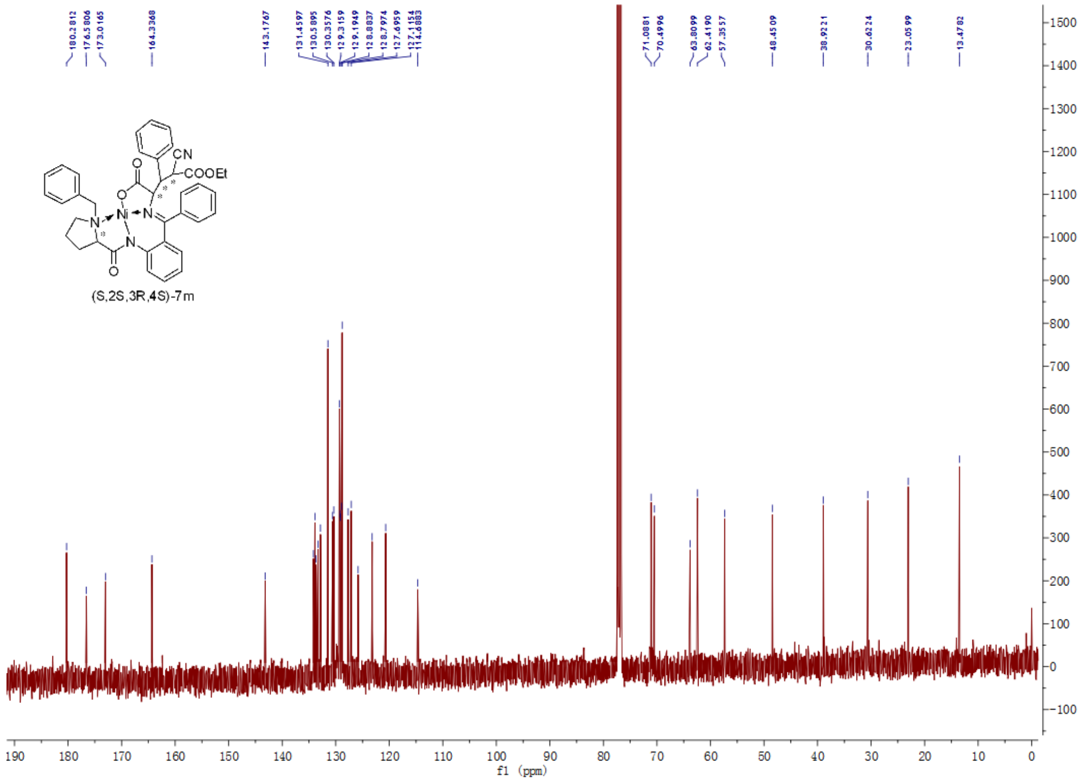

Figure S39. HPLC Spectra of Compound **7m**.

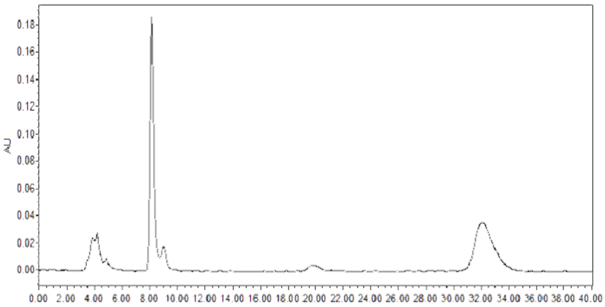

	rt(min)	Area	%Area	Type
1	8.140	9384010	51.57	bb
2	32.078	8812711	48.43	bb

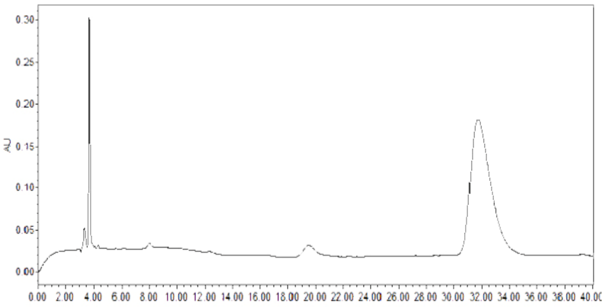

	rt(min)	Area	%Area	Type
1	8.012	62931	0.58	bb
2	31.696	10844580	99.42	bb

Figure S40. ^{1}H-NMR Spectrum of Compound **7n**.

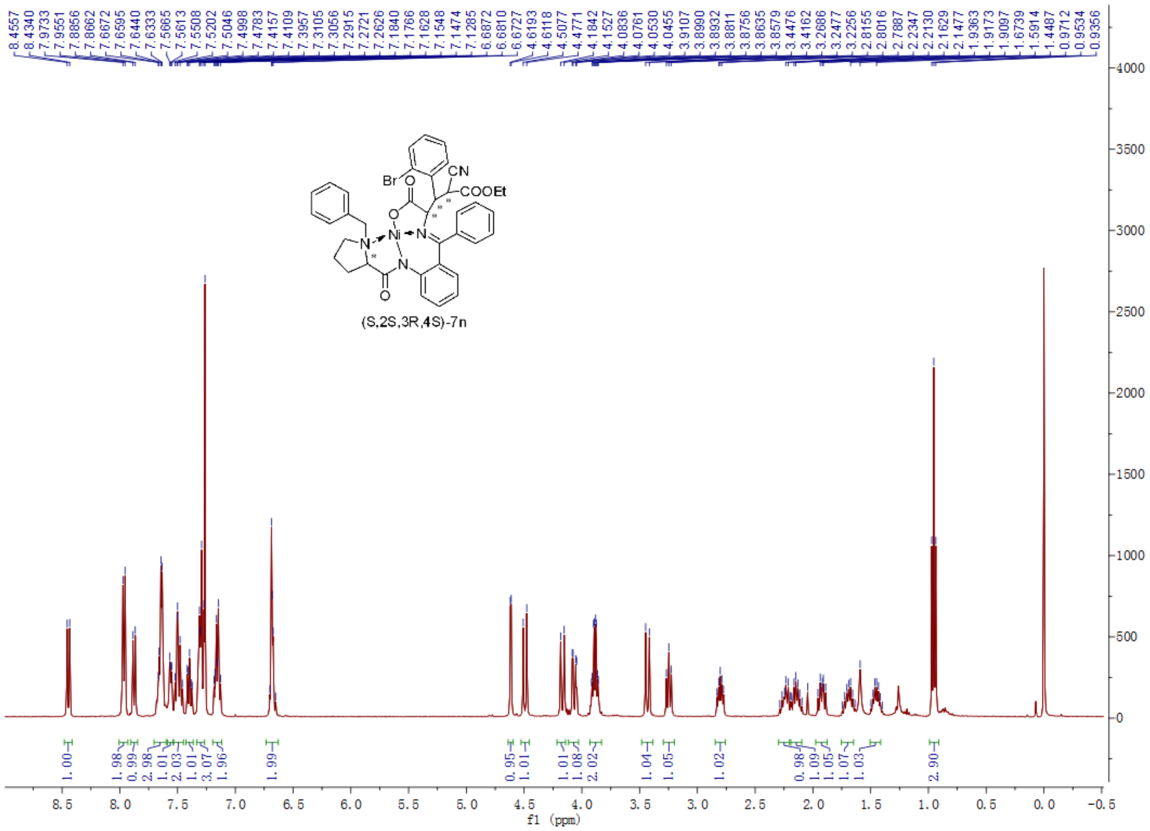

Figure S41. ^{13}C-NMR Spectrum of Compound **7n**.

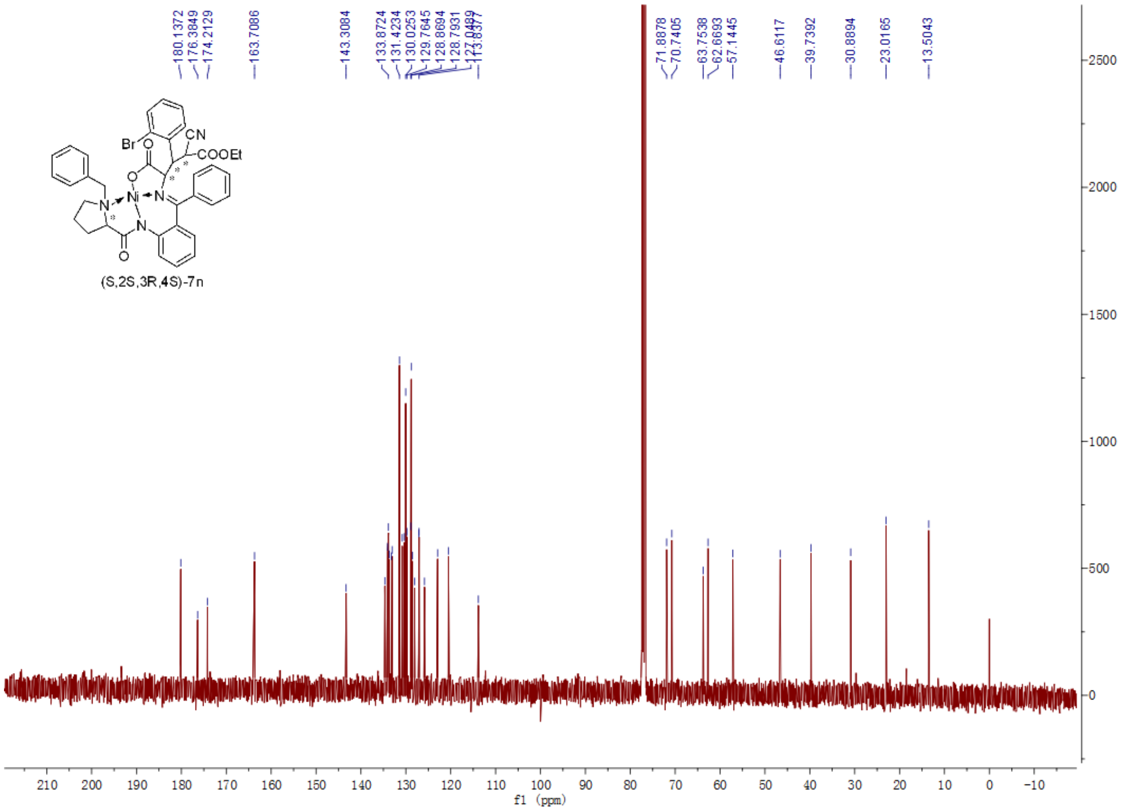

Figure S42. HPLC Spectra of Compound **7n**.

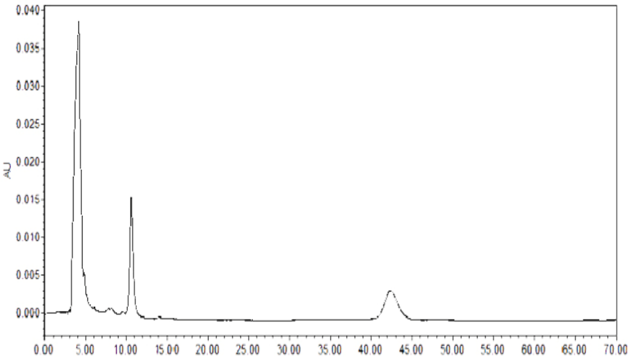

	rt(min)	Area	%Area	Type
1	10.600	867926	51.93	bb
2	42.394	803498	48.07	bb

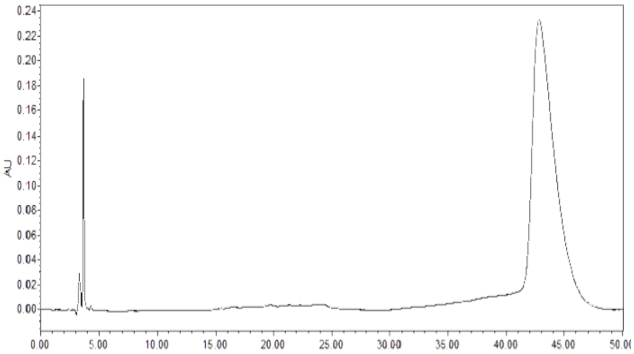

	rt(min)	Area	%Area	Type
1	42.821	29283144	100.00	bb

Figure S43. ^{1}H-NMR Spectrum of Compound **7o**.

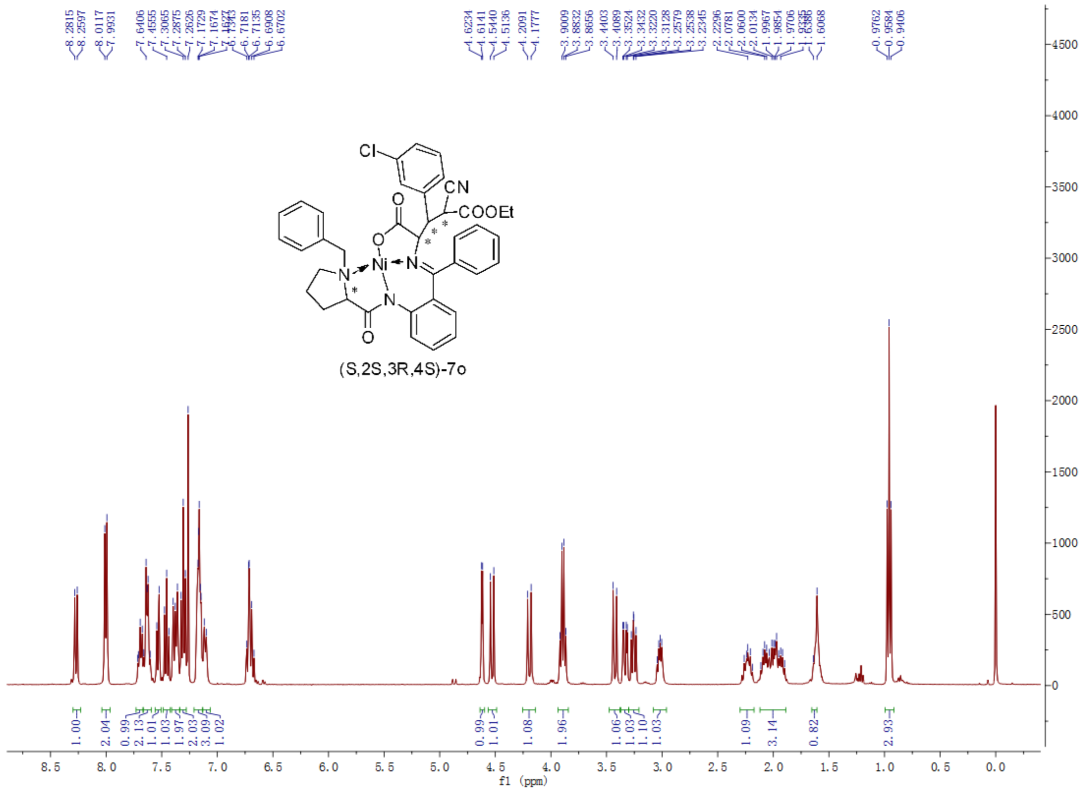

Figure S44. ^{13}C-NMR Spectrum of Compound **7o**.

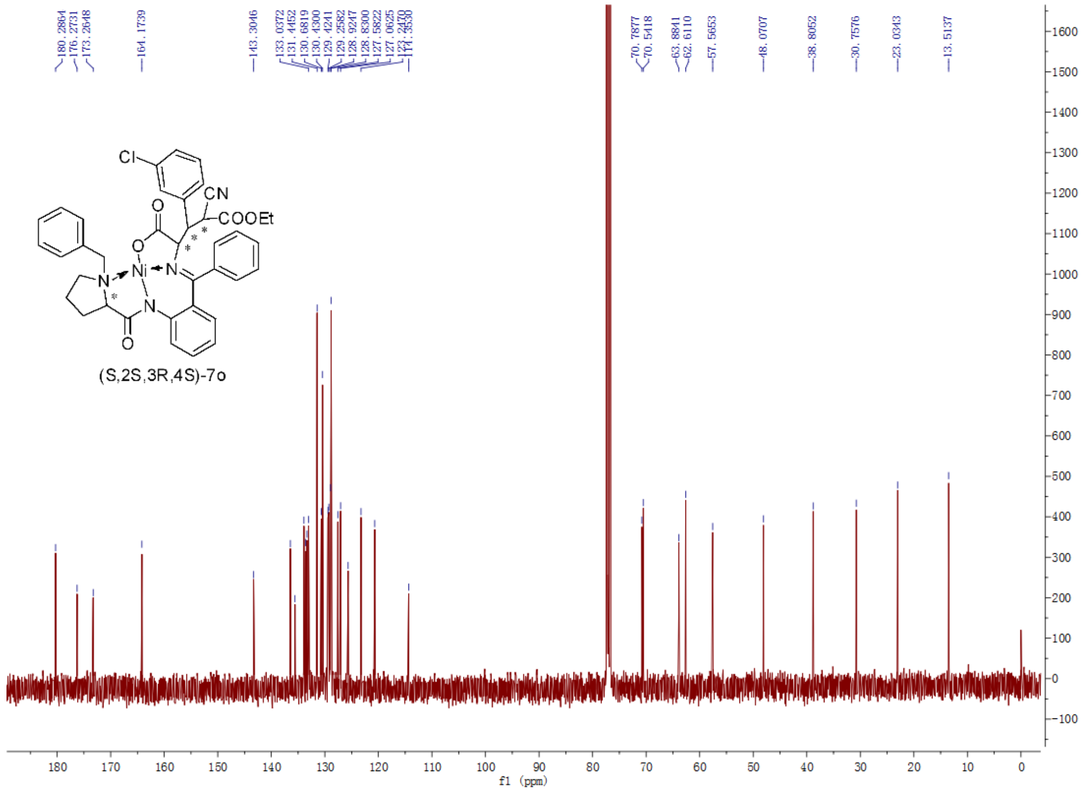

Figure S45. HPLC Spectra of Compound **7o**.

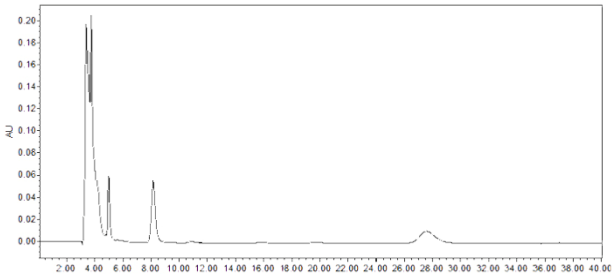

	rt(min)	Area	%Area	Type
1	8.165	875235	51.33	bb
2	27.564	829911	48.67	bb

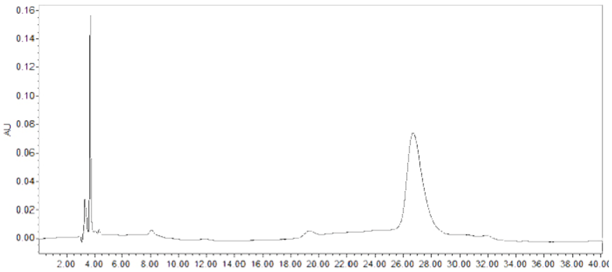

	rt(min)	Area	%Area	Type
1	8.015	58531	1.08	bb
2	26.685	5350807	98.92	bb

Figure S46. ^{1}H-NMR Spectrum of Compound **7p**.

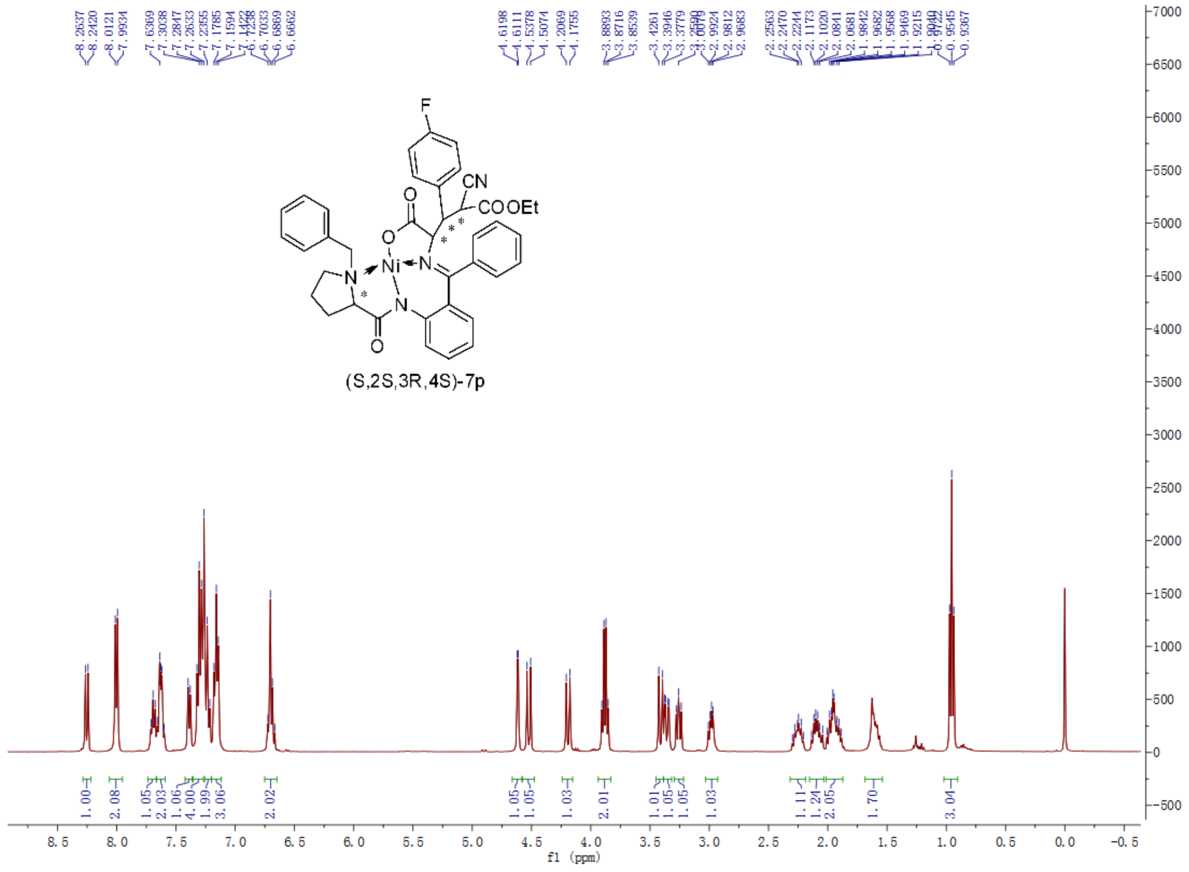

Figure S47. ^{13}C-NMR Spectrum of Compound **7p**.

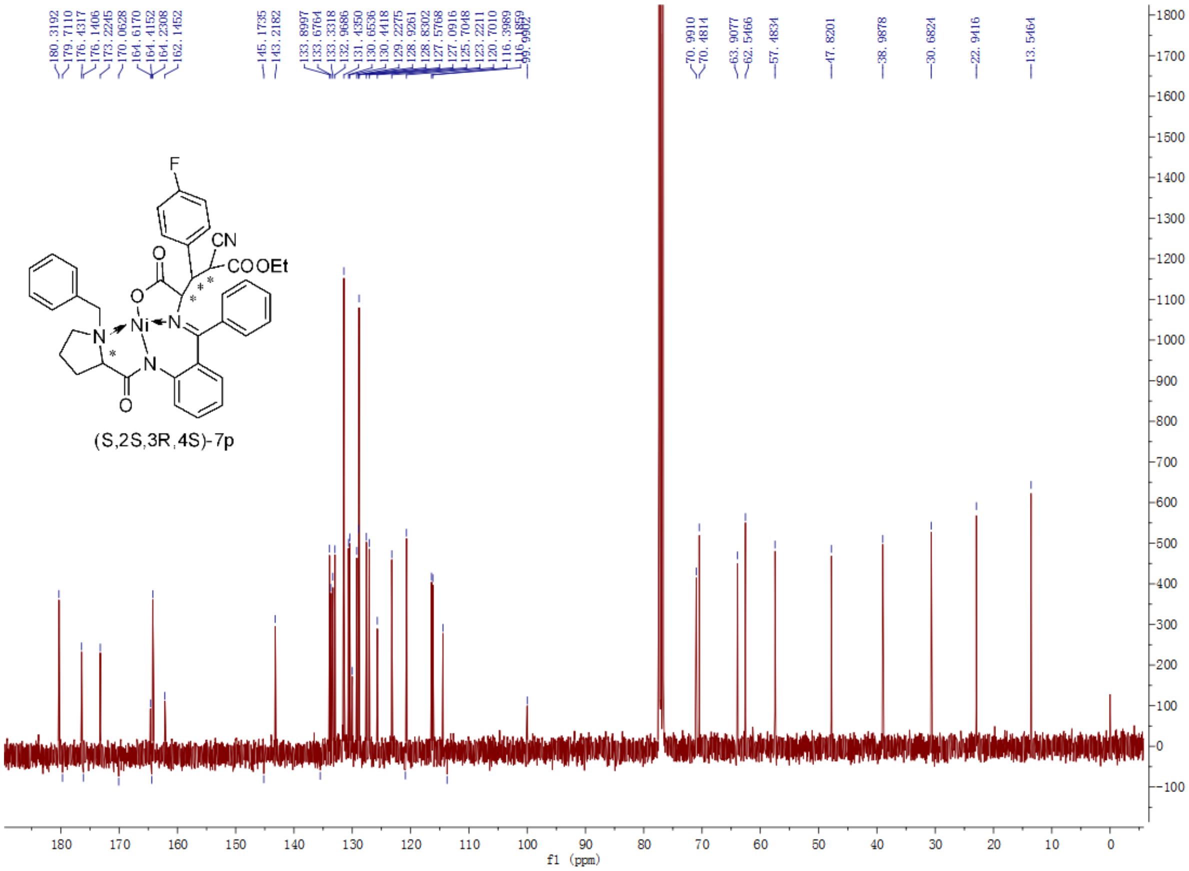

Figure S48. HPLC Spectra of Compound **7p**.

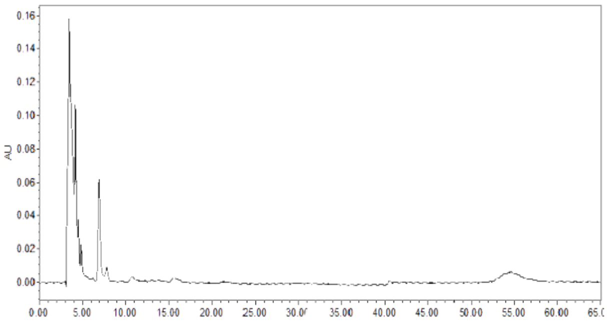

	rt(min)	Area	%Area	Type
1	6.918	929574	51.16	bb
2	54.504	887400	48.84	bb

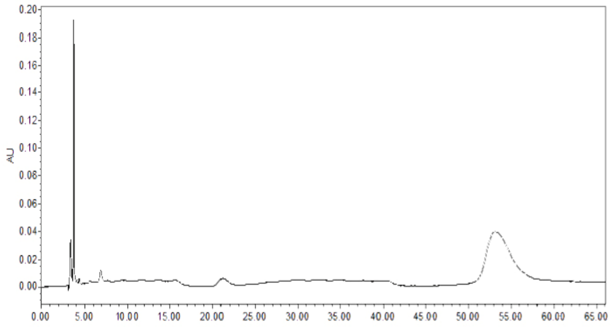

	rt(min)	Area	%Area	Type
1	6.856	127507	1.81	bb
2	53.169	6899978	98.19	bb

Figure S49. ^{1}H-NMR Spectrum of Compound **7q**.

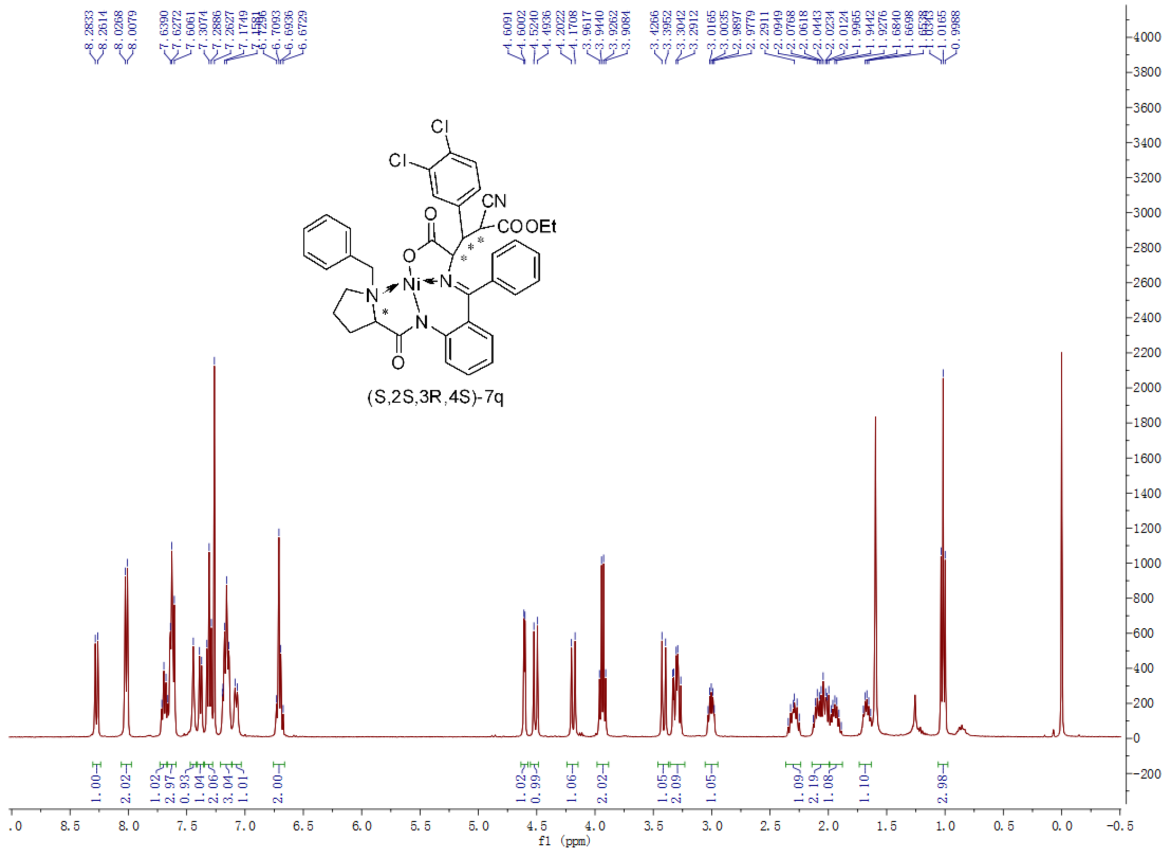

Figure S50. ^{13}C-NMR Spectrum of Compound **7q**.

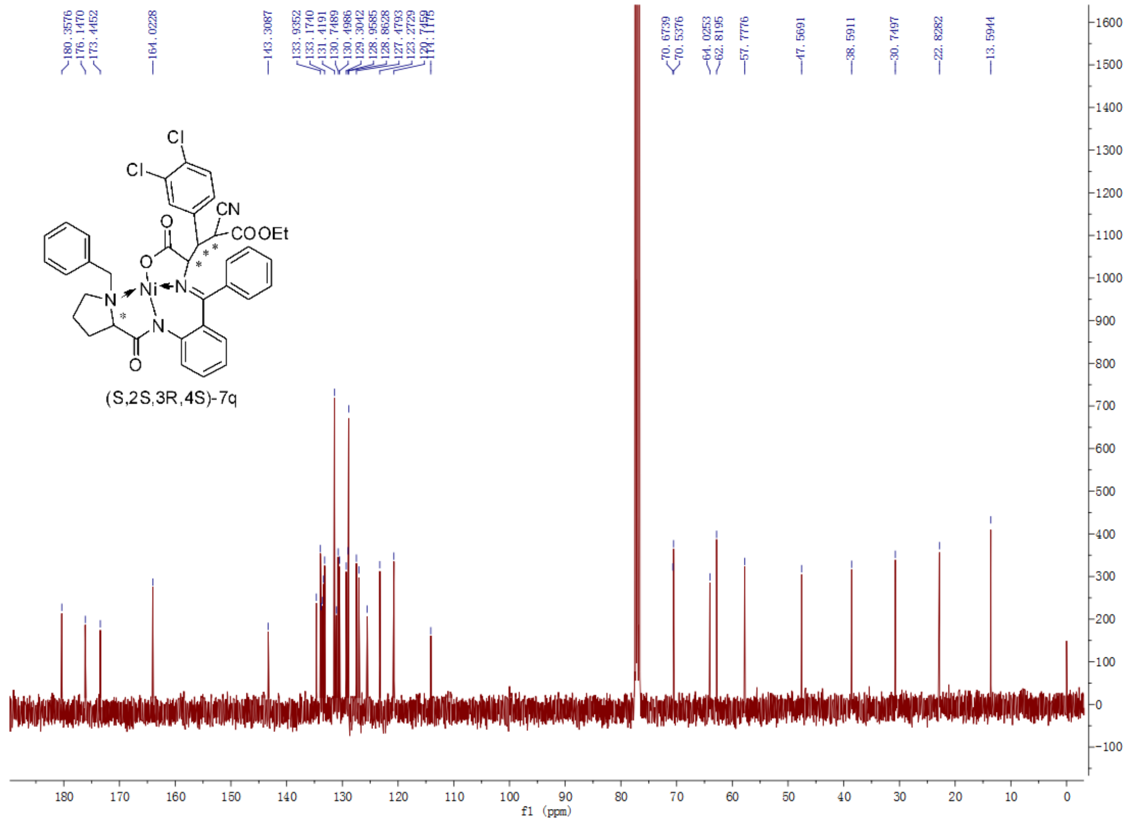

Figure S51. HPLC Spectra of Compound **7q**.

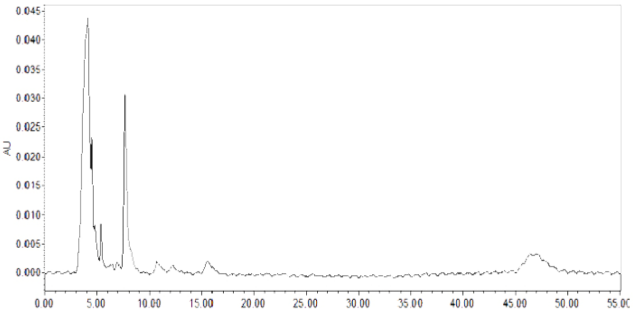

	rt(min)	Area	%Area	Type
1	7.619	1343205	53.50	bb
2	46.512	1167239	46.50	bb

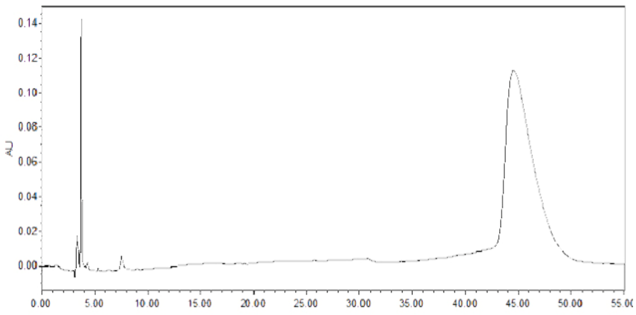

	rt(min)	Area	%Area	Type
1	7.564	135012	0.70	bb
2	44.529	19080822	99.30	bb

Figure S52. ^{1}H-NMR Spectrum of Compound **7r**.

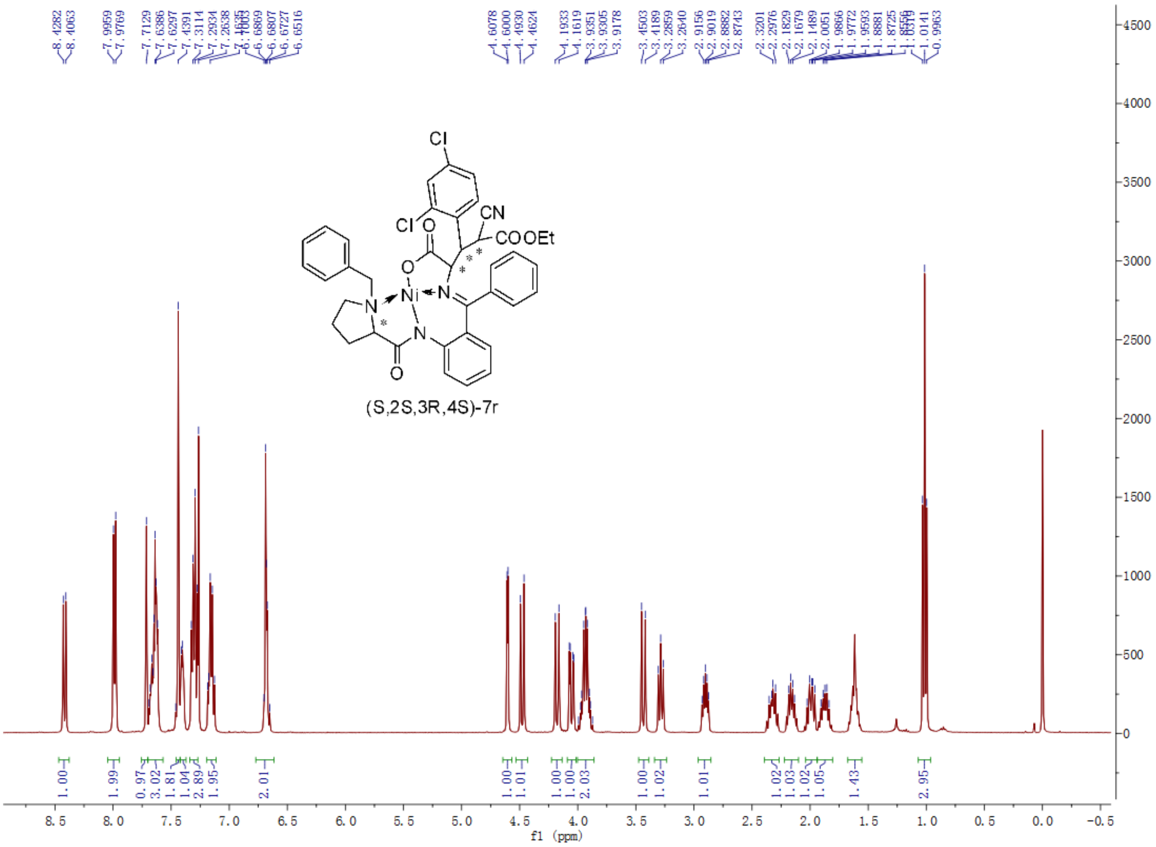

Figure S53. ^{13}C-NMR Spectrum of Compound **7r**.

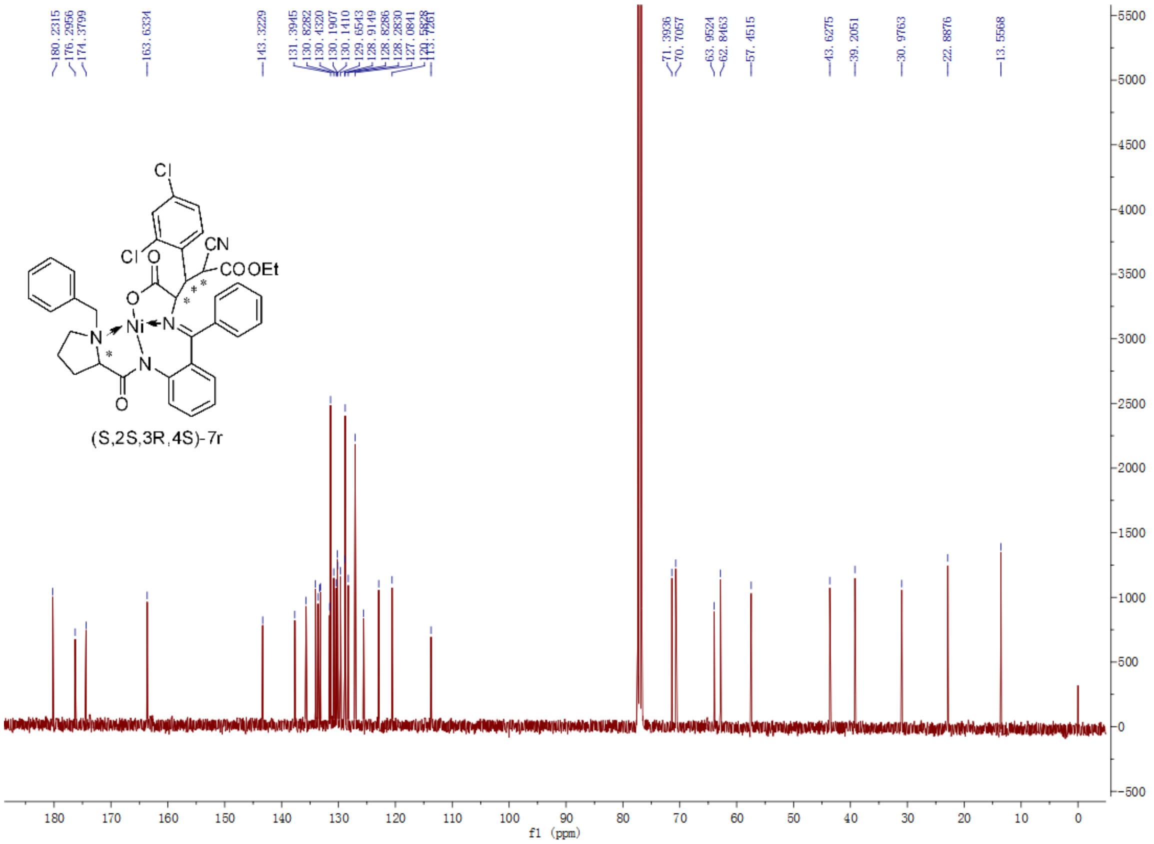

Figure S54. HPLC Spectra of Compound **7r**.

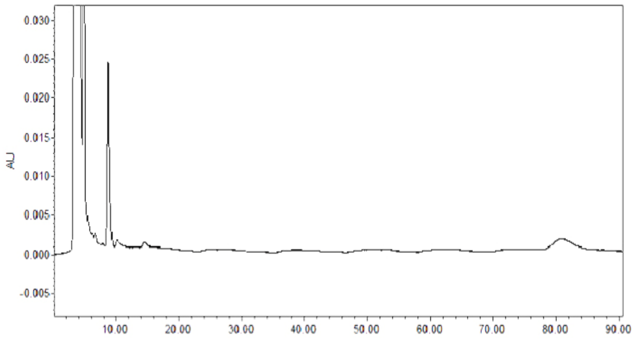

	rt(min)	Area	%Area	Type
1	8.756	235785	52.71	bb
2	80.848	211515	47.29	bb

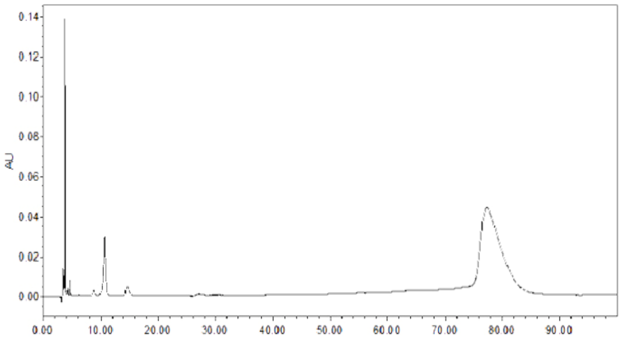

	rt(min)	Area	%Area	Type
1	8.752	96633	0.67	bb
2	77.290	14305717	99.33	bb

Figure S55. ^{1}H-NMR Spectrum of Compound **7s**.

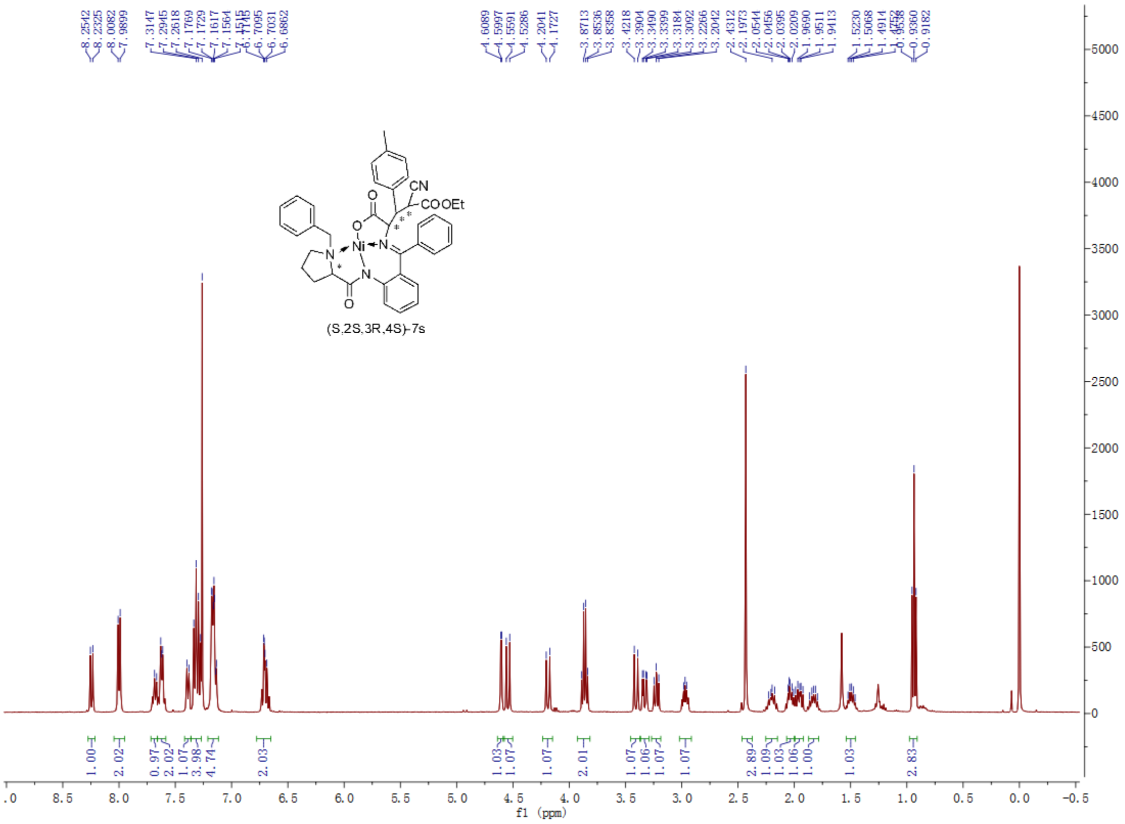

Figure S56. ^{13}C-NMR Spectrum of Compound **7s**.

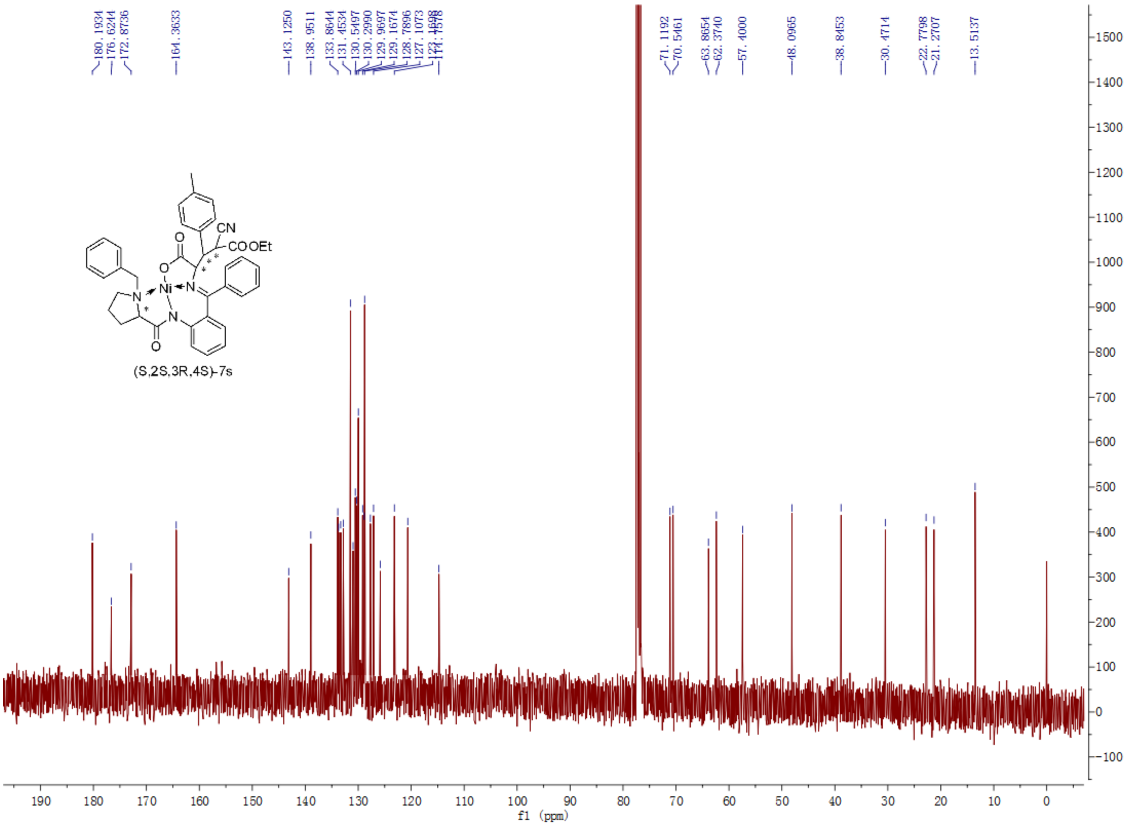

Figure S57. HPLC Spectra of Compound **7s**.

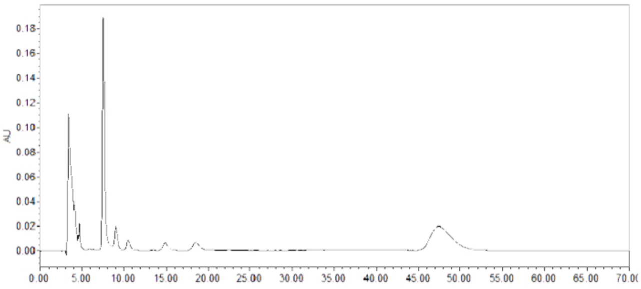

	rt(min)	Area	%Area	Type
1	7.533	3383008	50.88	bb
2	47.479	3265924	49.12	bb

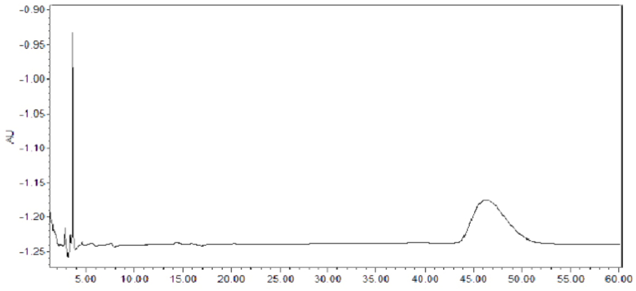

	rt(min)	Area	%Area	Type
1	46.325	20713278	100.00	bb

Figure S58. ^{1}H-NMR Spectrum of Compound **7t**.

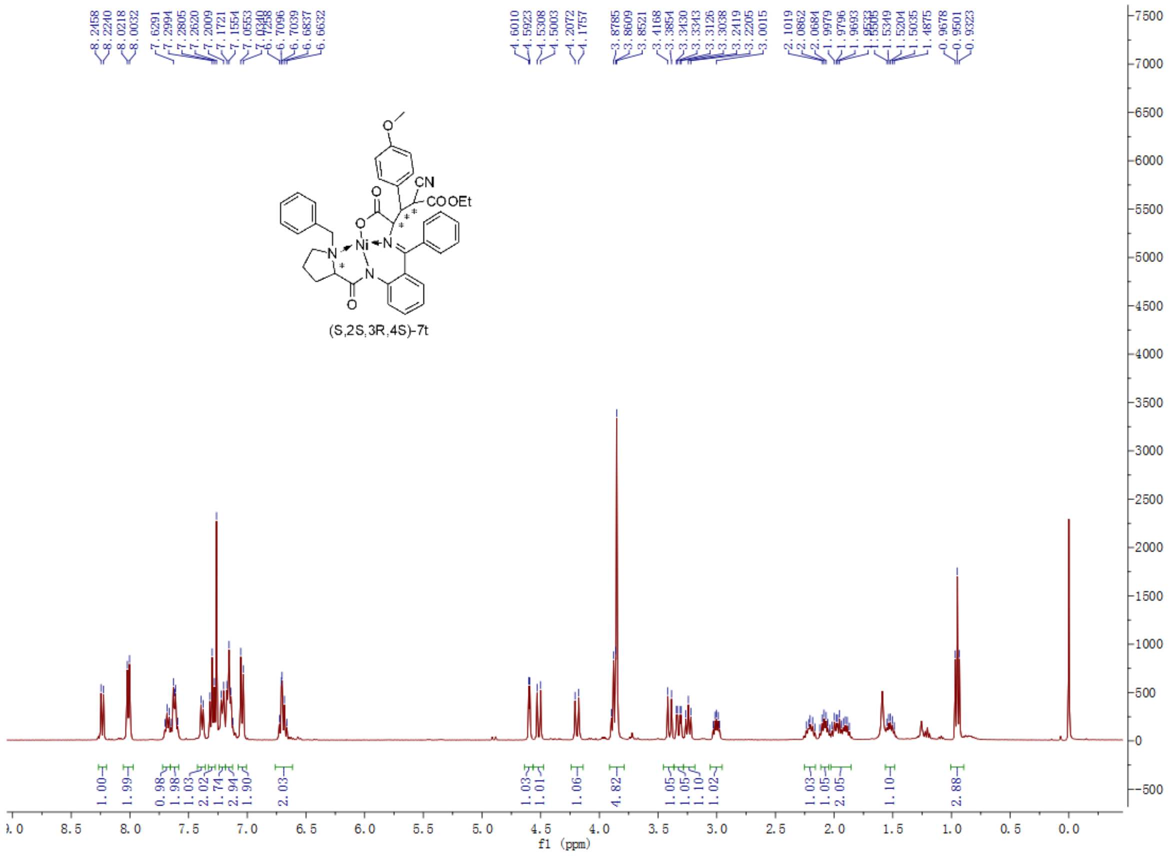

Figure S59. ^{13}C-NMR Spectrum of Compound **7t**.

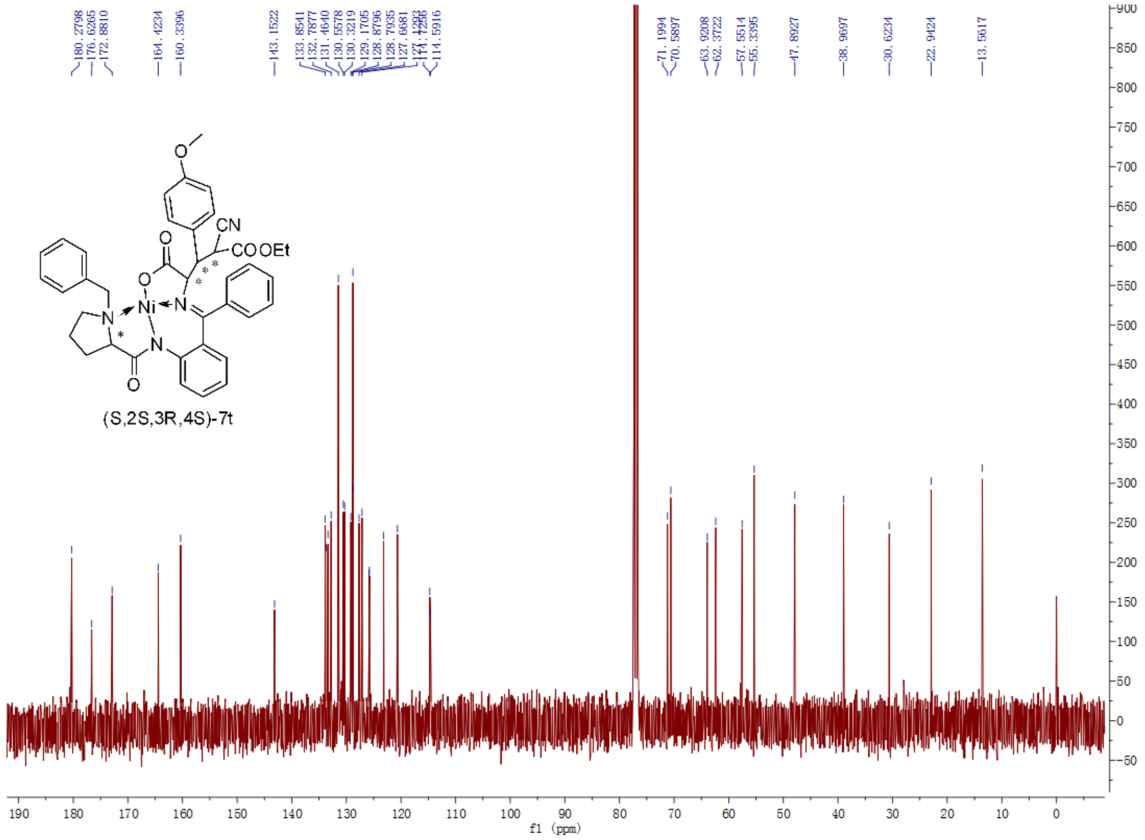

Figure S60. HPLC Spectra of Compound **7t**.

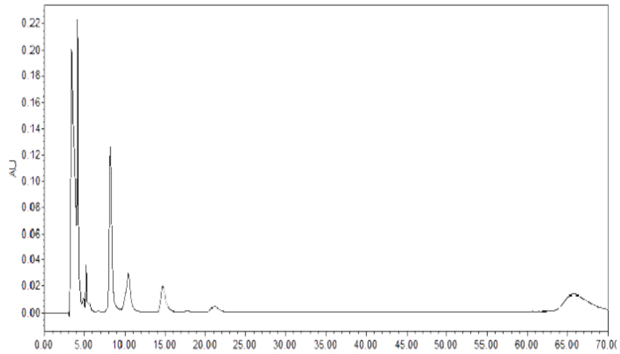

	rt(min)	Area	%Area	Type
1	8.234	2437737	51.51	bb
2	65.766	2295202	48.49	bb

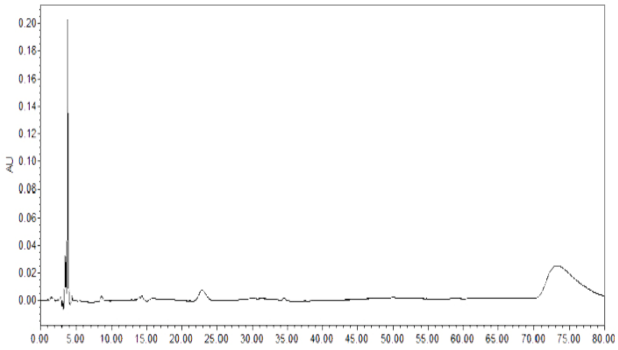

	rt(min)	Area	%Area	Type
1	8.548	71292	1.11	bb
2	73.210	6369366	98.89	bb

Figure S61. ^{1}H-NMR Spectrum of Compound **7u**.

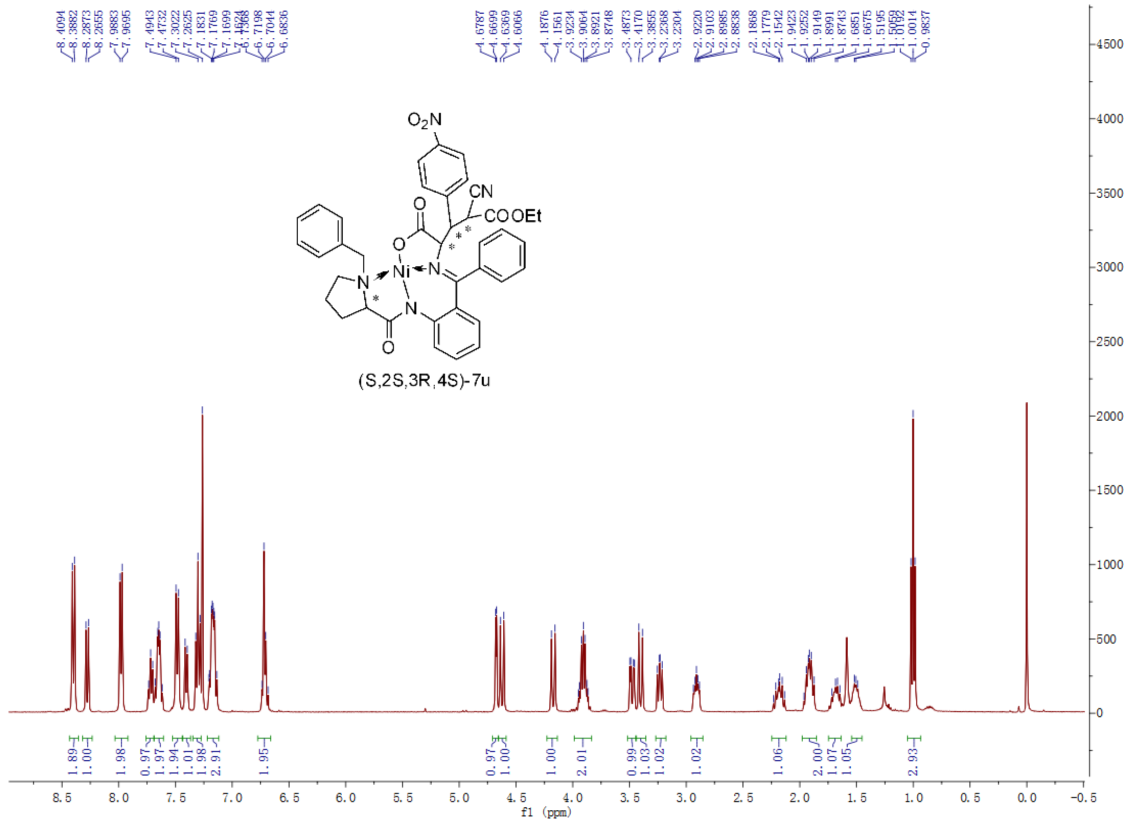

Figure S62. ^{13}C-NMR Spectrum of Compound **7u**.

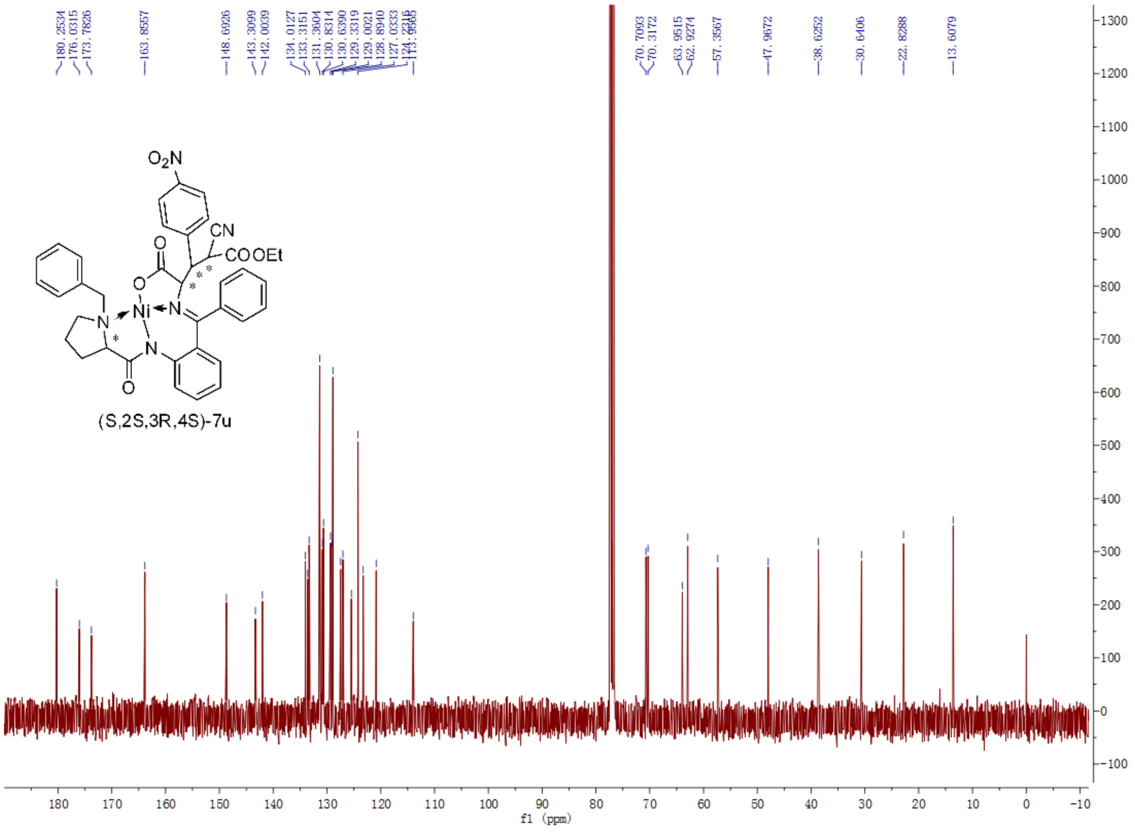

Figure S63. HPLC Spectra of Compound **7u**.

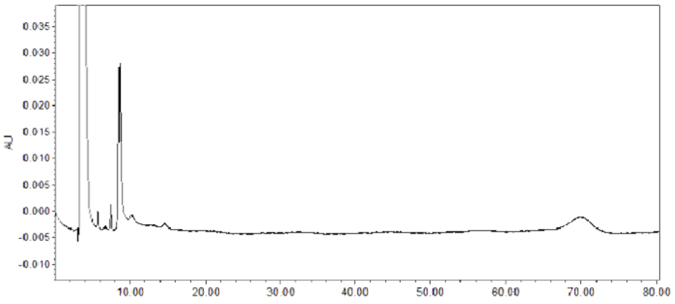

	rt(min)	Area	%Area	Type
1	8.623	270429	50.94	bb
2	70.047	260413	49.06	bb

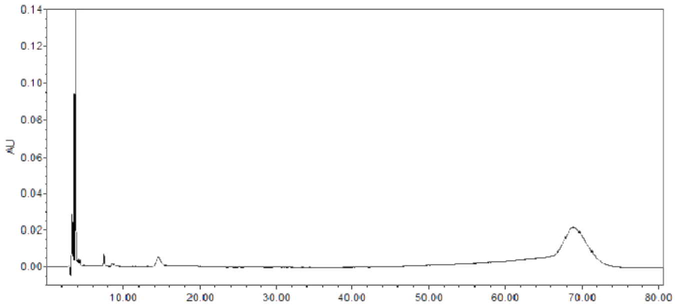

	rt(min)	Area	%Area	Type
1	8.565	42026	1.12	bb
2	68.977	3723841	98.88	bb

Figure S64. ^{1}H-NMR Spectrum of Compound **7v**.

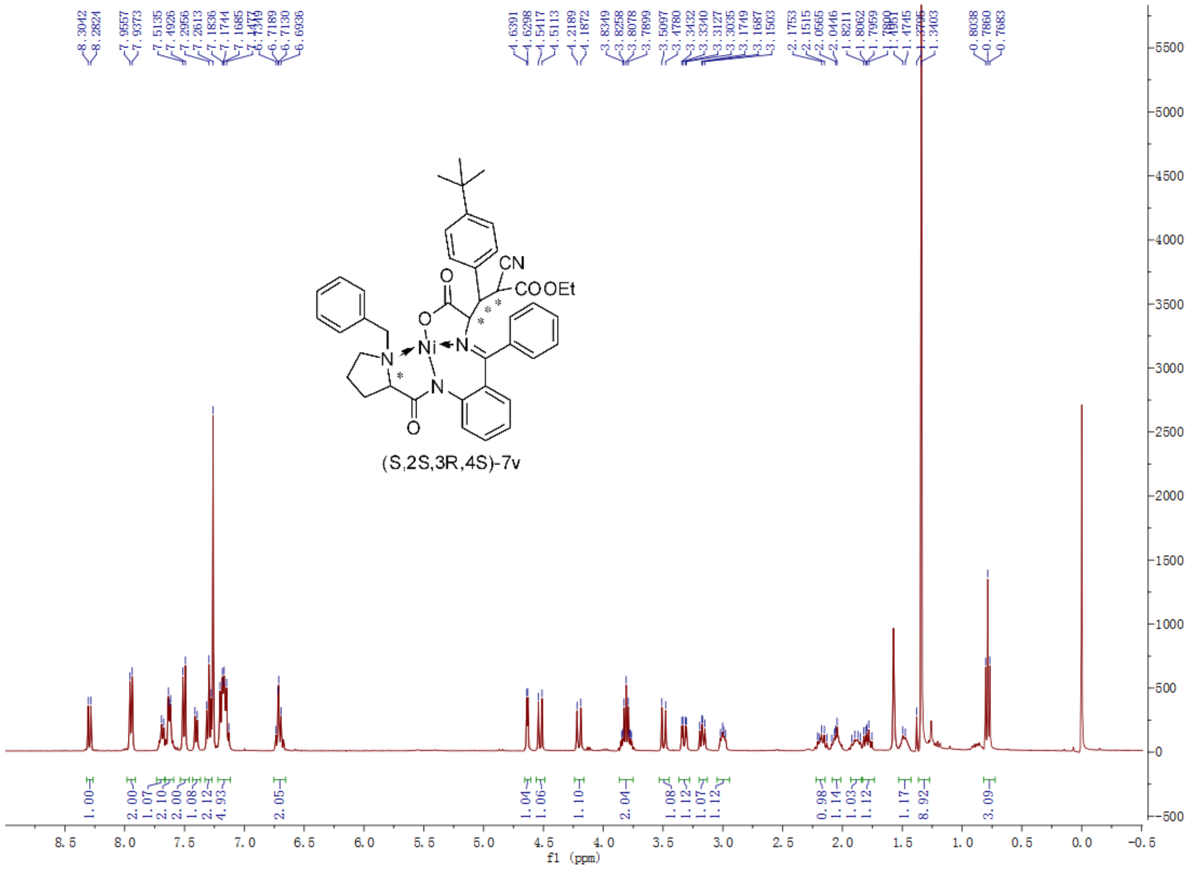

Figure S65. ^{13}C-NMR Spectrum of Compound **7v**.

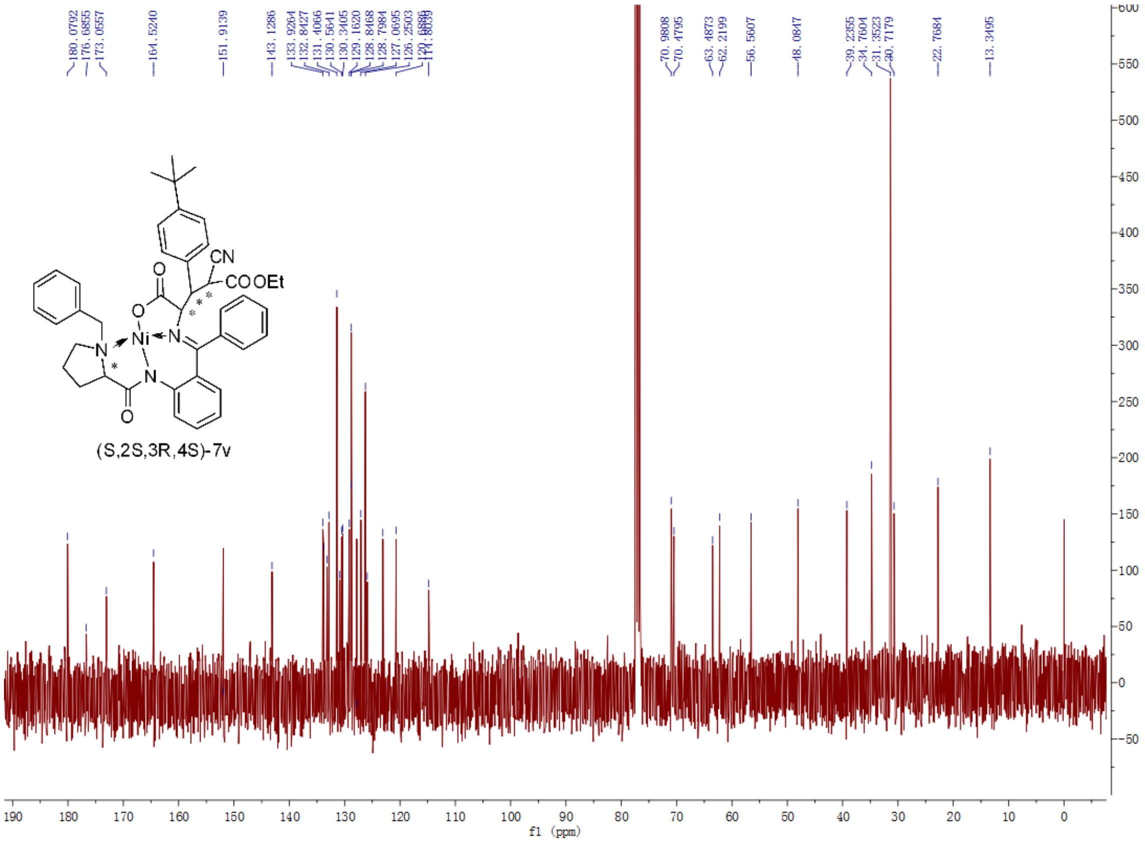

Figure S66. HPLC Spectra of Compound **7v**.

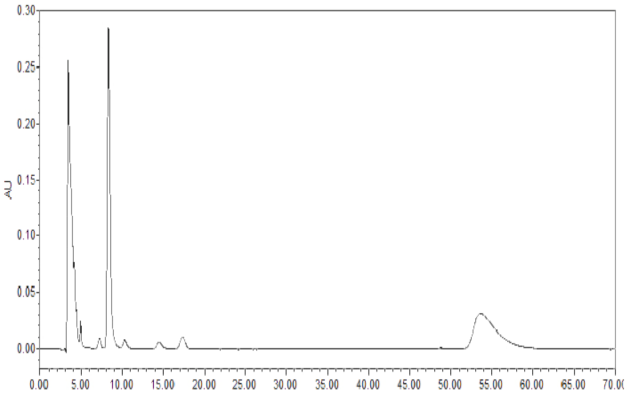

	rt(min)	Area	%Area	Type
1	8.302	6330893	51.10	bb
2	53.628	6057834	48.90	bb

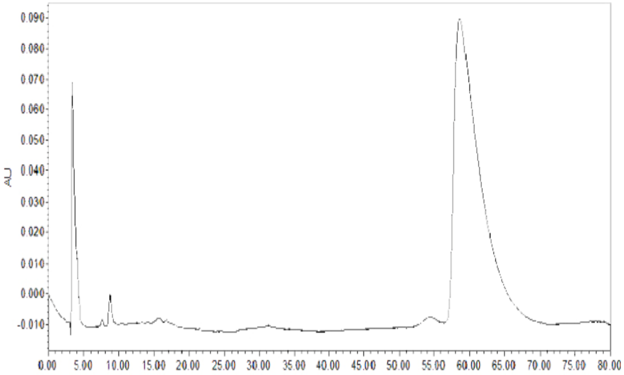

	rt(min)	Area	%Area	Type
1	8.730	263786	1.02	bb
2	58.615	25660622	98.98	bb

Figure S67. ^{1}H-NMR Spectrum of Compound **7w**.

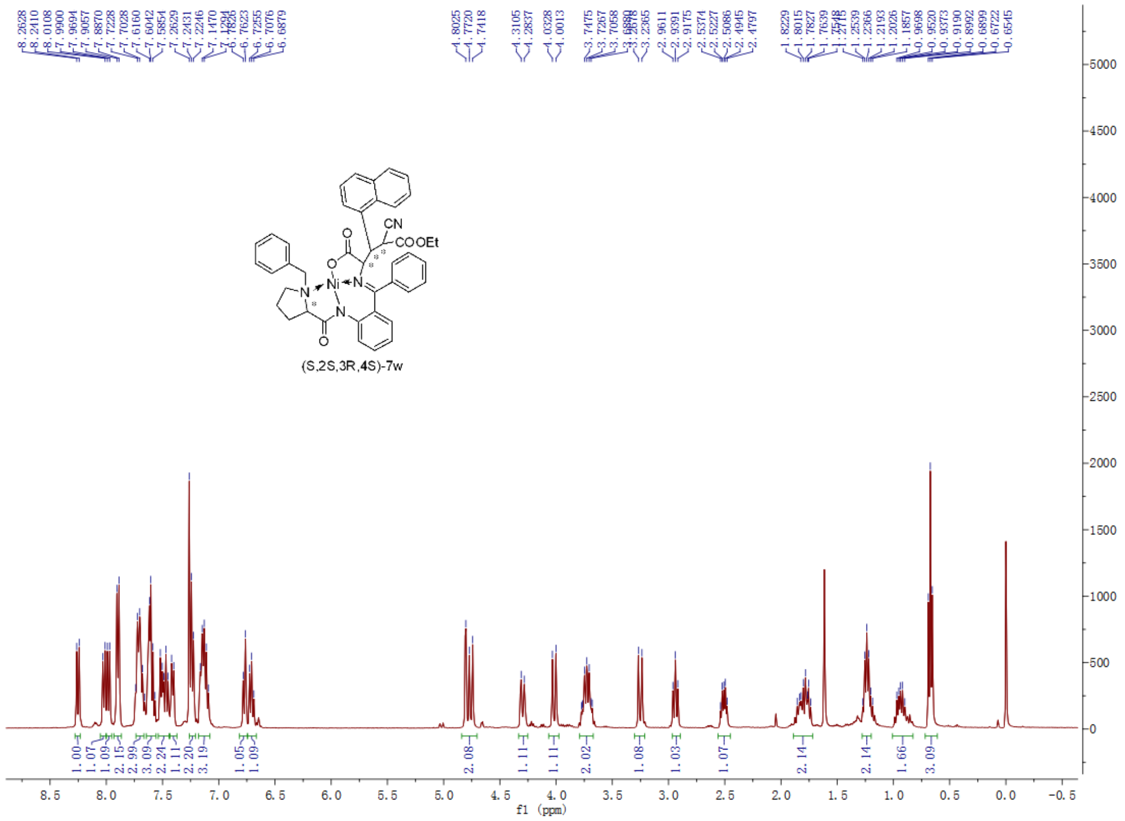

Figure S68. ^{13}C-NMR Spectrum of Compound **7w**.

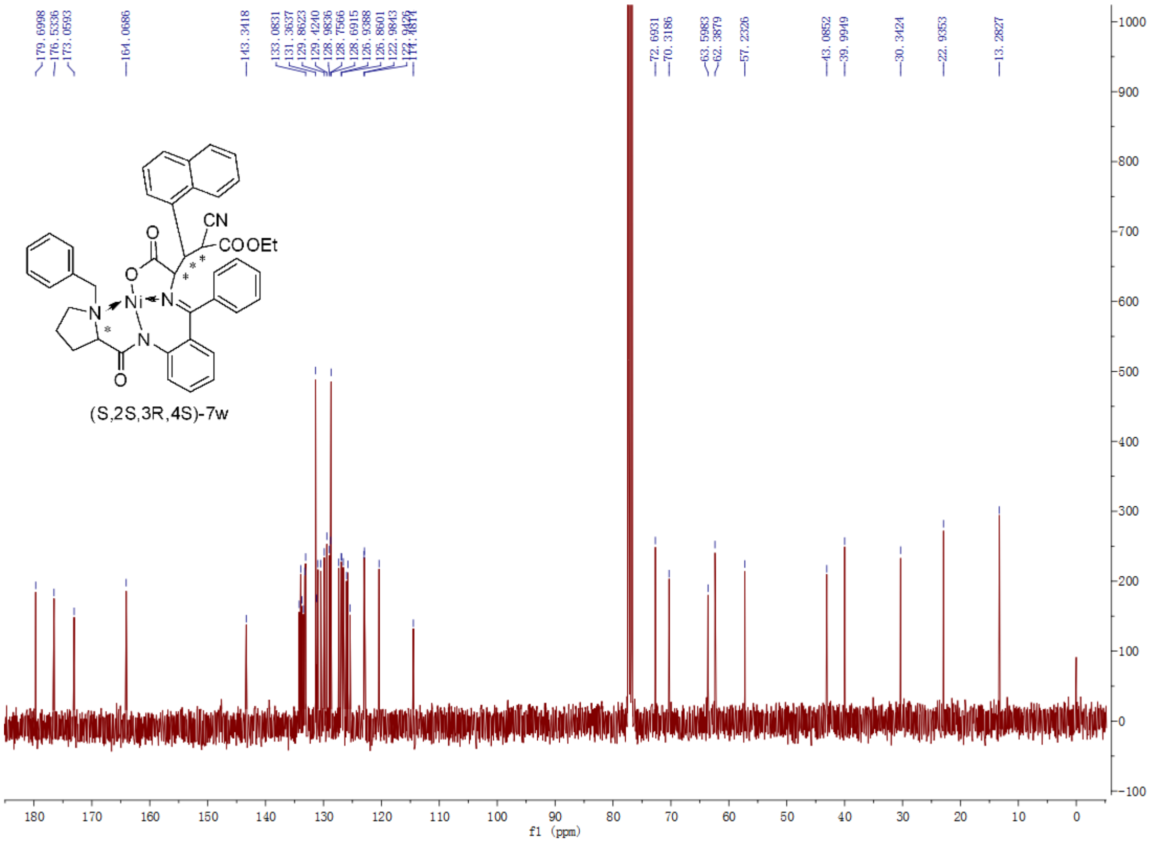

Figure S69. HPLC Spectra of Compound **7w**.

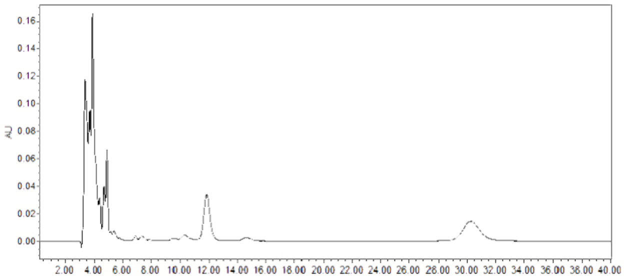

	rt(min)	Area	%Area	Type
1	11.841	1024116	49.21	bb
2	30.272	1056852	50.79	bb

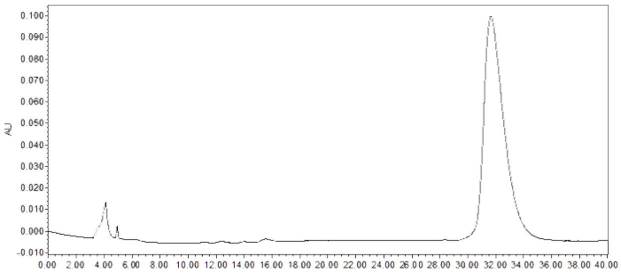

	rt(min)	Area	%Area	Type
1	12.382	14083	0.15	bb
2	31.657	9448407	99.85	bb

Figure S70. ^{1}H-NMR Spectrum of Compound **8a**.

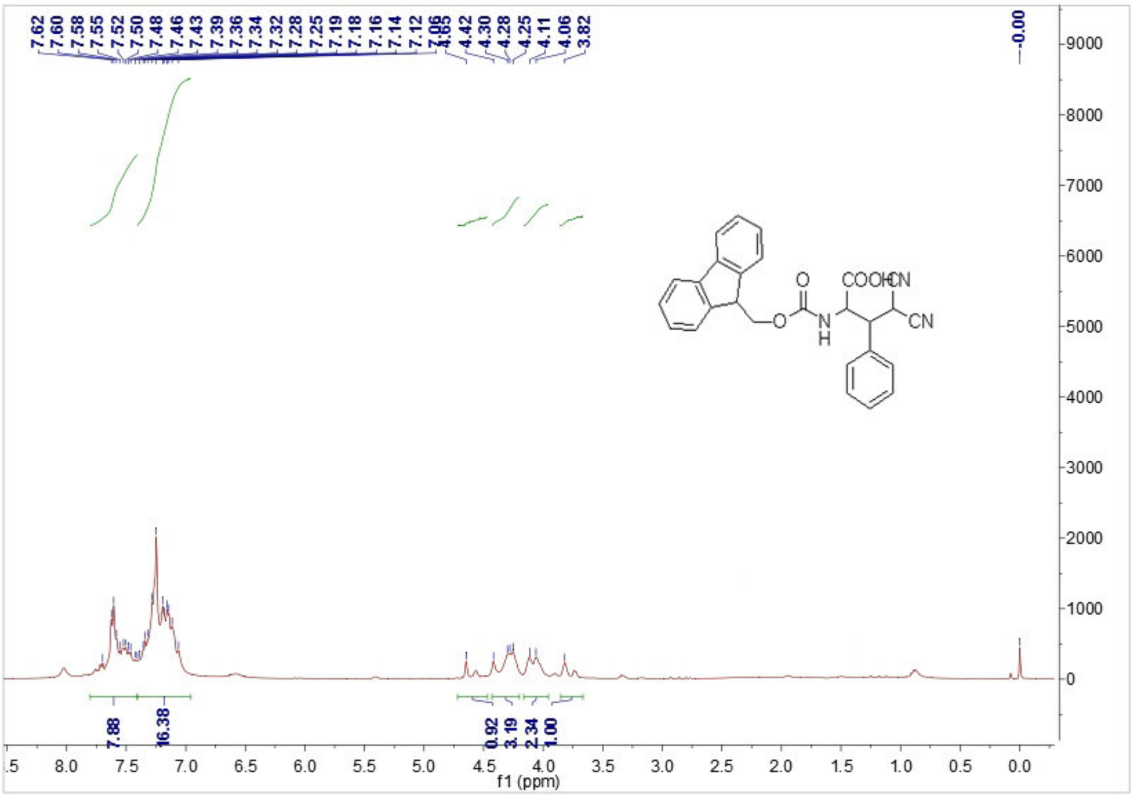

Figure S71. ^{13}C-NMR Spectrum of Compound **8a**.

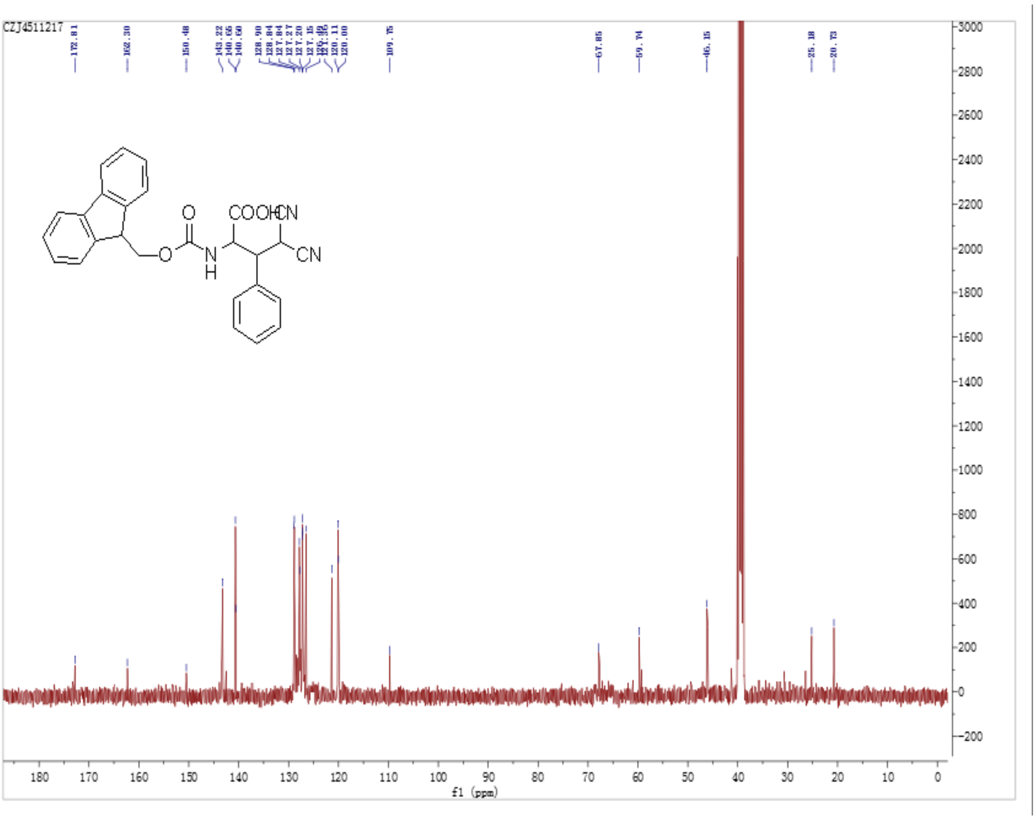

Figure S72. X-ray Single Crystal Structure Analysis of (*S,2S,3R*)-**7a**.

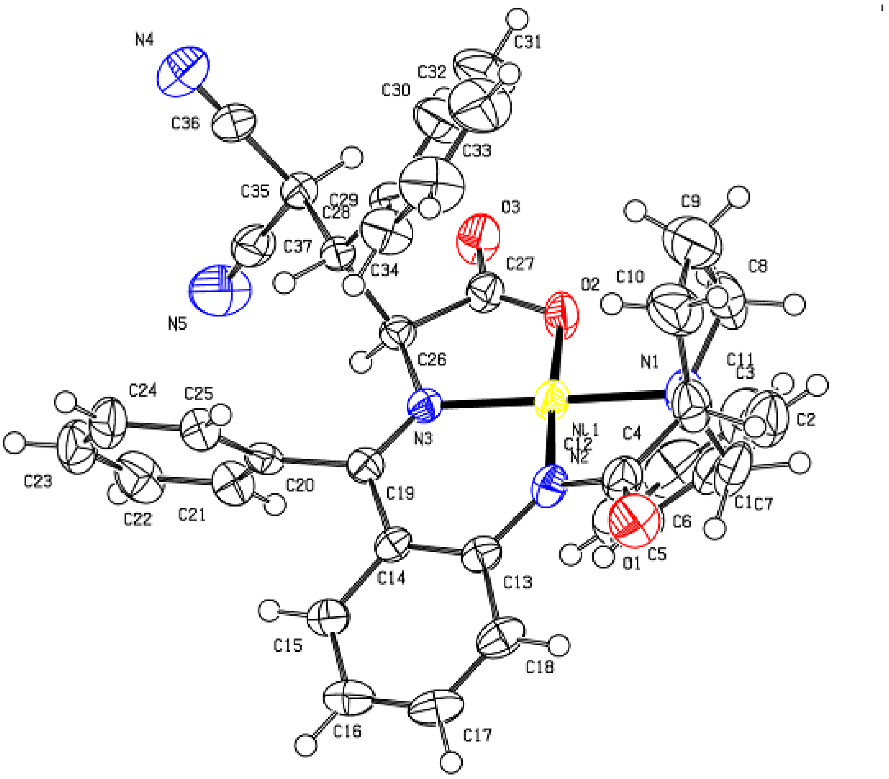

Table S1. Crystal data and structure refinement for **7a**.

**Empirical formula**	**$C_{37}H_{31}N_5NiO_3$**
Formula weight	652.38
Temperature/K	293.15
Crystal system	orthorhombic
Space group	$P2_12_12_1$
a/Å	11.6716(5)
b/Å	14.4772(6)
c/Å	18.6850(7)
α/°	90.00
β/°	90.00
γ/°	90.00
Volume/Å^3	3157.2(2)
Z	4
ρ_{calc}mg/mm^3	1.372
m/mm^{-1}	0.660
F(000)	1360.0
Crystal size/mm^3	0.38 × 0.20 × 0.08
2Θ range for data collection	6.04 to 49.98°
Index ranges	$-13 \le h \le 11$, $-17 \le k \le 13$, $-13 \le l \le 22$
Reflections collected	8050
Independent reflections	5166[R(int) = 0.0237]
Data/restraints/parameters	5166/0/415
Goodness-of-fit on F^2	1.032
Final R indexes [I>=2σ (I)]	R_1 = 0.0388, wR_2 = 0.0649
Final R indexes [all data]	R_1 = 0.0512, wR_2 = 0.0698
Largest diff. peak/hole / e Å^{-3}	0.36/−0.20
Flack parameter	−0.029(12)

Figure S73. X-ray Single Crystal Structure Analysis of (*S,2S,3R,4S*)-**7q**.

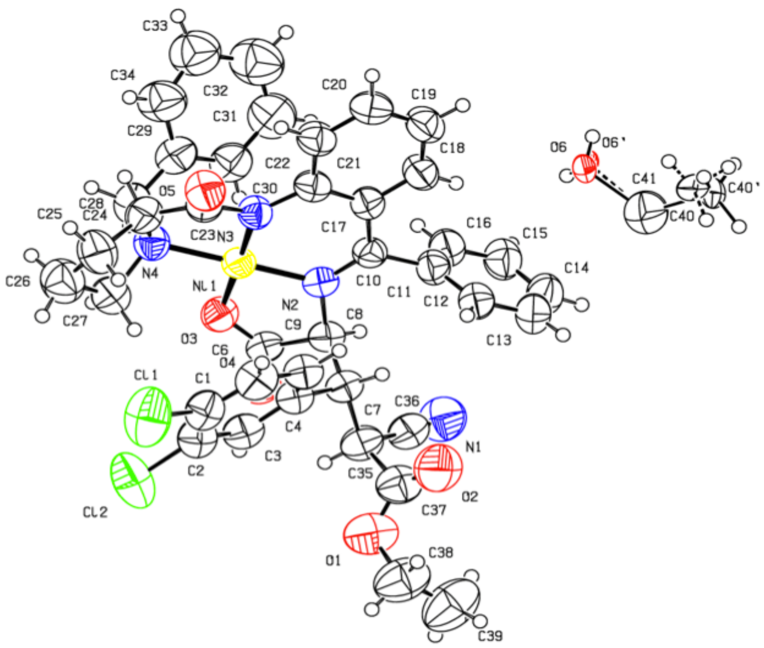

Table S2. Crystal data and structure refinement for **7q**.

**Empirical formula**	**$C_{41}H_{40}Cl_2N_4NiO_6$**
Formula weight	814.36
Temperature/K	293(2)
Crystal system	orthorhombic
Space group	$P2_12_12_1$
a/Å	9.8656(5)
b/Å	17.5526(8)
c/Å	22.7136(9)
α/°	90
β/°	90
γ/°	90
Volume/Å^3	3933.2(3)
Z	4
ρ_{calc}mg/mm^3	1.372
m/mm^{-1}	2.387
F(000)	1688.0
Crystal size/mm^3	0.4 × 0.2 × 0.02
2Θ range for data collection	6.364 to 134.456°
Index ranges	$-8 \leq h \leq 11$, $-20 \leq k \leq 20$, $-27 \leq l \leq 27$
Reflections collected	25555
Independent reflections	7055[R(int) = 0.0855]
Data/restraints/parameters	7055/1686/510
Goodness-of-fit on F^2	1.183
Final R indexes [I>=2σ (I)]	$R_1 = 0.1069$, $wR_2 = 0.2999$
Final R indexes [all data]	$R_1 = 0.1291$, $wR_2 = 0.3355$
Largest diff. peak/hole / e Å^{-3}	1.23/−0.72
Flack parameter	0.090(18)

Crystallographic data (excluding structure factors) for the structures **7a** (CCDC 951535) and **7q** (CCDC 949234) in this paper have been deposited with the Cambridge Crystallographic Data Centre. Copies of the data can be obtained, free of charge, on application to CCDC, 12 Union Road, Cambridge CB2 1EZ, UK (fax: +44 (0)1223 336033 or e-mail: deposit@ccdc.cam.ac.Uk).
